# Heteroaryl iminothioindoxyl (HA-ITI) photoswitches *via* regioselective aza-Wittig synthesis: unifying red-shifted absorption, large *E*/*Z* band separation, and tunable thermal recovery

**DOI:** 10.1039/d5sc08074f

**Published:** 2025-12-24

**Authors:** Jialei Chen-Wu, Carlos Benitez-Martin, José A. González-Delgado, Flip de Jong, Eduard Fron, Gert Steurs, Antonio J. Martínez-Martínez, Francisco Nájera, Morten Grøtli, Johan Hofkens, Joakim Andréasson, Uwe Pischel

**Affiliations:** a CIQSO – Center for Research in Sustainable Chemistry and Department of Chemistry, University of Huelva, Campus de El Carmen s/n E-21071 Huelva Spain uwe.pischel@diq.uhu.es; b Department of Chemistry and Molecular Biology, University of Gothenburg 40530 Göteborg Sweden carlos.benitez-martin@gu.se grotli@chem.gu.se; c Chemistry and Chemical Engineering, Chemistry and Biochemistry, Chalmers University of Technology 41296 Göteborg Sweden a-son@chalmers.se; d KU Leuven, Department of Chemistry Celestijnenlaan 200F B-3001 Leuven Belgium johan.hofkens@kuleuven.be; e KU Leuven, Core Facility for Advanced Spectroscopy Celestijnenlaan 200F B-3001 Leuven Belgium; f KU Leuven, Core Facility for Liquid-State NMR Spectroscopy Celestijnenlaan 200F B-3001 Leuven Belgium; g Departamento de Química Orgánica, Universidad de Málaga, Andalucía-Tech Campus Teatinos s/n Málaga ES-29071 Spain; h Instituto de Investigación Biomédica de Málaga y Plataforma en Nanomedicina–IBIMA, Plataforma Bionand, Parque Tecnológico de Andalucía Málaga ES-29590 Spain; i Max Planck Institute for Polymer Research Ackermannweg 10 55128 Mainz Germany

## Abstract

Herein we introduce heteroaryl-substituted iminothioindoxyl (HA-ITI) photoswitches featuring *N*-heterocyclic moieties based on indole or benzimidazole. A regioselective aza-Wittig reaction of thioisatin, previously unexplored for the synthesis of imino-based photoswitches, enabled the obtention of HA-ITI derivatives and provides an alternative and versatile synthetic access to similar analogues. HA-ITIs undergo blue light-induced *Z* → *E* photoisomerization, followed by very fast thermal *E* → *Z* back isomerization at room temperature (µs to ms timescale; resolved by transient absorption spectroscopy). Irradiation at low temperature (<200 K) provided clear proof for the reversible T-type switching of HA-ITI. This behavior was corroborated by the changes in the UV/vis absorption spectra, with *λ*_abs_ values around 470 nm for the thermodynamically more stable *Z* form and *ca.* 525–550 nm for the metastable *E* isomer. Additional compelling evidence for the *Z* → *E* photoisomerization was obtained by NMR spectroscopy of samples irradiated *in situ* at low temperature. The combination of NMR data, single-crystal X-ray structures, and density functional theory calculations allowed the identification of both inter- and intramolecular interactions (chalcogen and hydrogen bonding), which are present in the *Z* and *E* isomers. Balancing these interactions dictates the differential performance of the switches, resulting in the significant kinetic stabilization, from µs to ms, of the *E* isomer in the benzimidazole-containing photoswitch. These findings establish HA-ITIs as a modular photoswitch platform with highly desirable and tunable photochemical features, thereby broadening the synthetic and conceptual landscape of heteroaryl photoactive systems.

## Abstract 2

Substitution of the iminothioindoxyl core with heteroaryl residues yields HA-ITI photoswitches that exhibit fast T-type behavior, red-shifted visible absorption, and large *E*/*Z* band separation.

## Graphical abstract

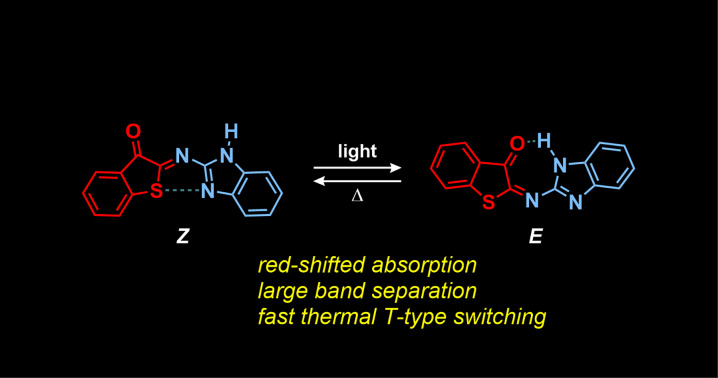

## Introduction

Light as an external stimulus of molecular systems gains its attractiveness from the possibility of spatiotemporal control, *i.e.*, when and where light is applied. In this respect, the use of reversibly actuating photoswitches is regarded as a prime choice. Photoswitches are in many cases toggled between two states, which are commonly characterized by significant differences in their physicochemical properties, such as electronic conjugation, dipole moment, or acid/base behavior, among others. This is observed, for example, in diarylethenes,^1,2^ spiropyrans,^3^ fulgides^4^ or donor–acceptor Stenhouse adducts.^5–7^ These photoswitches operate based on ring opening/closing processes, often implying electrocyclic reactions. However, pronounced structural modification upon photoswitching is more inherent for *E*/*Z* isomerization, with azobenzenes being an archetypal example.^8^ Whether involving electronic variations, geometrical changes, or both simultaneously, the usefulness of photoswitches is evidenced by many applications including molecular systems for data storage and processing,^9–12^ sensing,^13^ imaging,^14–18^ catalysis,^19^ photopharmacology,^20–22^ or light-responsive release.^23–29^ In the particular context of *E*/*Z* photoswitches, the recent years have seen a growing interest in the diversification of this toolbox. This has led to the systematic investigation of hemi(thio)indigo-derived systems^30–33^ and their related Schiff-base variations,^34–37^ hydrazones,^38–44^ or imines.^45–50^ Furthermore, the integration of heterocyclic moieties in *E*/*Z* photoswitches, such as in azaheteroarenes,^51–55^ was shown to be beneficial toward the fine-tuning of optical properties and bistability. However, combining fast T-type thermal recovery with large *E*/*Z* band separation and red-shifted absorption features remains a largely unmet challenge in the design of imine-based photoswitches.

**Fig. 1:**
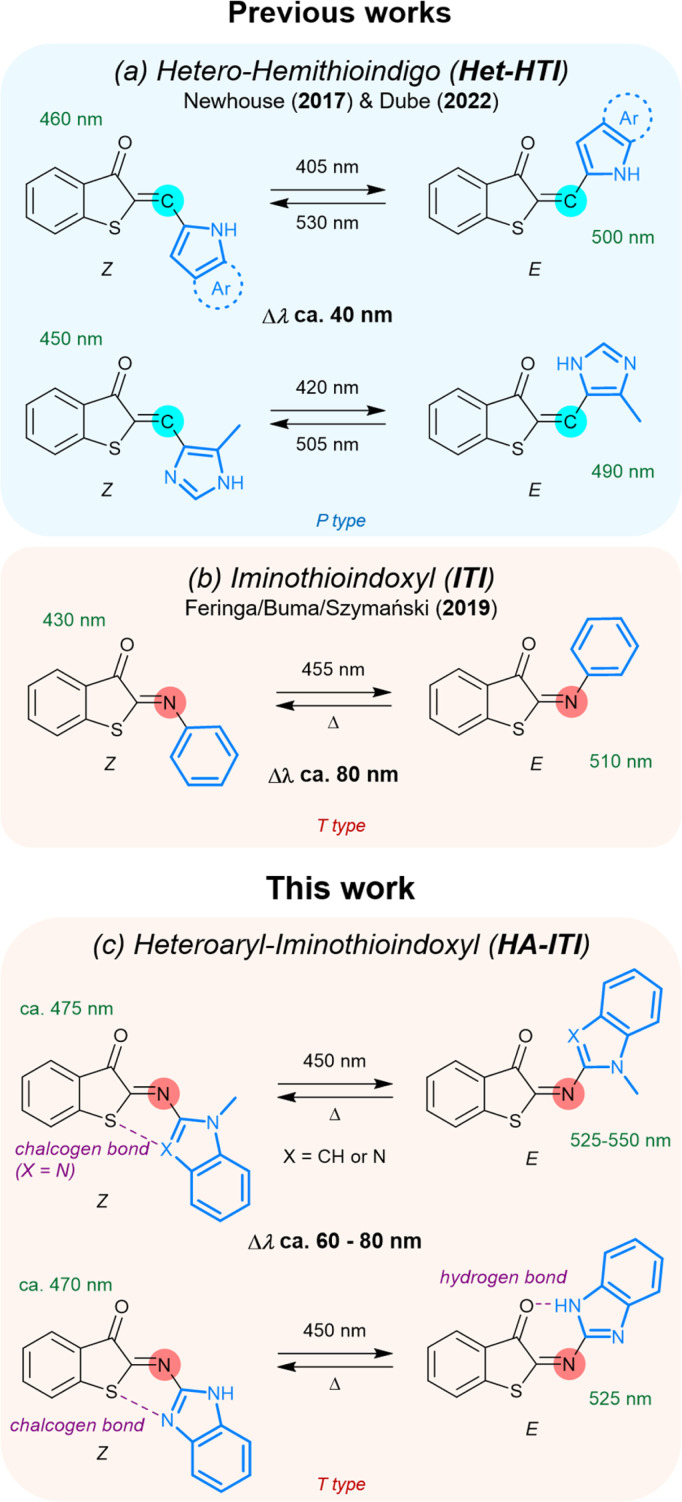
Comparison of heteroaryl-substituted iminothioindoxyl (HA-ITI) with related photoswitches from the previous literature. (a) Hetero-hemithioindigo (Het-HTI) systems reported by the Newhouse (2017) and Dube groups (2022), incorporating various nitrogen-containing heterocycles, behave as P-type photoswitches that can be addressed by blue/green light and the spectral shift between *Z* and *E* isomers (Δ*λ*) is about 40 nm. (b) Iminothioindoxyl (ITI) systems reported by the Feringa/Buma/Szymański groups (2019) feature *Z* → *E* photoisomerization by irradiation at 455 nm and rapid thermal back-isomerization (T-type switching). The band separation between *Z* and *E* isomers (Δ*λ*) is *ca.* 80–100 nm. (c) Light-induced *Z* → *E* photoisomerization of heteroaryl-substituted iminothioindoxyl (HA-ITI) reversible photoswitches. Representative examples show T-type switching behavior triggered by blue light and thermal back-isomerization. Substituents bearing nitrogen-containing heterocycles (*e.g.*, indole, benzimidazole) facilitate supramolecular interactions in the *Z* or *E* isomer, including intramolecular chalcogen bonding (*Z* isomer) and hydrogen bonding (*E* isomer). The photoswitches display pronounced UV/vis absorption shifts upon isomerization (Δ*λ* ≈ 60–80 nm). Wavelengths refer to absorption maxima of the corresponding isomers.

Recent works by the groups of Newhouse^56^ and Dube^31^ are of particular importance for the present investigation (Fig. 1a). They have shown that the integration of *N*-heteroaryl moieties into hemithioindigos (HTI) leads to significant modulation of the reversible switching by photonic stimuli, that is, their P-type photoswitching behavior. The affected photochemical properties include the red-shift of the absorption spectra and the *E*/*Z* band separation (data for selected examples can be found in the SI; Table S1). These attributes enable the application of low-energy photons, which are potentially less harmful in biological environments, and improve the selective addressability of both isomeric forms. In parallel, Feringa, Buma, and Szymański have demonstrated that the aryl-substituted iminothioindoxyl (ITI) platform (Fig. 1b), which is chemically related to the HTI photoswitch family, behaves as a T-type photoswitch (*i.e.*, the back isomerization is thermally activated) with large band separation and fast thermal *E* → *Z* conversion.^34^ Fast thermal recycling is particularly advantageous for applications where real-time responsiveness is targeted by the photoswitches.^57^ Inspired by these seminal findings, we envisaged that installing *N*-heteroaryl residues onto the ITI platform would yield a new class of robust and tunable T-type reversible photoswitches (heteroaryl iminothioindoxyls, HA-ITIs), joining fast thermal switching and substantial *E*/*Z* absorption band separation together with red-shifted spectral features (Fig. 1c). HA-ITIs are accessed by a so far unexplored regioselective aza-Wittig reaction, which circumvents the use of typically unstable nitroso reagents for the incorporation of *N*-heteroaryl units (indole and benzimidazole). This synthetic route additionally unlocks a broader chemical space for imino-based T-type photoswitches and related molecular architectures. Altogether, the herein obtained results establish the HA-ITI platform as a new design paradigm for photofunctional imino-based materials, pairing a synthetically versatile access route with fast, visible-light-responsive, and tunable switching.

## Results and discussion

In the following, we will describe the synthesis and structural characterization of several HA-ITI photoswitches. Evidence of reversible *Z* ⇌ *E* T-type photoswitching is provided by irradiation at low temperatures, monitored by UV/vis absorption and ^1^H NMR spectroscopy. These findings are further supported by transient absorption spectroscopy at room temperature and theoretical calculations.

### Synthesis and characterization of HA-ITI photoswitches

It has been reported that ITI structures can be prepared by two strategies: (a) the base-catalyzed condensation of nitroso derivatives with thioindoxyl^58^ and (b) a [4 + 1] cycloaddition involving 2-mercaptobenzaldehydes and isocyanides.^59^ We focused initially on strategy (a), which had been employed successfully by the Feringa, Buma, and Szymański groups.^34^ However, the reaction with the relatively unstable 2-nitrosoindole was not successful in our case, and only decomposition products were obtained after workup.

**Scheme 1:**
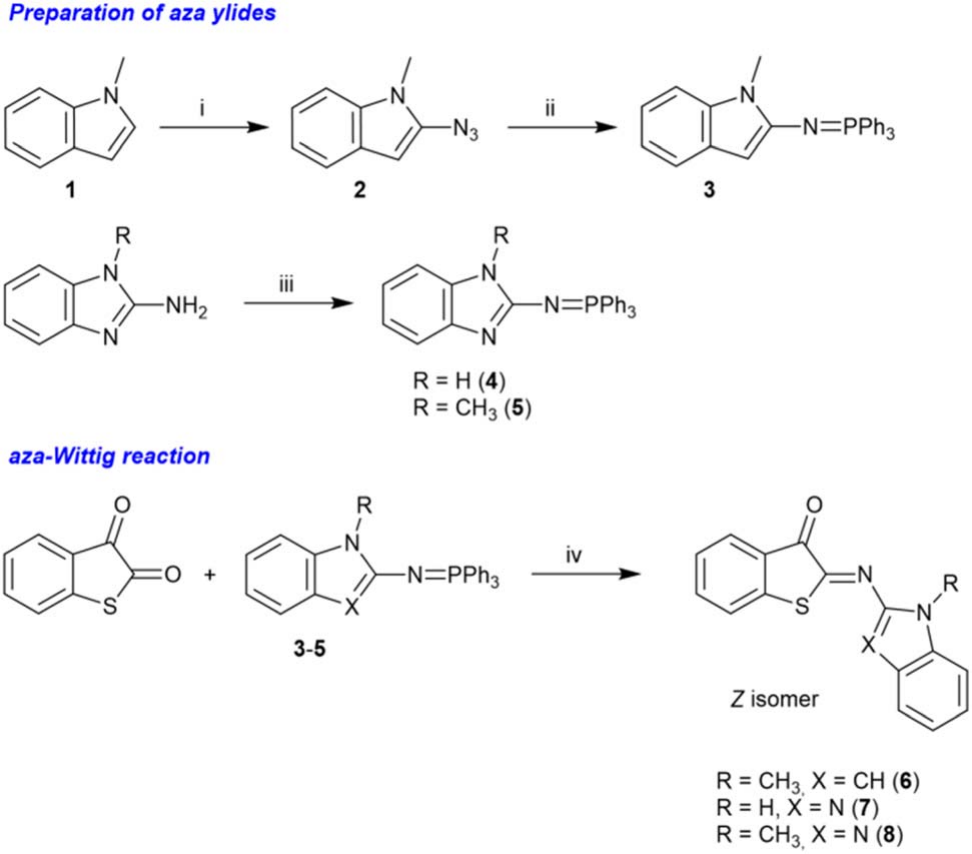
Synthesis of HA-ITI photoswitches 6–8. Reagents and reaction conditions: (i) TsN_3_, *n*-BuLi, Et_2_O, reflux, 70% yield; (ii) PPh_3_, Et_2_O, 0 °C, 30% yield; (iii) Ph_3_PBr_2_, toluene, Et_3_N, reflux, 72–80% yield; (iv) toluene, 90 °C, overnight, 15–65% yield.

Hence, we changed our approach and decided to develop an alternative strategy for preparing the photoswitches 6–8 (Scheme 1). We found that an aza-Wittig reaction between benzo[*b*]thiophene-2,3-dione (thioisatin) and the aza-ylides 3–5 (ref. 60 and 61) (Scheme 1) showed a remarkable selectivity for the reaction at the carbonyl groups neighboring the sulfur atom, providing the desired photoswitches in moderate to good isolated yields (15–65%). The regioselectivity is analogous to the observations made by Jørgensen and Albrecht for conventional Wittig reactions of thioisatin.^62^ Importantly, this regioselective aza-Wittig transformation of thioisatin has not been described before for any photoactive imine system and therefore provides a synthetically distinct entry point compared to previously reported ITI derivatives obtained from nitroso intermediates. Because the final products were obtained in high purity and sufficient quantity, no further efforts were undertaken to optimize the reaction conditions. The final products were characterized by ^1^H and ^13^C NMR spectroscopy as well as by high-resolution mass spectrometry (HRMS). The results confirmed the chemical identity and purity of the compounds (see the SI; Fig. S2–S22).

**Fig. 2:**
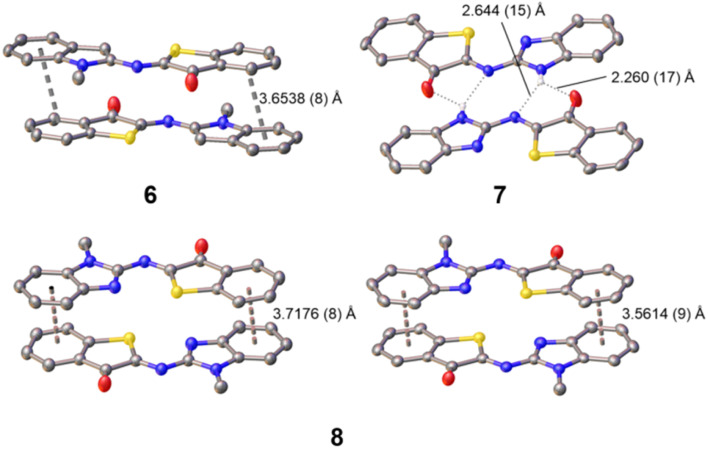
Single-crystal X-ray structures of 6–8 and their intermolecular supramolecular assembly through π-stacking (6 and 8) and H-bonding (7). More details can be found in the SI – Fig. S47–S49 and Tables S5–S7. CCDC: 6 – 2431430, 7 – 2431431; 8 – 2431432).

Furthermore, single crystals of X-ray diffraction quality were obtained by slow diffusion of pentane into a solution of the photoswitch in dichloromethane (6) or in tetrahydrofuran (7 or 8). From the resolved structures (Fig. 2 and S47–S49 in the SI), it can be safely concluded that the thermodynamically stable isomers are of *Z* configuration. This fits with general observations made previously for ITI and Het-HTI photoswitches,^31,34,37,56^ and also matches with the theoretical calculations (see below).

Notably, among the several possible conformers, *Z*-6 crystallized with the methyl group pointing away from the sulfur atom, likely due to steric reasons and the occurrence of a close S⋯H–C (indole) interaction (S⋯H distance of 2.4736 (5) Å). For *Z*-7 and *Z*-8, it is worth noting that the rotamer with the C=N (heterocycle) nitrogen orienting toward the sulfur atom is the one preferred out of all the possible rotamers of the benzimidazole moiety. Both S and N approach closely to a distance of 2.6735 (12) Å (*Z*-7) and 2.7652 (10) (*Z*-8; average for two independent molecules found in the asymmetric unit cell), pointing to the occurrence of S⋯N chalcogen bonding. Similar observations were recently made for an imidazole-derived Het-HTI.^31^ As for *Z*-6, this means that the N–CH_3_ group in *Z*-8 is pointing away from the sulfur, thereby avoiding a steric clash. All three photoswitches show intermolecular interactions. *Z*-7 forms pre-organized “head-to-tail” dimers, held together by complementary intermolecular hydrogen bonds of N–H with O=C [O⋯H distance of 2.260 (17) Å] and N=C [N⋯H distance of 2.644 (15) Å], but no further higher-order organization by π–π stacking is observed. Finally, the nearly planar *Z*-6 and *Z*-8 form π–π stacked “head-to-tail” dimers with an *anti* orientation of the carbonyl groups. In fact, for *Z*-8 two non-equivalent π–π dimers with slightly different packing are observed (Fig. 2). The inter-planar distance is *ca*. 3.6–3.7 Å. It should be noted that none of the dimers propagates further, and no π-chains are formed.

### UV/vis absorption spectroscopy of the *Z* isomer

**Fig. 3:**
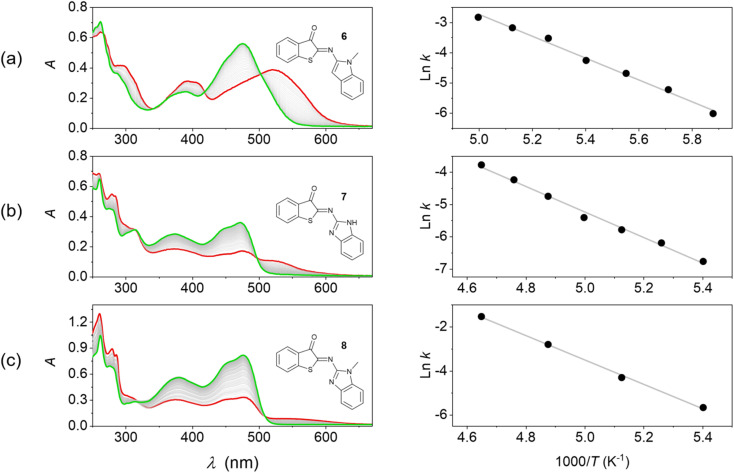
Thermal *E* → *Z* isomerization of HA-ITI photoswitches monitored by UV/vis absorption spectroscopy and Arrhenius analysis. (Left) Spectra of the thermal back isomerization of (a) 6 (at 170 K), (b) 7 (at 185 K), and (c) 8 (at 185 K) in MTHF. The green spectra correspond to the fully recovered signatures of the *Z* forms, while the red spectra correspond to the signature of the photothermal stationary state mixture (generated by irradiation at 375 nm). (Right) Corresponding Arrhenius plots.

The HA-ITI photoswitches 6–8 in their *Z* form are characterized by a long-wavelength absorption band with a maximum at around 470 nm (Table 1 and Fig. 3). It is noteworthy that HA-ITI photoswitches show a considerable red-shift of about 40 nm compared to the ITI analogues.^34^ This hints at the involvement of the heterocyclic moiety in the molecular orbitals participating in the transition, which was verified in the theoretical calculations (see below). Based on the structural analogy with ITI photoswitches the long-wavelength absorption band is ascribed to a π, π* transition.^34^ As verified for *Z*-7, the spectral position of the absorption maximum is not significantly dependent on the solvent, featuring minor shifts no larger than 8 nm (*λ*_max_: 467 nm in acetonitrile and 475 nm in DMSO; the corresponding spectra can be found in Fig. S23 in the SI).

**Table 1:** Spectral properties and thermal back isomerization of the photoswitches 6–8 in MTHF

	*λ*_abs_ (nm) *Z*^a^ [*ε* (M^−1^cm^−1^)]	*λ*_abs_ (nm)		*τ* (ms) *E* → *Z*^d^	*E*_a_ (kJ mol^−1^)	
		*E* b	*E* c		*E* → *Z*^e^	*E* → *Z*^f^
6	474 [20200]	525	∼545	0.73	30.1	59.1
	444 [14400]					
	389 [7400]					
7	472 [16100]	525	530	23.6	33.0	43.6
	450 [14100]					
	379 [10400]					
8	476 [16400]	550	∼560	0.67	46.2	51.4
	454 [14500]					
	381 [10600]					

a Absorption maximum of the *Z* isomer, determined by conventional UV/vis absorption spectroscopy in MTHF.

b Absorption maximum of the *E* isomer, determined by low-temperature UV/vis absorption spectroscopy.

c Absorption maximum of the *E* isomer, determined by transient absorption spectroscopy.

d Time constant for the *E* → *Z* thermal back isomerization at 298 K, determined by transient absorption spectroscopy in MTHF.

e Arrhenius activation energy for the *E* → *Z* thermal back isomerization in MTHF; extracted from low-temperature irradiation experiments with UV/vis absorption monitoring.

f Arrhenius activation energy for the *E* → *Z* thermal back isomerization in tetrahydrofuran-*d*_8_ (THF-*d*_8_); extracted from low-temperature irradiation experiments with monitoring by ^1^H NMR spectroscopy.

### Photoswitching monitored by UV/vis absorption spectroscopy at low temperature

In a first attempt to test the photoswitching function of *Z*-6, *Z*-7, and *Z*-8, their 2-methyltetrahydrofuran (MTHF) solutions were subjected to irradiation with light at *λ* > 455 nm at room temperature. However, no changes were observed in the absorption spectra under such conditions. It is well known that imine-based photoswitches can undergo thermal back isomerization due to the possibility of inversion at the imine nitrogen atom, which was also observed for the ITI photoswitch platform.^34^ If the back isomerization is very fast at room temperature, this can explain the lack of observation of the *E* isomer under conventional experimental conditions. Therefore, we resorted to irradiation experiments (*λ*_exc_ = 375 nm) at low temperature (*T* < 220 K in MTHF). Indeed, under these conditions the thermally activated *E* → *Z* isomerization was slowed down sufficiently as to allow the observation of photoswitching (Fig. 3).

In all cases the long-wavelength absorption of the *Z* form at *ca.* 470 nm diminished and new bathochromically displaced spectral features at wavelengths above 500 nm were detected, which are attributed to the formation of the corresponding *E* isomer. A substantial band separation of the *Z* and *E* isomers is a property that is often sought after, as it facilitates photoisomerization reactions in mainly one direction. The previously reported ITI photoswitches reach significant values of 80–100 nm,^34^ while our HA-ITI photoswitches feature a comparable band separation of 60–80 nm.

The spectral changes reverse in the dark and are characterized by several well-defined isosbestic points (Fig. 3), confirming the uniformity of the *E* → *Z* back isomerization process. Monitoring the kinetics and fitting them to monoexponential decays revealed lifetimes of several hundreds of seconds at low temperature (*e.g.*, 414 s at 170 K for *E*-6, 885 s at 185 K for *E*-7, and 300 s at 185 K for *E*-8); Fig. S24–S38 in the SI.

The Arrhenius analysis of the temperature-dependent kinetic data revealed activation energies of 30.1, 33.0, and 46.2 kJ mol^−1^ for the *E* → *Z* isomerization of 6, 7, and 8, respectively. These numbers are in broad agreement with observations made for ITI photoswitches (*E*_a_ ∼55–60 kJ mol^−1^).^34^ Albeit associated with a large error, the extrapolation of these data provides a rough estimate of the expected timescale at room temperature on the order of tens of milliseconds. This is in accordance with the lack of observation of macroscopic conversion in conventional irradiation experiments at 298 K. More accurate kinetic data for the *E* → *Z* thermal back isomerization at room temperature were obtained from transient absorption studies and are shown in Table 1.

### Study of the photoswitching by ^1^H NMR spectroscopy at low temperature

Having established the T-type photoswitching of HA-ITI by means of monitoring the changes in the UV/vis absorption spectra, we attempted to gain complementary insights by performing ^1^H NMR spectroscopic characterization of HA-ITI *E*- and *Z*-isomers at low temperature. For this purpose, samples of the HA-ITI photoswitches in THF-*d*_8_ were irradiated at 450 nm directly in the NMR tube at temperatures of 180 K, 190 K, and 200 K. It is worth emphasizing that the compounds feature only a few diagnostic protons that could help to unambiguously establish the occurrence of the *Z* → *E* photoisomerization. However, in the case of HA-ITI photoswitch 6, the proton at the 3-position of the *N*-methylindole moiety offers a unique opportunity for *E*/*Z* assignment.

**Fig. 4:**
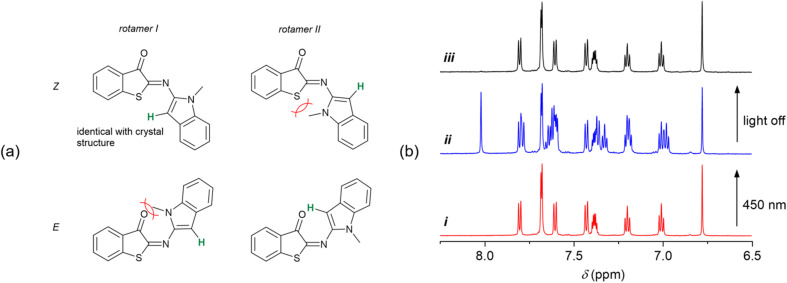
(a) Possible rotamers of the *Z* and *E* forms of HA-ITI photoswitch 6. The proton highlighted in green was used as diagnostic tool for rationalizing the formation of the *E* isomer upon irradiation. (b) Partial ^1^H NMR spectra of 6 in THF-*d*_8_ at 190 K, demonstrating the reversible T-type photoswitching. (i) *Z*-6 before irradiation, (ii) photothermal stationary state upon irradiation with 450-nm light (*Z*/*E ca.* 50/50), (iii) spectrum after thermal back isomerization in the dark, corresponding to *Z*-6.

In Fig. 4a, the structures of the *Z* form and the metastable *E* form of 6 are shown. Each of the configurational isomers may exist as rotamer I or II, among many other possible conformations. A simple visual inspection of the structures indicates that steric clashes involving the methyl group will likely disfavor rotamer II of the *Z* form and rotamer I of the *E* form. Hence, on the one hand, *Z*-6 is expected to be present as rotamer I, as it is the case in the solid-state structure that was established by X-ray crystallography (Fig. 2). On the other hand, the photogenerated *E*-6 is more likely present as rotamer II, where the proton at the 3-position of the *N*-methylindole part is close to the carbonyl group, lying in the nodal plane of the C=O. This should yield a pronounced deshielding of this proton.^63^

Importantly, these intuitively derived conformational preferences of photoswitch 6 are confirmed by the NMR experiment at 190 K. In Fig. 4b, the aromatic region of the ^1^H NMR spectra of HA-ITI 6 is shown (i) before, (ii) during continuous irradiation at 450 nm, and (iii) after switching off the light source. The signal at 6.78 ppm in the spectrum of *Z*-6 is ascribed to the proton at the 3-position of the *N*-methylindole moiety. Upon irradiation significant changes are observed, of which the most indicative is the emergence of a signal at 8.03 ppm. This signal is assigned to the aforementioned proton in the *E* form of the photoswitch. This pronounced deshielding by 1.25 ppm would only be expected in the *E* form of 6, thereby lending strong support for the *Z* → *E* photoisomerization. The integration of the signals yields *ca.* 50% *Z* → *E* conversion in the photothermal stationary state at 190 K. As expected, this value is even more favorable at lower temperature (180 K), *i.e.*, *ca.* 78% *Z* → *E* conversion. For a higher temperature (200 K) the considerably faster thermal back isomerization yields a much lower *Z* → *E* conversion of *ca.* 10%; Fig. S40 in the SI. Upon irradiation of the other two HA-ITI photoswitches 7 and 8, pronounced changes were observed in their ^1^H NMR spectra; Fig. S41–S46 in the SI. However, the unambiguous assignment of these changes to the formation of the corresponding *E* isomers proved to be challenging, especially for HA-ITI 8. In the case of compound 7, the NH proton was considered a potential diagnostic marker. Interestingly, irradiation of *Z*-7 induced a notable upfield shift of this signal, from 12.79 to 11.67 ppm (Fig. S43 in the SI), which is contrary to expectations based on proximity to the nodal plane of the carbonyl π orbital or involvement in N–H⋯O=C hydrogen bonding in the *E* isomer.

This unexpected behavior may be rationalized by steric constraints and less ideal alignment of the NH in *E*-7 and/or the initial presence of an intermolecular hydrogen-bonded dimer of the *Z* isomer, analogous to the arrangement observed in the solid-state structure of *Z*-7. In such dimer the NH proton signal may be more downfield shifted than in the case of intramolecular hydrogen bonding of NH in *E*-7. Despite the absence of definitive spectroscopic markers for *E*-7 and *E*-8, the robust evidence for *Z* → *E* photoisomerization in the structurally related HA-ITI 6 strongly suggests the occurrence of an analogous transformation in compounds 7 and 8. The photothermal stationary state distribution at 190 K points to significant *Z* → *E* photoisomerization for 7 (35%) and near-quantitative conversion for 8 (94%); data at other temperatures can be found in Tables S3 and S4 in the SI. It should be noted that a direct comparison of the conversion yields between the different photoswitches is complicated by the difficulty to guarantee exactly equal irradiation geometries and conditions in our setup (Fig. S1 in the SI). Importantly, all photoswitches demonstrated complete thermal reversibility upon cessation of irradiation (Fig. 4b).

### Transient absorption spectroscopy (TAS)

**Fig. 5:**
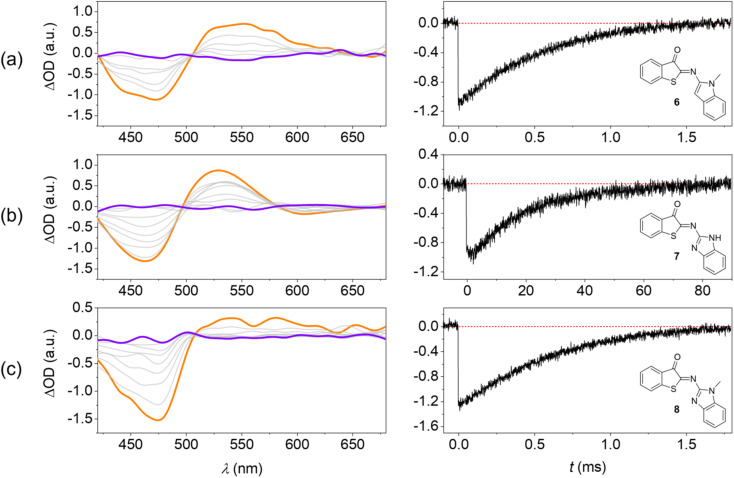
Transient absorption spectroscopy confirms fast thermal *E* → *Z* isomerization of HA-ITI photoswitches at room temperature. (Left) Transient absorption spectra of (a) 6, (b) 7, and (c) 8 in MTHF at room temperature; excitation at 355 nm, orange: spectrum after laser flash, purple: spectrum after complete ground-state recovery. (Right) Recovery kinetics of the *Z* form at 460 nm.

TAS at the micro- to millisecond timescale enabled monitoring of the thermal *E* → *Z* back isomerization at room temperature (Fig. 5). These experiments were performed in MTHF for a better comparison with the low-temperature steady-state irradiations (Fig. 3).

The photoswitches show two transients: (i) a negative signal that coincides with the inverted long-wavelength absorption band in the 400–500 nm range and (ii) a positive signal in the 500–650 nm spectral window. The spectra cross at an isosbestic point at *ca.* 500 nm. On the one hand, signal (i) is unambiguously assigned to the ground-state bleaching (GSB) of the *Z* isomer. On the other hand, the spectral fingerprint of the transient (ii) coincides with the corresponding changes in the low-temperature irradiations and is therefore assigned to the *E* isomer.

Notably, the lifetime of the *E* isomer (*τ*_*E*→*Z*_) in the thermal *E* → *Z* back isomerization at room temperature is readily determined from the recovery kinetics of the GSB (Fig. 5). For the photoswitches 6 and 8 this value was obtained as 730 µs and 670 µs, respectively. For HA-ITI 7, a decay two orders of magnitude slower is observed (*τ*_*E*→*Z*_ = 23.6 ms). The reason for this significant difference is assigned to the stabilization of *E*-7 by intramolecular N–H⋯O=C hydrogen bonding. It is noteworthy that ITI photoswitches typically display lifetimes of tens of milliseconds for the *E* form. Recent attempts to control the kinetics of the thermal back-isomerization by means of electronic factors have shown that longer lifetimes (close to 100 ms) can be achieved.^37^ However, faster switching reaching the sub-millisecond regime was not attained by this strategy. Interestingly, the HA-ITI platform provides such characteristics, which may be particularly useful in applications where very fast turnover is an advantage. In addition, by drawing on tailored supramolecular interactions, such as in *E*-7, the lifetime can be tuned at will. Likely, the combination with electronic factors would allow for further extension of the lifetime range.

Unfortunately, the considerable spectral overlap of the *E* isomer with the *Z* isomer hampers the accurate determination of the quantum yield of the *Z* → *E* transformation of the HA-ITI photoswitches 6–8. However, it is certainly not unreasonable to assume similar efficiencies as observed for ITI^34^ or Het-HTI^56^ switches.

### Theoretical calculations

To obtain additional insight into the relative stability of the *Z* and *E* isomers and their optical properties, density functional theory (DFT) calculations were performed. The ground-state structures were optimized at the M06-2X/6-311+G(d,p) level,^64,65^ including the solvation model based on density (SMD)^66^ for MTHF. This combination of exchange-correlation functional and solvation model was employed to describe ITI derivatives, based on its good accuracy for thermochemistry.^34,37^ For all investigated HA-ITI photoswitches 6–8, the *Z* isomer is more stable than the *E* isomer by approximately 21.3–25.9 kJ mol^−1^. This configurational preference is in full agreement with the observations made for the X-ray crystallographic structures (Fig. 2). The *Z* isomers of all the photoswitches are planar, as well as the *E* isomers of 6 and 7. For the *E* isomer of compound 8 a distorted structure with a dihedral angle of 110° between the ITI and benzimidazole ring systems was identified.

**Fig. 6:**
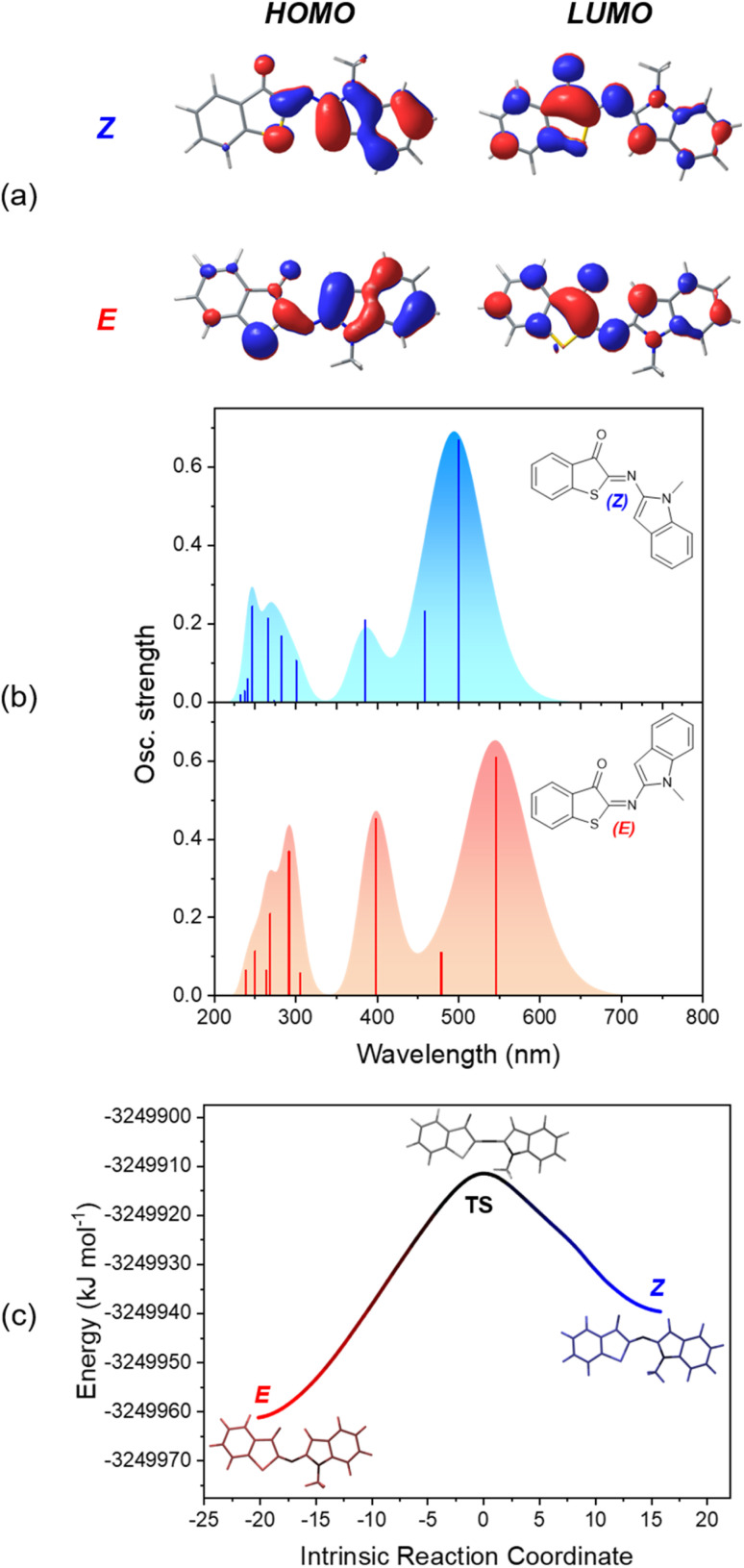
(a) HOMO and LUMO density plots for *Z*- and *E*-6. (b) Calculated absorption spectra of both isomers (*Z*-6: blue, *E*-6: red). (c) Calculated *E* → *Z* ground-state isomerization path for photoswitch 6. Note that the herein obtained rotamer of the *Z* isomer easily converts to the structure that was obtained for the crystal by rotation around the C–N σ bond (activation energy of *ca.* 3.8 kJ mol^−1^; Fig. S56 in the SI).

The optical properties of the *Z* and *E* isomers in MTHF (Table 2) were calculated by means of time-dependent density-functional theory [SMD(MTHF)/*m*PW1PW91/6-311+G(d,p)].^67^ The S_0_ → S_1_ transition, corresponding to the lowest-energy absorption band, implies mainly the frontier molecular orbitals (FMOs) HOMO and LUMO. As a general observation, the HOMO is preferentially located on the *N*-heterocyclic moiety, while the LUMO can be found on the thioindoxyl residue (Fig. 6a, S50 and S52 in the SI). The calculated energies for the S_0_ → S_1_ transition are in very good agreement with the experimental values, deviating maximally by 0.14 eV. It is worth noting that, as observed for ITIs, the photophysical properties of our derivatives are not significantly dependent on the solvent (see above) despite featuring an intermediate, in some cases pronounced, charge-transfer excited state. This notion suggests that HA-ITIs are not subjected to the classical limitations of time-dependent density-functional theory, in analogy to ITIs.^34,68^ Thus, the use of the *m*PW1PW91 functional, having a certain exchange-correlation character and better reproducing the experimental results, was preferred.

**Table 2:** Calculated optical spectroscopic properties of the *Z* and *E* isomers of 6–8

	Oscillator strength *f*	FMO contribution	*E*_calc_ (eV) (S_0_ → S_1_)	*E*_exp_ (eV) (S_0_ → S_1_)
*Z*-6	0.6682	HOMO → LUMO (99%)	2.48	2.62
*E*-6	0.6097	HOMO → LUMO (99%)	2.27	2.27
*Z*-7	0.5699	HOMO → LUMO (92%)	2.62	2.63
*E*-7	0.5146	HOMO → LUMO (95%)	2.28	2.34
*Z*-8	0.5932	HOMO → LUMO (93%)	2.60	2.60
*E*-8	0.0924	HOMO → LUMO (93%)	2.10	2.21

The data confirm the experimental notion that the HA-ITI platform functions as a positive photoswitch, with the thermodynamically less stable *E* isomer absorbing at longer wavelengths than the *Z* isomer (Fig. 6b, S51 and S53 in the SI). Notably, the comparatively small calculated oscillator strength of the *E* isomer of compound 8 accounts for its relatively weak absorption signal observed in both TAS and low-temperature irradiation experiments (Fig. 3 and 5).

Finally, the reaction path for the thermal *E* → *Z* isomerization was mapped [SMD(MTHF)/M06-2X/6-311+G(d,p)]; Fig. 6c for photoswitch 6 and Fig. S54 and S55 in the SI for data for 7 and 8. The intrinsic reaction coordinate is correlated with the change of the C=N–C inversion angle. In the transition state an angle of *ca.* 175–180° is observed for all three photoswitches. These calculations enabled us to extract activation energies for the *E* → *Z* isomerization, which were found to be in qualitative agreement with the experimentally determined values (Table 1): 49.8 kJ mol^−1^ for 6, 57.4 kJ mol^−1^ for 7, and 49.0 kJ mol^−1^ for 8. It is noteworthy that the calculated activation energies reflect also the enhanced lifetime of the *E*-7. This was not observed for the experimentally determined values, which are subjected to a higher uncertainty.

## Conclusions

The integration of *N*-heterocyclic moieties, such as indoles or benzimidazoles, with iminothioindoxyl (ITI) photoswitches enables the pinpointed spectral tuning of the absorption properties of the *Z* and *E* forms, while maintaining the T-type character with fast thermal back isomerization. Specifically, the examples of heteroaryl iminothioindoxyl (HA-ITI), which were developed in this work and prepared by a so far unexplored aza-Wittig reaction strategy, show significantly red-shifted absorption spectra compared to ITI or hemithioindigo photoswitches. Importantly, a separation of the *E*/*Z* absorption bands of 60–80 nm was observed. Irradiation of the thermodynamically stable *Z* form with blue light generates the metastable *E* form, which reverts on a µs to ms timescale in a thermally activated process with relatively low activation energies (30–60 kJ mol^−1^). The occurrence of intraswitch supramolecular interactions such as N⋯S chalcogen bonding and C=O⋯H–C as well as C=O⋯H–N hydrogen bonding interactions dictates switch-specific variations of performance characteristics. Especially interesting is the case of photoswitch 7, in which C=O⋯H–N interaction occurs in the metastable *E* form. This enabled us to design a switch that shows ms thermal recovery of the *Z* isomer as opposed to the µs time scale for 6 and 8. To our knowledge, HA-ITIs are the first class of imine-based photoswitches to exhibit both fast T-type switching and a red-shifted, strongly resolved *E*/*Z* absorption profile in the visible range. These results not only expand the design space of imine-based photoswitches, but also establish HA-ITIs as promising candidates for integration into light-controlled molecular devices, optical logic circuits, or responsive therapeutic systems, among others.

## Author contributions

J. C.-W.: data curation, formal analysis, investigation, methodology, validation, visualization, writing – review & editing. C. B.-M.: conceptualization, data curation, formal analysis, investigation, methodology, validation, visualization, writing – review & editing. J. A. G.-D.: formal analysis, investigation, methodology, visualization, writing – review & editing. F. d. J.: formal analysis, investigation, methodology, visualization, writing – review & editing. E. F.: formal analysis, investigation, methodology, visualization. A. J. M.-M.: formal analysis, investigation, methodology. F. N.: formal analysis, investigation, methodology. M. G.: funding acquisition, project administration, resources, supervision, writing – review & editing. J. H.: funding acquisition, project administration, resources, supervision. J. A.: funding acquisition, project administration, resources, supervision, writing – review & editing. U. P.: funding acquisition, project administration, resources, supervision, writing – original draft.

## Conflicts of interest

There are no conflicts to declare.

## Supplementary Material

SC-017-D5SC08074F-s001

SC-017-D5SC08074F-s002

## Data Availability

The datasets generated during and/or analyzed during the current study are available in the KU Leuven RDR repository, https://doi.org/10.48804/LXAEID. All data is available from the corresponding author upon reasonable request. CCDC 2431430–2431432 contain the supplementary crystallographic data for this paper.^69*a*–*c*^ Supplementary information (SI) is available. See DOI: https://doi.org/10.1039/d5sc08074f.
