# Supplementary material for: Heteroaryl iminothioindoxyl (HA-ITI) photoswitches via regioselective aza-Wittig synthesis: unifying red-shifted absorption, large E/Z band separation, and tunable thermal recovery
Source: Chem Sci. 2025 Dec 24;17(7):3658–68. doi: 10.1039/d5sc08074f (PMC12729054; doi:10.1039/d5sc08074f)
Supplement: SC-017-D5SC08074F-s001 [file SC-017-D5SC08074F-s001.pdf]

Supplementary Information (SI) for Chemical Science.
This journal is © The Royal Society of Chemistry 2025

Electronic Supplementary Information

Heteroaryl Iminothioindoxyl (HA-ITI) Photoswitches via Regioselective Aza-Wittig Synthesis: Unifying Red-Shifted Absorption, Large *E*/*Z* Band Separation, and Tunable Thermal Recovery

Jialei Chen-Wu,[a] Carlos Benítez-Martín,*[b,c] José A. González-Delgado,[a] Flip de Jong,[d] Eduard Fron,[d,e] Gert Steurs,[d,f] Antonio J. Martínez-Martínez,[a] Francisco Nájera,[g,h] Morten Grøtli,*[b] Johan Hofkens,*[d,i] Joakim Andréasson,*[c] Uwe Pischel*[a]

Affiliations

[a] *CIQSO – Center for Research in Sustainable Chemistry and Department of Chemistry, University of Huelva, Campus de El Carmen s/n, E-21071 Huelva, Spain*

[b] *Department of Chemistry and Molecular Biology, University of Gothenburg, 40530 Göteborg, Sweden*

[c] *Chemistry and Chemical Engineering, Chemistry and Biochemistry, Chalmers University of Technology, 41296 Göteborg, Sweden*

[d] *KU Leuven, Department of Chemistry, Celestijnenlaan 200F, B-3001 Leuven, Belgium*

[e] *KU Leuven, Core Facility for Advanced Spectroscopy, Celestijnenlaan 200F, B-3001 Leuven, Belgium*

[f] *KU Leuven, Core Facility for Liquid-State NMR Spectroscopy, Celestijnenlaan 200F, B-3001 Leuven, Belgium*

[g] *Departamento de Química Orgánica, Universidad de Málaga, Andalucía-Tech Campus Teatinos s/n, Málaga ES-29071, Spain*

[h] *Instituto de Investigación Biomédica de Málaga y Plataforma en Nanomedicina–IBIMA, Plataforma Bionand, Parque Tecnológico de Andalucía, Málaga ES-29590, Spain*

[i] *Max Planck Institute for Polymer Research, Ackermannweg 10, 55128 Mainz, Germany*

Table of Contents

1. Previous works	S3
2. Methods and materials	S4
3. Synthetic procedures	S7
4. Characterization by NMR spectroscopy	S9
5. Characterization by HRMS (ESI–QTOF)	S18
6. Photochemistry	S20
7. Low-temperature NMR irradiation	S26
8. Crystallographic data	S31
9. Theoretical calculations	S36
10. References	S52

1. Previous works

Table S1. Examples of previously reported **Het-HTI** and **ITI** photoswitches and their characteristics.

Photoswitch (Z form)	Solvent	λ_{max} (Z)/ nm	λ_{max} (E)/ nm	$\Delta\lambda$/ nm	type [$t_{1/2}$ ($E\rightarrow Z$)/ ms]	Reference
**HTI**						
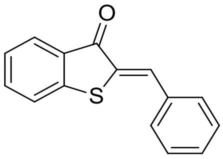	DCM	433	444	11	P	[1]
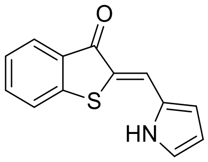	DCM	459	503	44	P	[1]
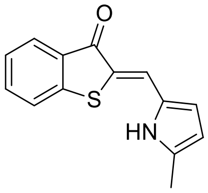	THF	471	516	45	P	[2]
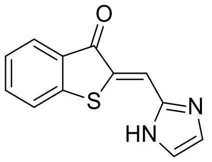	DCM	451	486	35	P	[1]
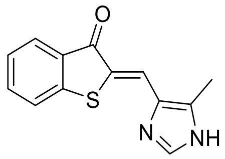	THF	452	492	40	P	[2]
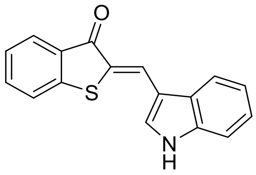	THF	455	487	32	P	[2]
**ITI**						
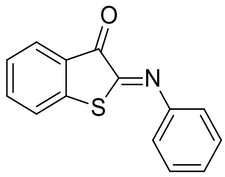	MeOH	429	515	86	T [18.5]	[3, 4]
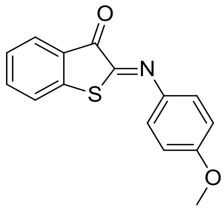	MeOH	448	516	68	T [5.3]	[3, 4]
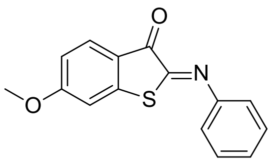	MeOH	460	540	80	T [20.0]	[4]

2. Methods and materials

Synthesis and characterization

All reagents and solvents for the synthesis were commercially available from Merck or BLDpharm and used as received without further purification. The products were purified by column chromatography on silica gel Merck-60 (230-400 mesh, 60 Å). Analytical TLC was performed on aluminum sheets pre-coated with silica gel 60 F_{254} (Merck). The ^{1}H, ^{13}C, and ^{31}P NMR measurements were done either on a Bruker Avance 400 MHz HPPR2 or a Bruker Avance 500 MHz HPPR2 instrument. Structural assignments were made with additional information from gCOSY and gHSQC experiments. The solvents for NMR measurements (CD_2Cl_2, $CDCl_3$, and THF-d_8; ≥ 99.6 atom% D) were purchased from Eurisotope. The residual solvent peak was used as the reference signal for the ^{1}H NMR spectra (δ = 3.58, 5.32, and 7.26 ppm for THF-d_8, CD_2Cl_2, and $CDCl_3$, respectively) and for the ^{13}C NMR spectra (δ = 53.84, 67.57, and 77.2 ppm for CD_2Cl_2, THF-d_8 and $CDCl_3$, respectively). For the ^{31}P NMR spectra, H_3PO_4 was used as an external reference and assigned a chemical shift of 0 ppm. High-resolution mass spectra were obtained on a Bruker Compact Elite QTOF with an electrospray ionization source (ESI) in positive mode.

Steady-state absorption spectroscopy

Room temperature (25 ºC) measurements were performed with air-equilibrated solutions held in quartz cuvettes with 10 mm optical pathlength. Acetonitrile (MeCN), 2-methyltetrahydrofuran (MTHF), dimethylsulfoxide (DMSO), toluene, methanol (MeOH) and dichloromethane (DCM), which were used for the spectroscopic measurements, were of the highest purity available from Merck (spectroscopic grade).
Low-temperature steady-state absorption measurements were conducted using a Janis VPF-100 cryostat, cooled with liquid nitrogen. The cryostat was fitted with a quartz cuvette of 1 mm optical pathlength, and MTHF was used as the solvent. Photoswitching experiments were carried out using a Coherent CUBE laser (375 nm), equipped with a diverging lens to maximize uniform excitation. Please note that the small deviations in the fittings from first-order kinetics are potentially associated with diffusion-related issues due to the use of low temperatures.

Transient absorption measurements

Transient absorption measurements from the nanosecond to millisecond timescale were performed using a 355 nm laser pulse (8 ns pulse duration, 10 Hz repetition rate) generated by a pulsed Nd:YAG laser system (Quanta-Ray INDI-40, Spectra Physics). The excitation beam was focused on the sample using a 300 mm focal length achromatic lens. A small portion of the excitation light was diverted to a fast photodiode to generate a trigger signal for synchronization. The probe light was produced by a xenon flash lamp system, comprising a pulse generator and light source from Müller Elektronik-Optik (Pulseinheit MSP05m, SVX 1450, LAX 1450). The probe beam was directed and focused using the same 300 mm focal length lens and spatially overlapped with the excitation beam on the sample in an approximately collinear geometry. After passing through the sample, the probe light was filtered and focused on the entrance slit of a 300 mm focal length monochromator (SpectroPro-300i), which was used to disperse the light and select the desired detection wavelength. The transmitted or emitted light was detected using a photomultiplier tube (PMT, Hamamatsu R928). The resulting transient electrical signal was amplified and recorded by a computer-controlled oscilloscope. Instrument control, synchronization, data acquisition, and signal averaging were managed using custom software developed in LabVIEW. All samples were contained in 10 mm pathlength quartz cuvettes. Measurements were conducted in a 2 ms time window over a 400–700 nm detection range. The decays were recorded with 10,000 channels, and analyzed individually and globally with Origin software. The prompt response (or instrumental response function, IRF) of this setup, was estimated to be about 10 ns, determined primarily by the laser pulse duration and jitter.

Low-temperature irradiation and observation by NMR spectroscopy

High-field nuclear magnetic resonance (NMR) spectra were recorded on a Bruker Avance Neo 600 spectrometer with an Ascend^{TM} 600 magnet system and a 5 mm PI HR-TBO (BB/F-H/F-D) probe with z-gradients. All samples were dissolved in tetrahydrofuran-d_8. Probe temperature regulation was achieved using a Bruker LN2 heat exchanger, and the sample temperature was calibrated using a reference sample of 4% methanol in methanol-d_4. Data were recorded using Bruker TopSpin 4.4.1 GxP and processed and analyzed using Bruker TopSpin 4.4.1. ^{1}H and ^{13}C data were calibrated using tetramethylsilane (TMS) as an internal calibration reference. Further data processing and statistical analysis were carried out using GraphPad Prism 10.4.1.
In situ laser irradiation of the NMR samples inside the NMR magnet was achieved using a custom-built in-house setup. For this purpose, a 200 mW, PWM-controlled laser (450 nm) was coupled to a 1 mm ID fiber-optic cable. At the end of the cable, around 20 cm of the cladding was stripped off and the last 4.2 cm of the fiber were sanded with 240 grit sandpaper. The stripped end of the cable was then encapsulated in a glass capillary to protect the cable from the sample solvent. The glass capillary was then inserted into the NMR sample (see Figure S1).

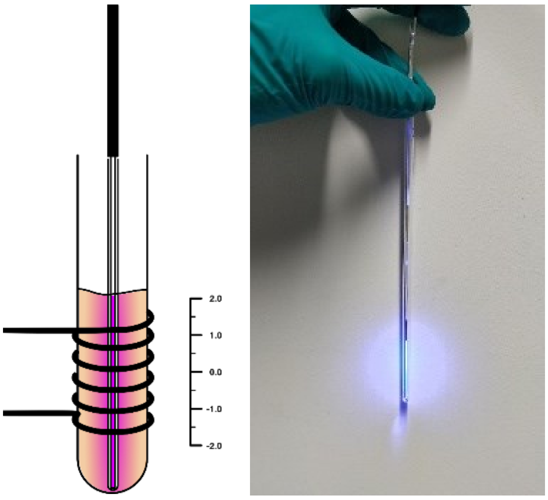

Figure S1. In situ NMR irradiation experiment.

Single-crystal X-ray diffraction

Single-crystal X-ray diffraction data for **6**, **7**, and **8** were collected (ω-scans) on a Rigaku Synergy-S diffractometer with Cu-*Kα* radiation (λ = 1.54184 Å) equipped with a nitrogen gas Oxford Cryosystems Cryostream 1000 unit.[5] Diffraction data were reduced and processed using CrysAlisPro package.[6] The structures were solved using SHELXT[7] and refined to convergence on F^2 and against all independent reflections by full-matrix least-squares using SHELXL[8] in combination with the GUI OLEX2[9] program. All non-hydrogen atoms were refined anisotropically, and hydrogen atoms were geometrically placed unless otherwise stated (see specific details for each molecular structure in the text) and allowed to ride on their parent atoms. Distances and angles were calculated using the full covariance matrix. Selected crystallographic data are summarized in the text and full details are given in the supplementary deposited CIF files (CCDC 2431430-2431432). These data can be obtained free of charge from the Cambridge Crystallographic Data Centre via http://optimized.ccdc.cam.ac.uk/data_request/cif.

Theoretical calculations

The Gaussian 16C01 package was employed for calculations. The geometrical parameters for the ground state (S_0) were determined with the density functional level of theory (DFT), employing the M06-2X

functional[10, 11] and the 6-311+G(d,p) basis set. Solvent effects were considered by applying the solvation model based on density (SMD).[12] Custom parameters were employed for 2-methyltetrahydrofuran (see below), while default parameters were used for acetonitrile and tetrahydrofuran. The absolute nature of the energetic minima was confirmed by the absence of any negative frequencies in the vibrational analysis. The transition states (TS) were determined at the same level of theory and were characterized by the presence of a single negative frequency. An intrinsic reaction coordinate (IRC) calculation yielded the two minima connected through the optimized TS. Energy parameters were calculated as vertical electronic excitations from the S_0 minimum structure using the linear response (LR) approach and the time-dependent density functional theory (TDDFT). These calculations were carried out for the first fifteen excited states using the mPW1PW91 functional[13] and the 6-311+G(d,p) basis set on previously optimized structures.

Parameters used for 2-methyltetrahydrofuran:

- Eps = 6.97
- EpsInf = 1.9776
- HbondAcidity = 0.00
- HbondBasicity = 0.46
- SurfaceTensionAtInterface = 25.0
- CarbonAromaticity = 0.00
- ElectronegativeHalogenicity = 0.72

3. Synthetic procedures

The phosphanimine **3** was synthesized from commercially available *N*-methylindole (**1**), according to a published route.[14] Likewise, the phosphanimine **4** was prepared by following a reported protocol.[15]

N-(1-methyl-1H-benzo[d]imidazol-2-yl)-1,1,1-triphenylphosphanimine (5)

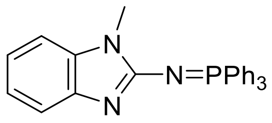

5

An oven-dried 100 mL Schlenk tube was charged with 1-methyl-1*H*-benzo[*d*]imidazol-2-amine (0.5 g, 3.40 mmol), TEA (1.03 g, 10.3 mmol) and dry toluene (10 mL) under a nitrogen atmosphere. Subsequently, a solution of triphenylphosphine dibromide (1.43 g, 3.4 mmol) in dry toluene was added, the reaction mixture was stirred at 90 ºC overnight. The solvent was removed under reduced pressure, and the resulting solid was re-dissolved in dichloromethane. The organic phase was washed with water and subsequently dried over anhydrous Na_2SO_4. After filtration, the organic solvent was evaporated under reduced pressure, and the crude product was purified by column chromatography on silica gel (Hex/EtOAc 4:1) to yield **5** (1.10 g, 80% yield) as a colorless solid.

^{1}H NMR (400 MHz, CD_2Cl_2): *δ* 7.93 – 7.80 (m, 6H), 7.63 – 7.55 (m, 3H), 7.53 – 7.45 (m, 6H), 7.18 – 7.12 (m, 1H), 7.03 – 6.98 (m, 1H), 6.96 – 6.89 (m, 2H), 3.71 (s, 3H).

^{13}C{^{1}H} NMR (101 MHz, CD_2Cl_2): *δ* 158.1, 143.3, 135.9, 133.5 (3C), 133.4 (3C), 132.6 (2C), 132.5 (2C), 130.3, 129.3, 129.0 (3C), 128.9 (3C), 120.2, 118.4, 115.3, 107.0, 28.8.

^{31}P{^{1}H} NMR (162 MHz, CD_2Cl_2): *δ* 14.83.

HRMS (ESI, QTOF): *m/z*: found $[M+H]^+$ 408.1600; molecular formula $C_{26}H_{23}N_3P$ requires 408.1624.

(Z)-2-((1-methyl-1H-indol-2-yl)imino)benzo[b]thiophen-3(2H)-one (6)

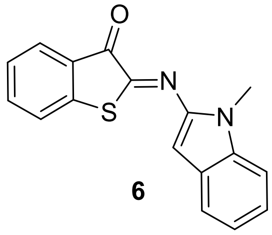

6

An oven-dried 100 mL Schlenk tube was charged with **3** (0.40 g, 1 mmol), benzo[*b*]thiophene-2,3-dione (0.16 g, 1 mmol) and dry toluene (15 mL) under nitrogen atmosphere. Subsequently, the reaction mixture was stirred at 90 ºC overnight. The solvent was then evaporated under reduced pressure and the crude product was purified by column chromatography on silica gel (Hex/EtOAc 1:1) to afford **6** (0.04 g, 15% yield) as an orange powder.

^{1}H NMR (500 MHz, $CDCl_3$): *δ* 7.95 (d, *J* = 7.6 Hz, 1H), 7.70 (d, *J* = 7.9 Hz, 1H), 7.64 (td, *J* = 7.6, 1.4 Hz, 1H), 7.50 (d, *J* = 7.8 Hz, 1H), 7.38 – 7.35 (m, 2H), 7.32 (ddd, *J* = 8.2, 6.8, 1.1 Hz, 1H), 7.15 (ddd, *J* = 8.1, 6.9, 1.2 Hz, 1H), 6.81 (s, 1H), 3.94 (s, 3H).

^{13}C{^{1}H} NMR (126 MHz, $CDCl_3$): *δ* 186.0, 150.8, 143.3, 142.2, 137.9, 136.6, 128.4, 127.7, 127.6, 127.1, 125.0, 124.8, 122.4, 120.9, 110.1, 96.8, 29.1.

HRMS (ESI, QTOF): *m/z*: found $[M+Na]^+$ 315.0535; molecular formula $C_{17}H_{12}N_2OSNa$ requires 315.0563.

(Z)-2-((1H-benzo[d]imidazol-2-yl)imino)benzo[b]thiophen-3(2H)-one (7)

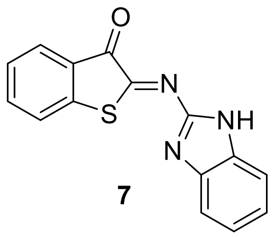

7

An oven-dried 100 mL Schlenk tube was charged with **4** (0.2 g, 0.5 mmol), benzo[*b*]thiophene-2,3-dione (0.08 g, 0.5 mmol) and dry toluene (10 mL) under nitrogen atmosphere. Subsequently, the reaction mixture was stirred at 90 °C overnight. The resulting suspension was filtered, and the solid was washed several times with DCM and dried *in vacuo* to afford **7** (0.09 g, 65% yield) as an orange powder.

^{1}H NMR (500 MHz, $CDCl_3$): *δ* 9.68 (s, 1H), 7.96 (dd, *J* = 7.6, 1.7 Hz, 1H), 7.92 – 7.87 (m, 1H), 7.67 (td, *J* = 7.6, 1.4 Hz, 1H), 7.56 – 7.49 (m, 2H), 7.41 – 7.31 (m, 3H).

^{13}C{^{1}H} NMR (126 MHz, $CDCl_3$): *δ* 187.2, 160.7, 152.3, 146.3, 143.1, 137.5, 133.7, 127.7, 127.4, 127.3, 125.1, 125.0, 123.6, 121.0, 111.6.

HRMS (ESI, QTOF): *m/z*: found $[M+Na]^+$ 302.0339; molecular formula $C_{15}H_9N_3OSNa$ requires 302.0359.

(Z)-2-((1-methyl-1H-benzo[d]imidazol-2-yl)imino)benzo[b]thiophen-3(2H)-one (8)

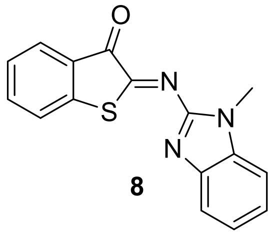

8

An oven-dried 100 mL Schlenk tube was charged with **5** (0.2 g, 0.5 mmol), benzo[*b*]thiophene-2,3-dione (0.08 g, 0.5 mmol) and dry toluene (10 mL) under nitrogen atmosphere. Subsequently, the reaction mixture was stirred at 90 °C overnight. The resulting suspension was filtered, and the solid was washed several times with DCM and dried *in vacuo* to afford **8** (0.08 g, 55% yield) as an orange powder.

^{1}H NMR (400 MHz, CD_2Cl_2): *δ* 7.89 (d, *J* = 7.0 Hz, 1H), 7.81 (d, *J* = 8.1 Hz, 1H), 7.67 (t, *J* = 7.6 Hz, 1H), 7.54 (d, *J* = 7.7 Hz, 1H), 7.46 (d, *J* = 7.4 Hz, 1H), 7.40 – 7.31 (m, 3H), 3.99 (s, 3H).

^{13}C{^{1}H} NMR (126 MHz, CD_2Cl_2): *δ* 186.9, 160.3, 152.7, 146.5, 142.5, 137.4, 136.3, 127.9, 127.41, 127.4, 125.4, 124.6, 123.6, 120.7, 110.5, 29.6.

HRMS (ESI, QTOF): *m/z*: found $[M+Na]^+$ 316.0497; molecular formula $C_{16}H_{11}N_3OSNa$ requires 316.0515.

4. Characterization by NMR spectroscopy

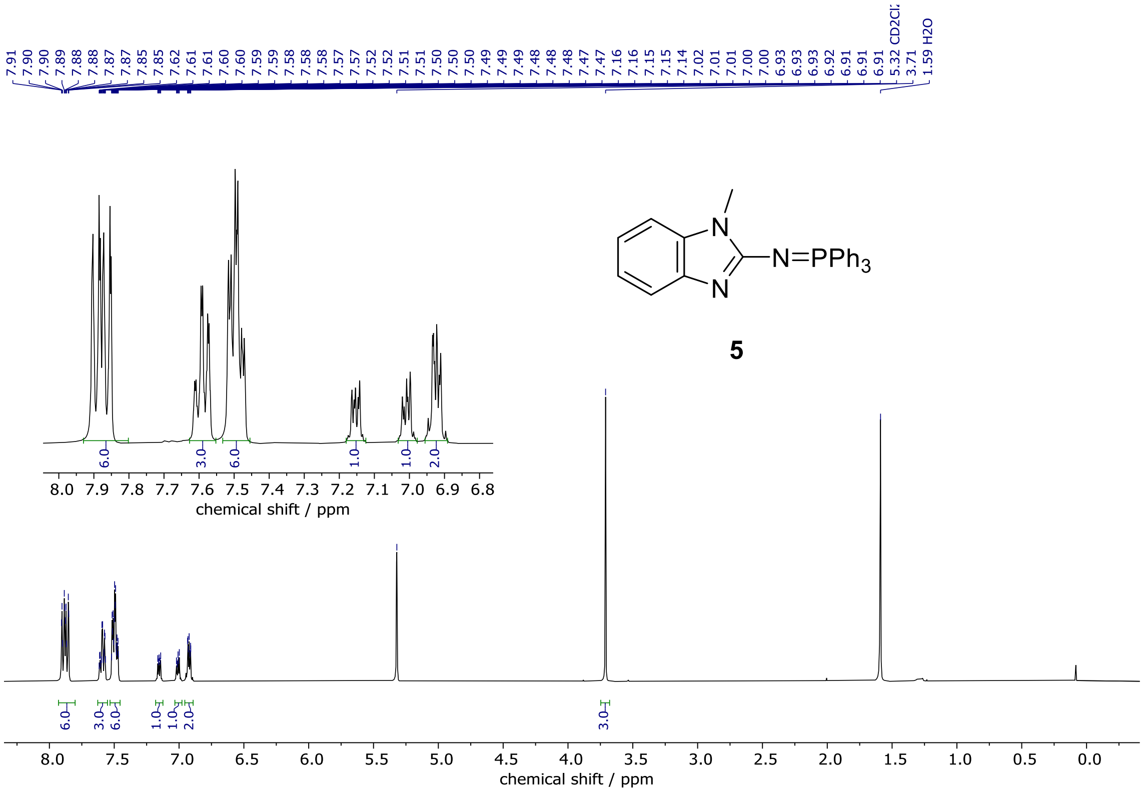

Figure S2. ^{1}H NMR spectrum (400 MHz) of **5** in CD_2Cl_2 at 298 K. The inset shows the aromatic signal region between 8.00 and 6.80 ppm.

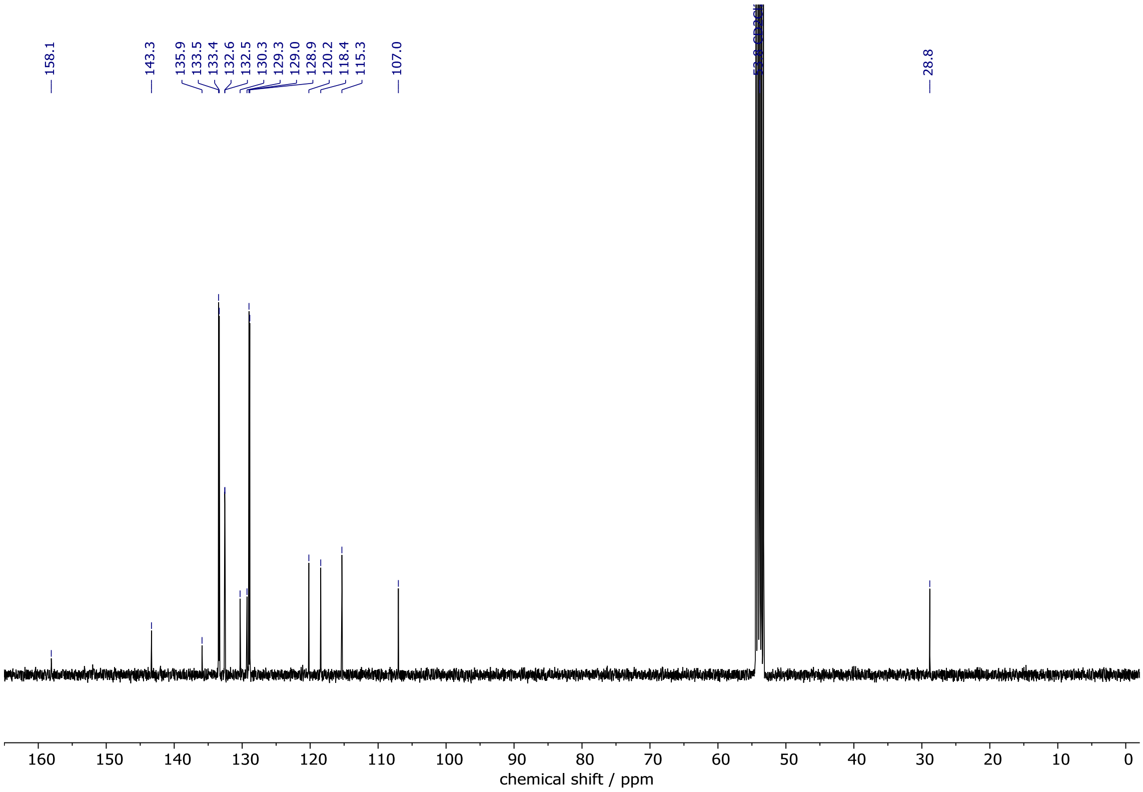

Figure S3. $^{13}C\{^{1}H\}$ NMR spectrum (101 MHz) of **5** in CD_2Cl_2 at 298 K.

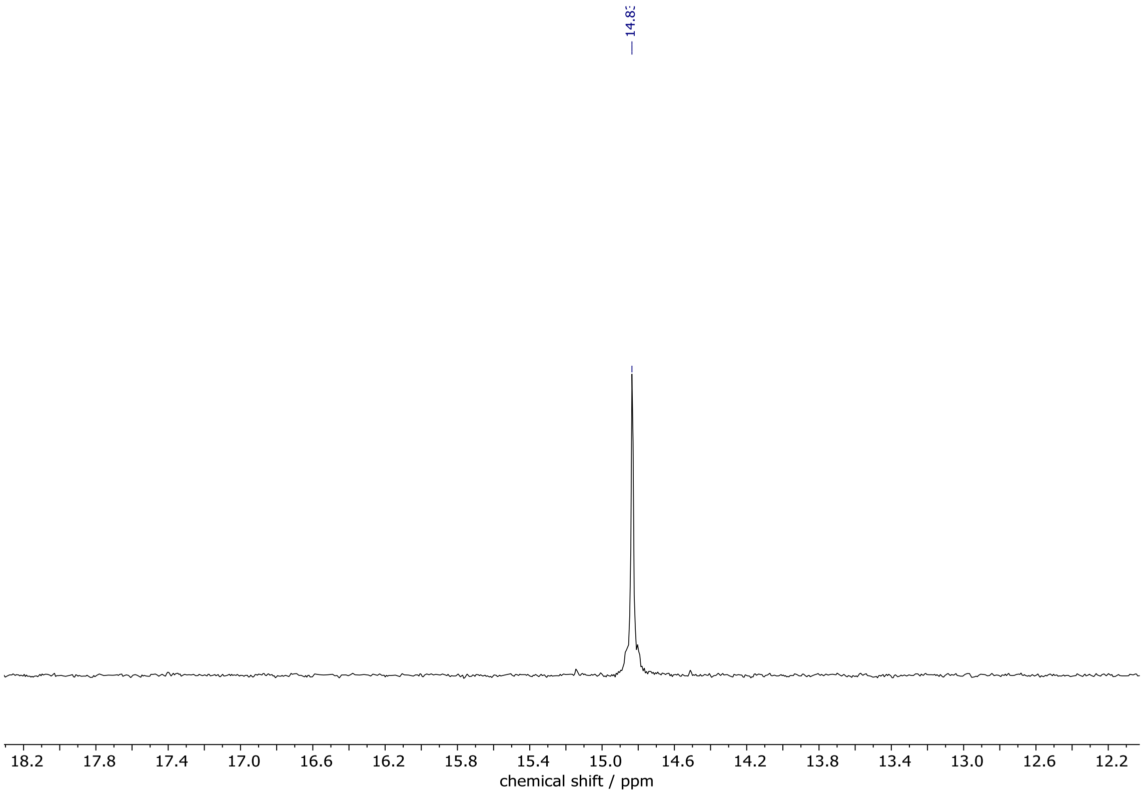

Figure S4. $^{31}P\{^{1}H\}$ NMR spectrum (162 MHz) of **5** in CD_2Cl_2 at 298 K.

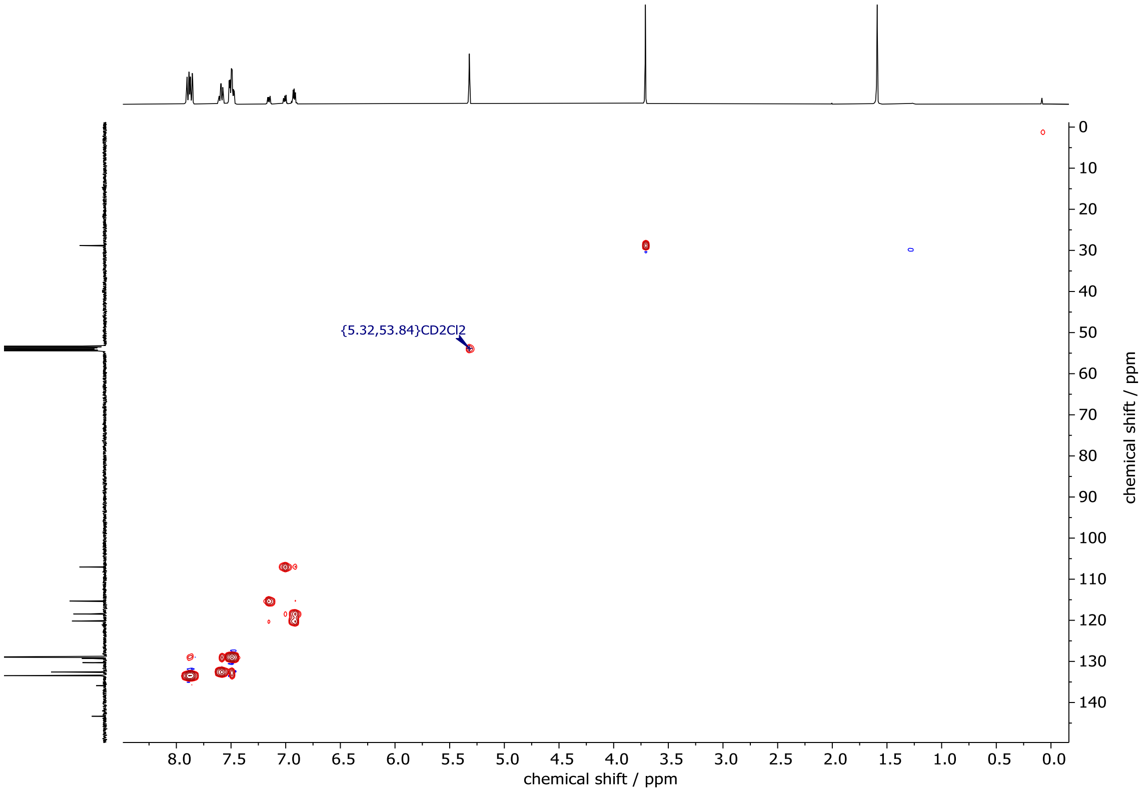

Figure S5. ^{1}H–^{13}C HSQC spectrum (400 MHz) of **5** in CD_2Cl_2 at 298 K.

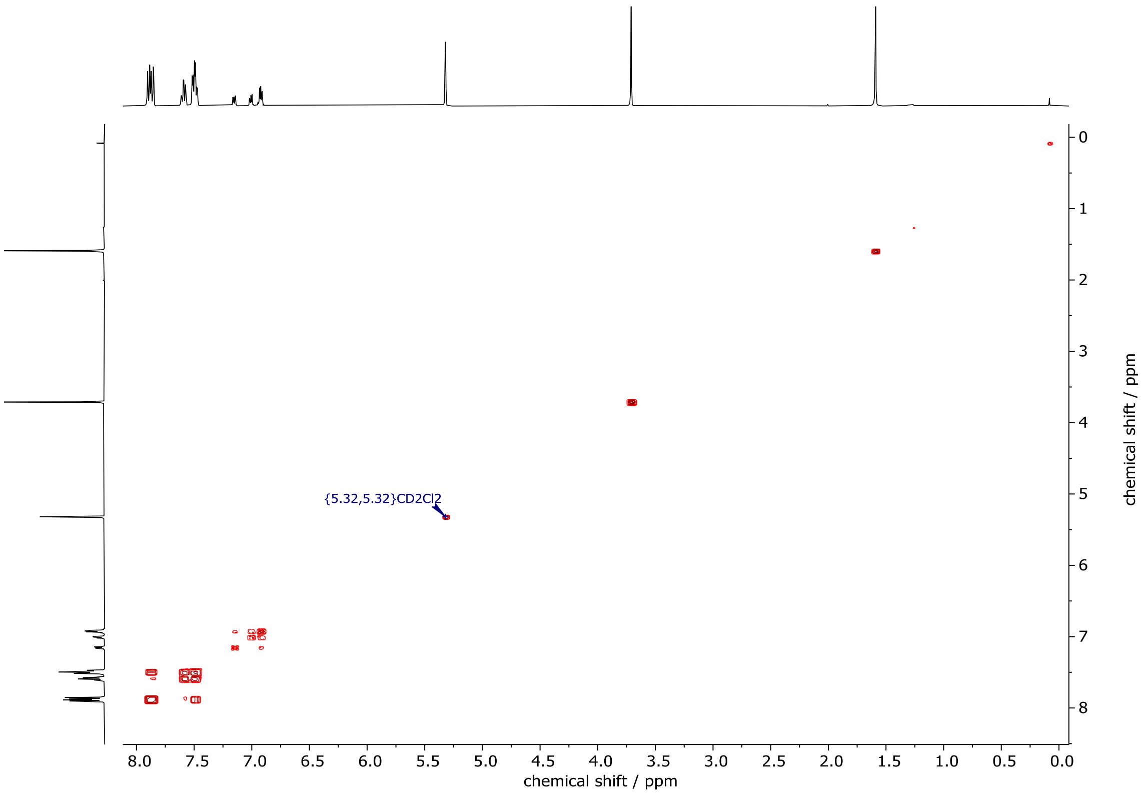

Figure S6. $^{1}H-^{1}H$ COSY spectrum (400 MHz) of **5** in CD_2Cl_2 at 298 K.

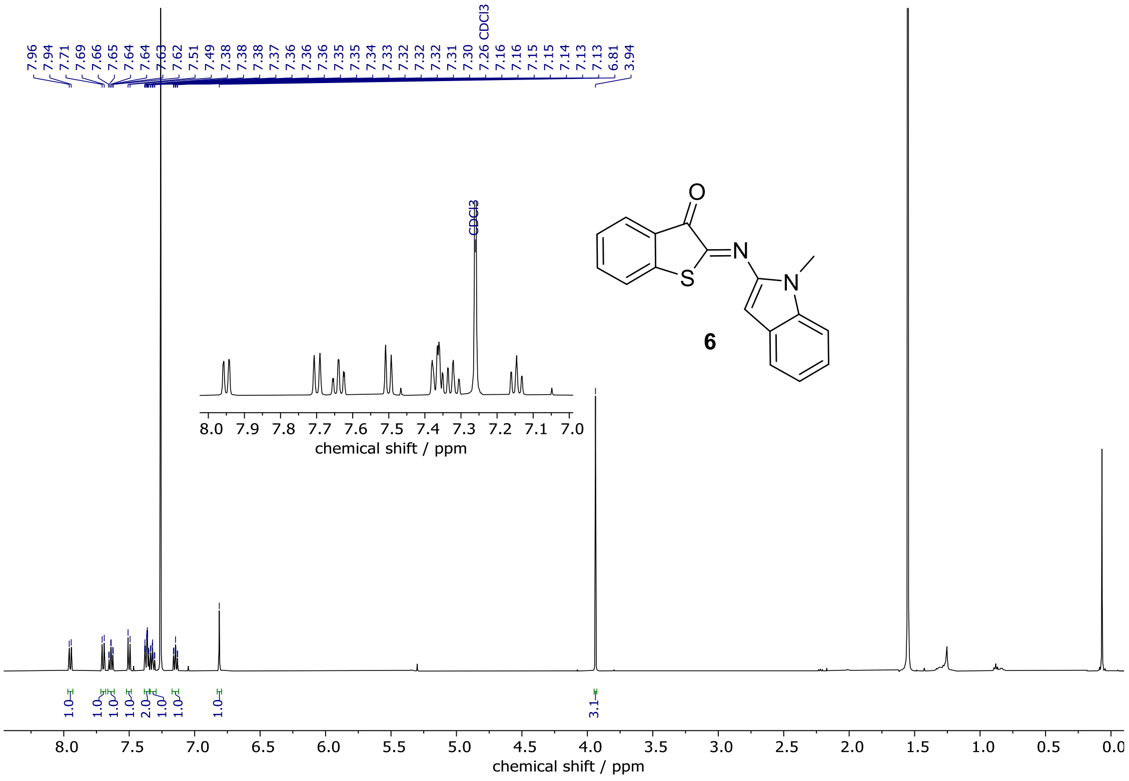

Figure S7. ^{1}H NMR spectrum (500 MHz) of **6** in $CDCl_3$ at 298 K. The inset shows the aromatic signal region between 8.00 and 7.00 ppm.

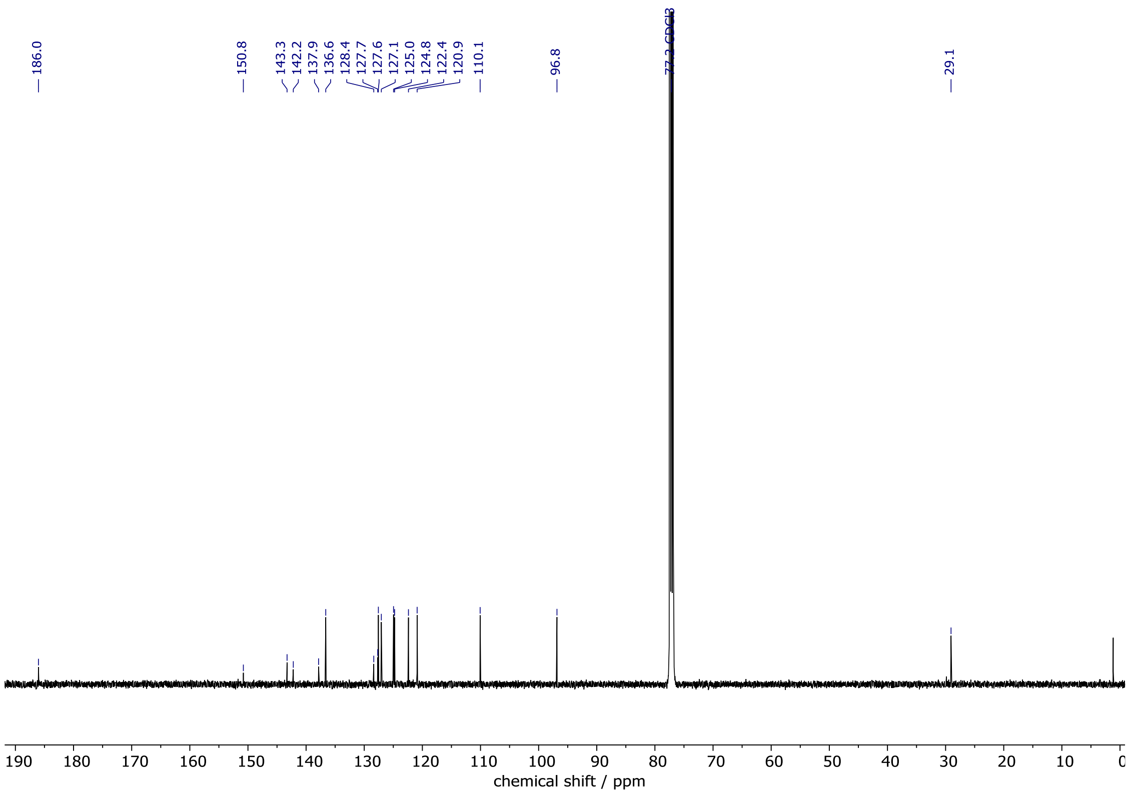

Figure S8. $^{13}C\{^{1}H\}$ NMR spectrum (126 MHz) of **6** in $CDCl_3$ at 298 K.

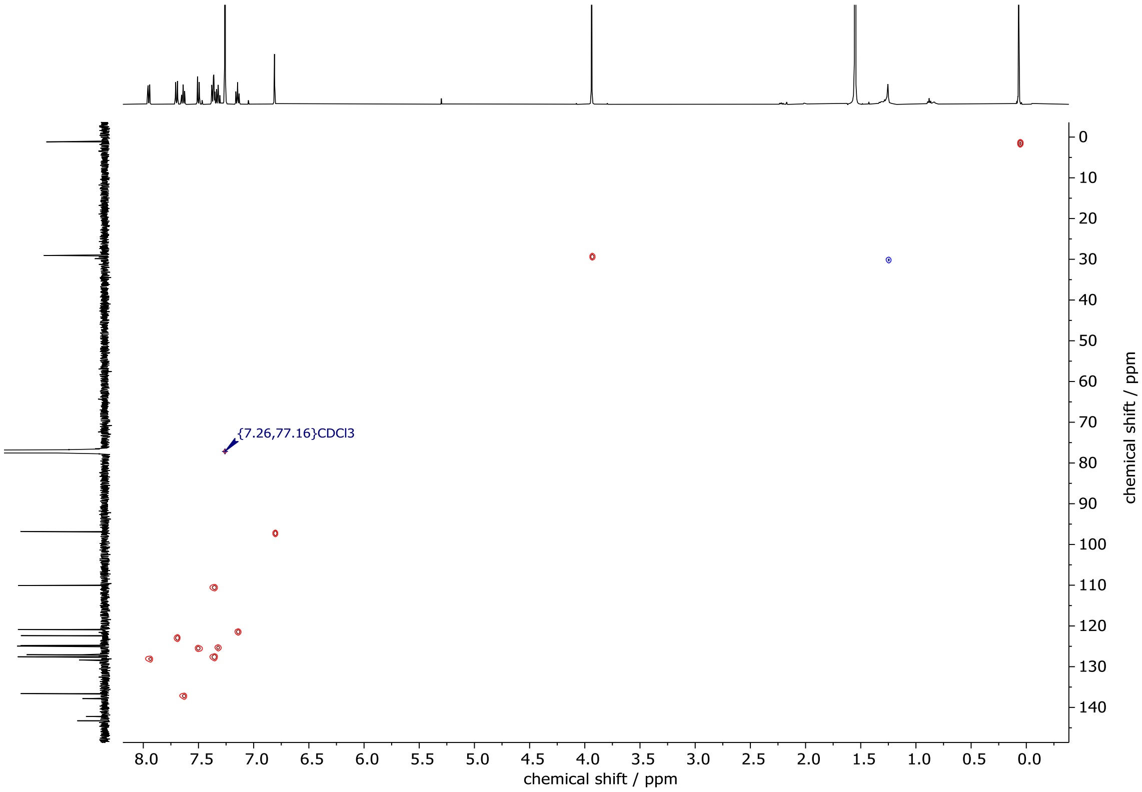

Figure S9. ^{1}H–^{13}C HSQC spectrum (500 MHz) of **6** in $CDCl_3$ at 298 K.

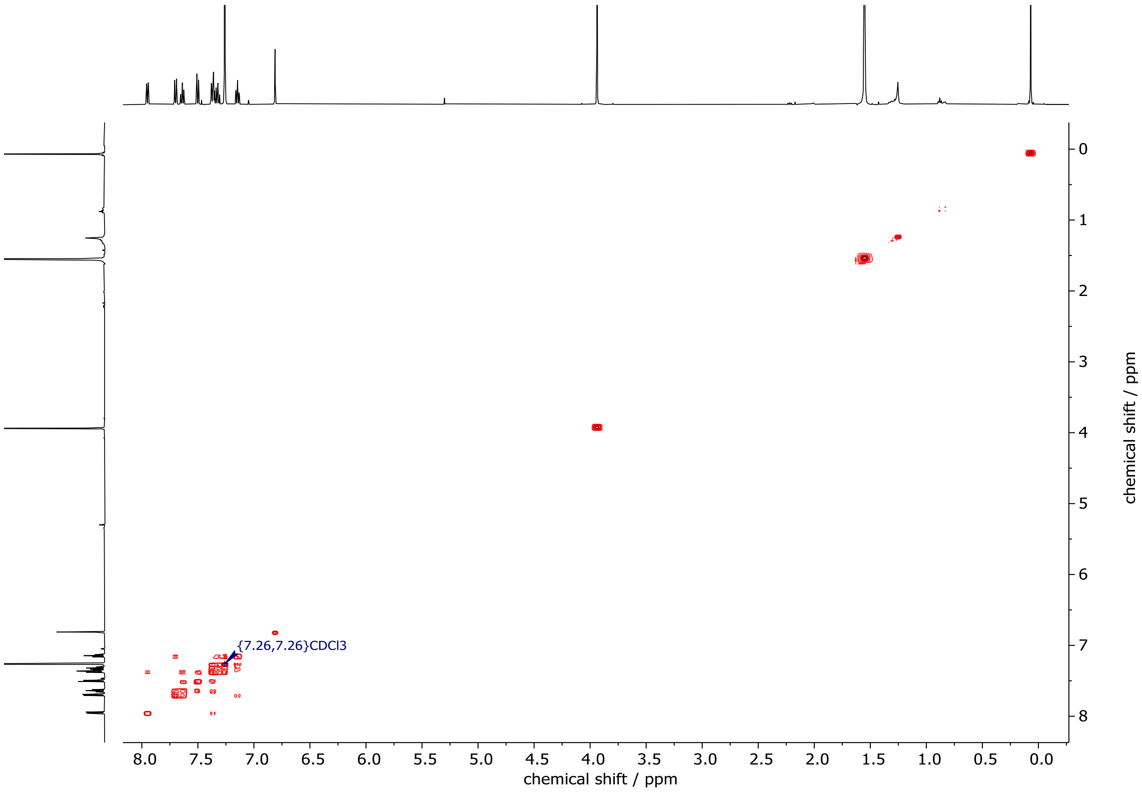

Figure S10. ^1H–^1H COSY spectrum (500 MHz) of **6** in $CDCl_3$ at 298 K.

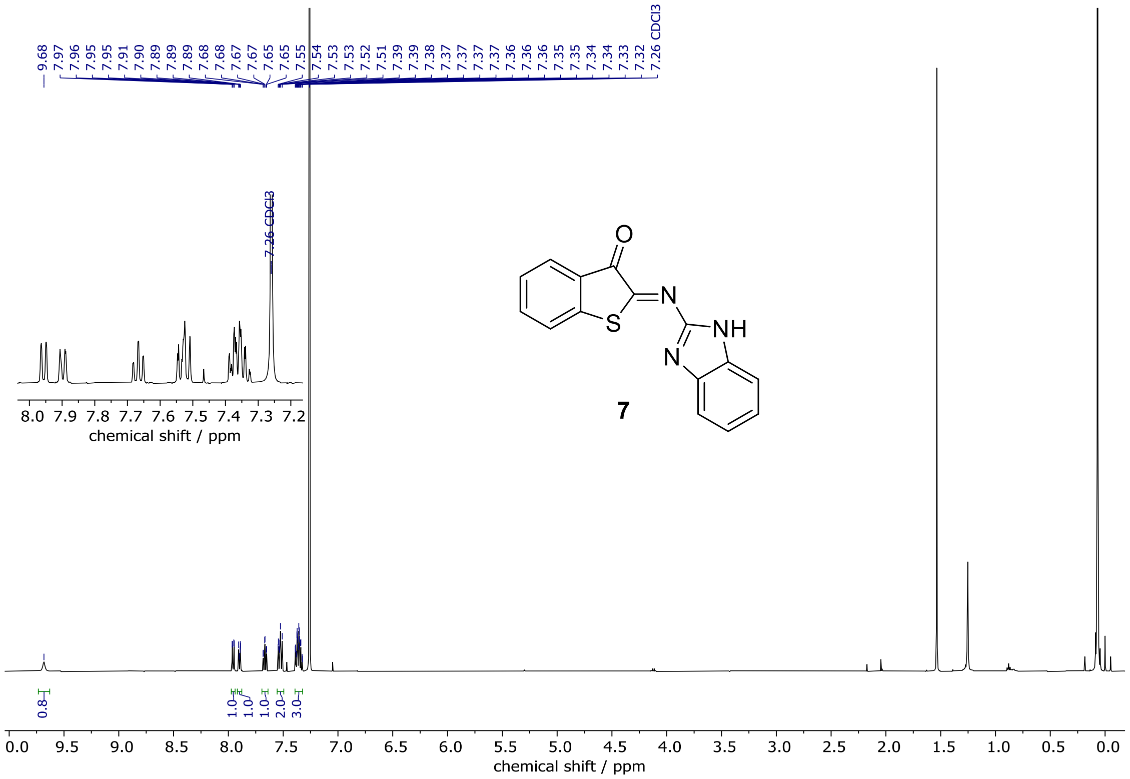

Figure S11. ^1H NMR spectrum (500 MHz) of **7** in $CDCl_3$ at 298 K. The inset shows the aromatic signal region between 8.00 and 7.20 ppm.

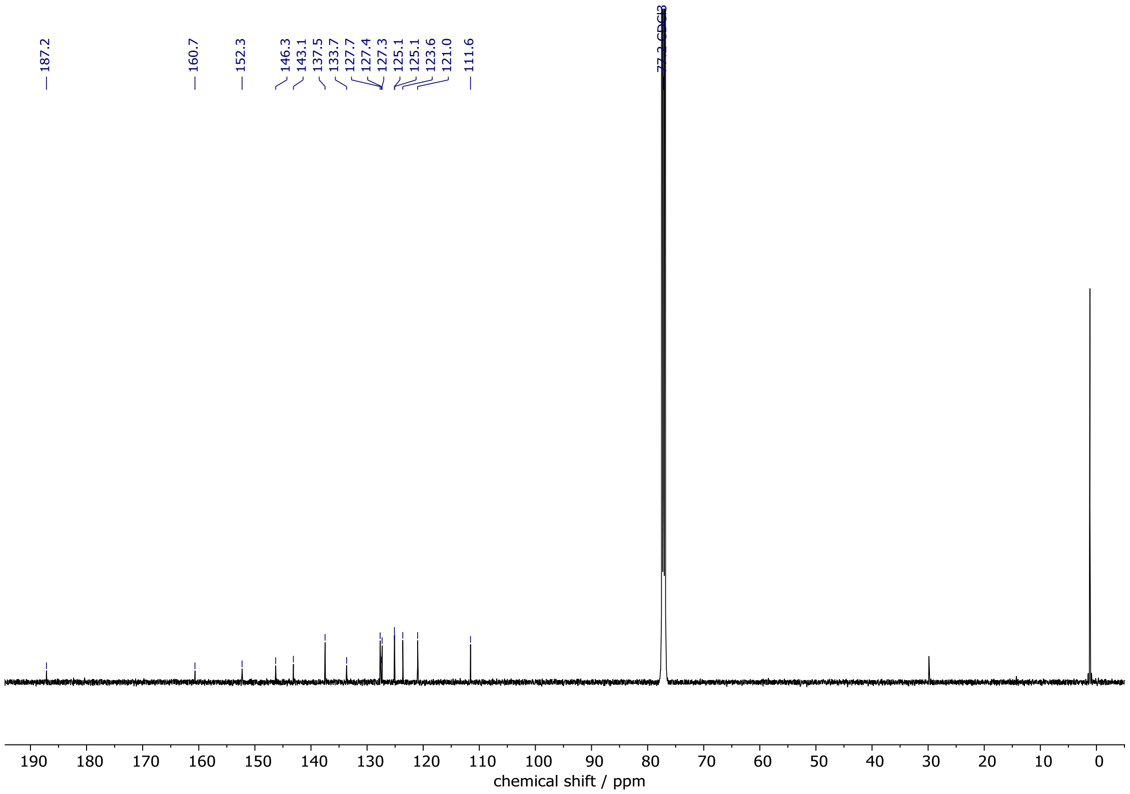

Figure S12. $^{13}C\{^{1}H\}$ NMR spectrum (126 MHz) of **7** in $CDCl_3$ at 298 K.

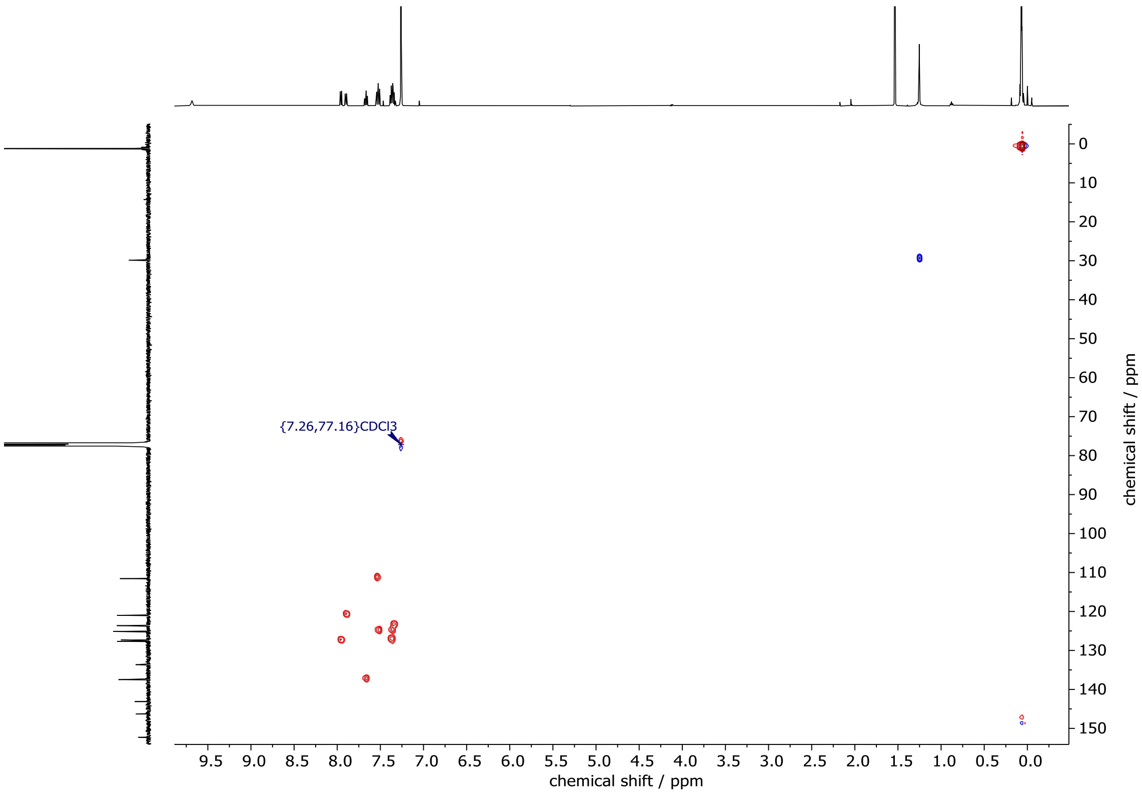

Figure S13. ^{1}H–^{13}C HSQC spectrum (500 MHz) of **7** in $CDCl_3$ at 298 K.

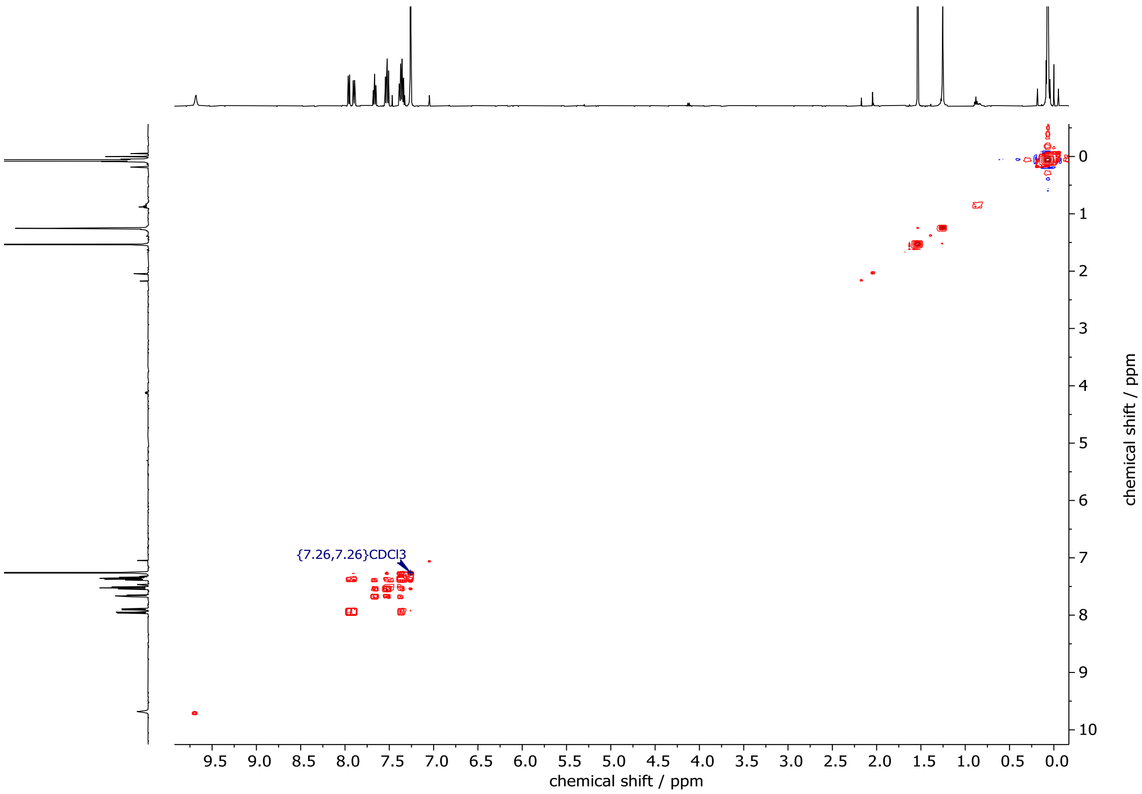

Figure S14. $^{1}H-^{1}H$ COSY spectrum (500 MHz) of **7** in $CDCl_3$ at 298 K.

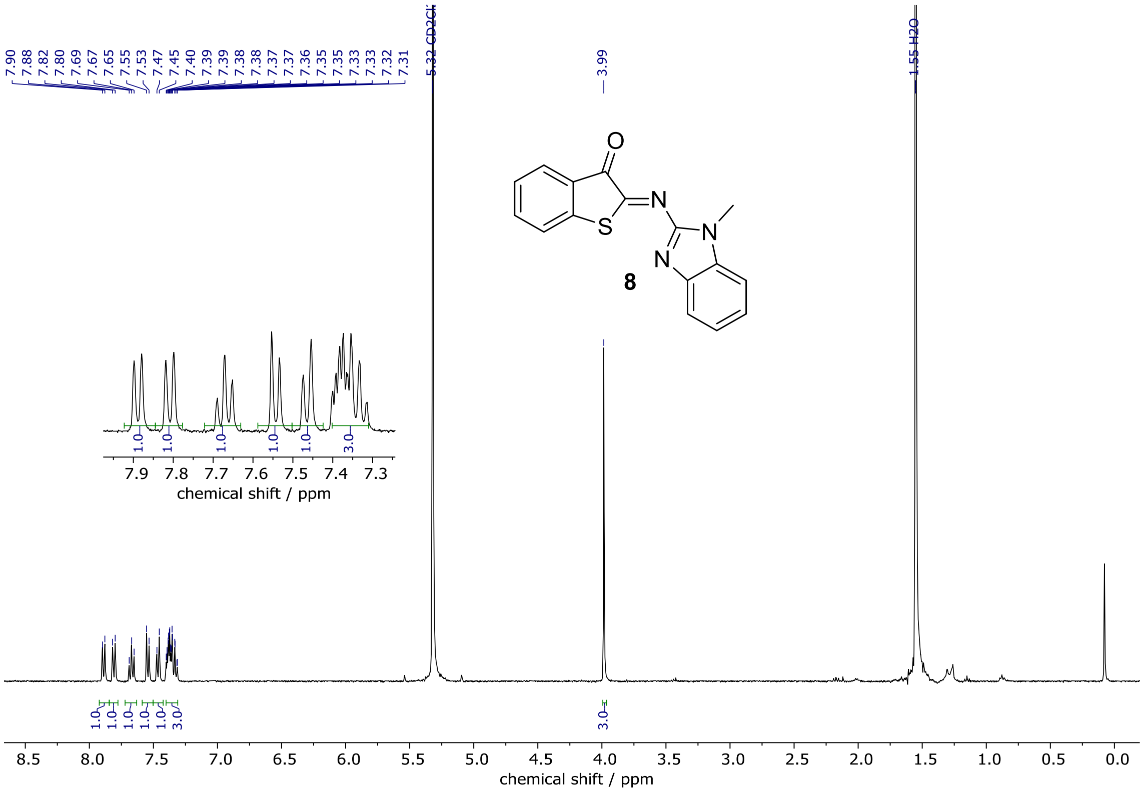

Figure S15. ^{1}H NMR spectrum (400 MHz) of **8** in CD_2Cl_2 at 298 K. The inset shows the aromatic signal region between 8.00 and 7.30 ppm.

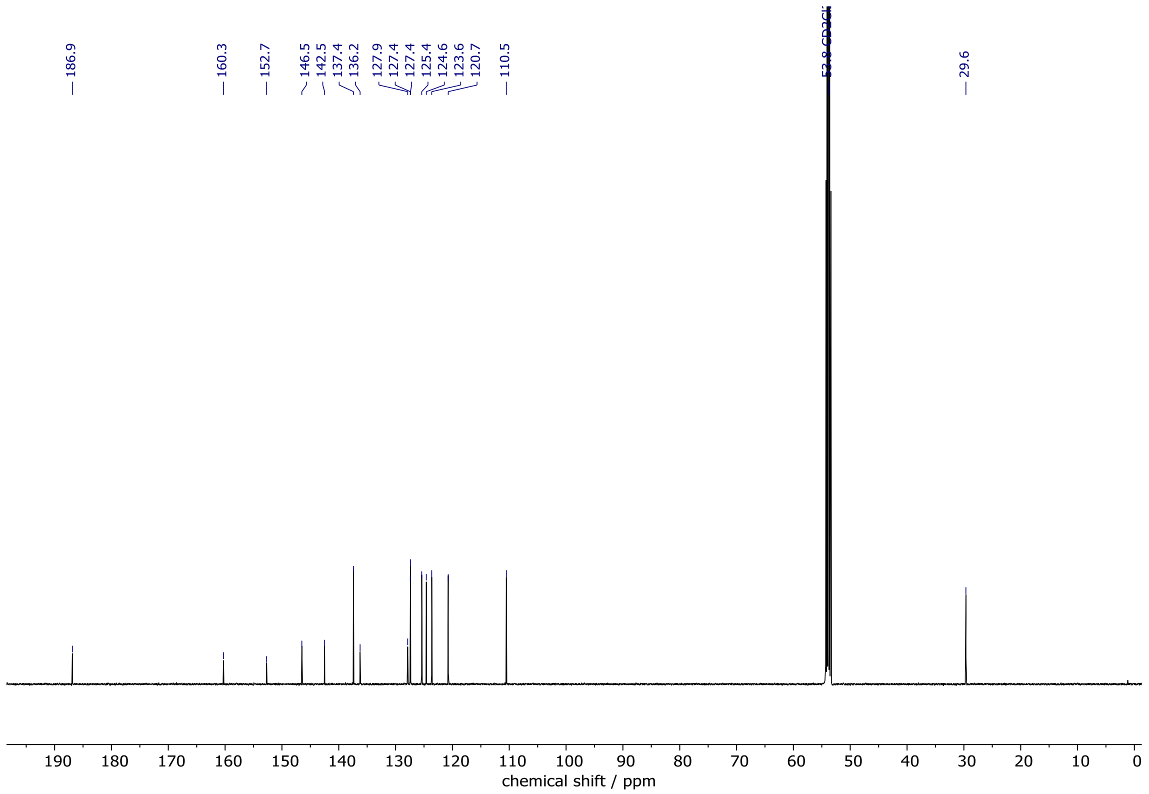

Figure S16. $^{13}C\{^1H\}$ NMR spectrum (126 MHz) of **8** in CD_2Cl_2 at 298 K

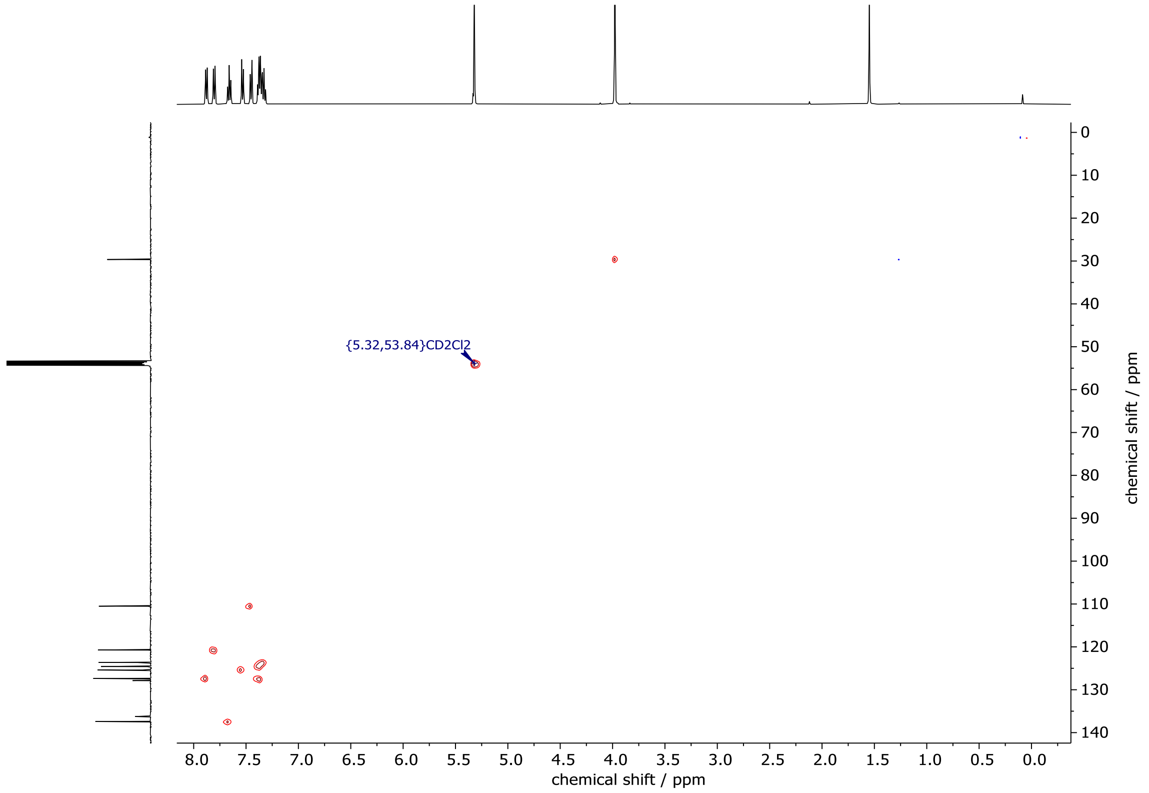

Figure S17. ^1H–^{13}C HSQC spectrum (500 MHz) of **8** in CD_2Cl_2 at 298 K.

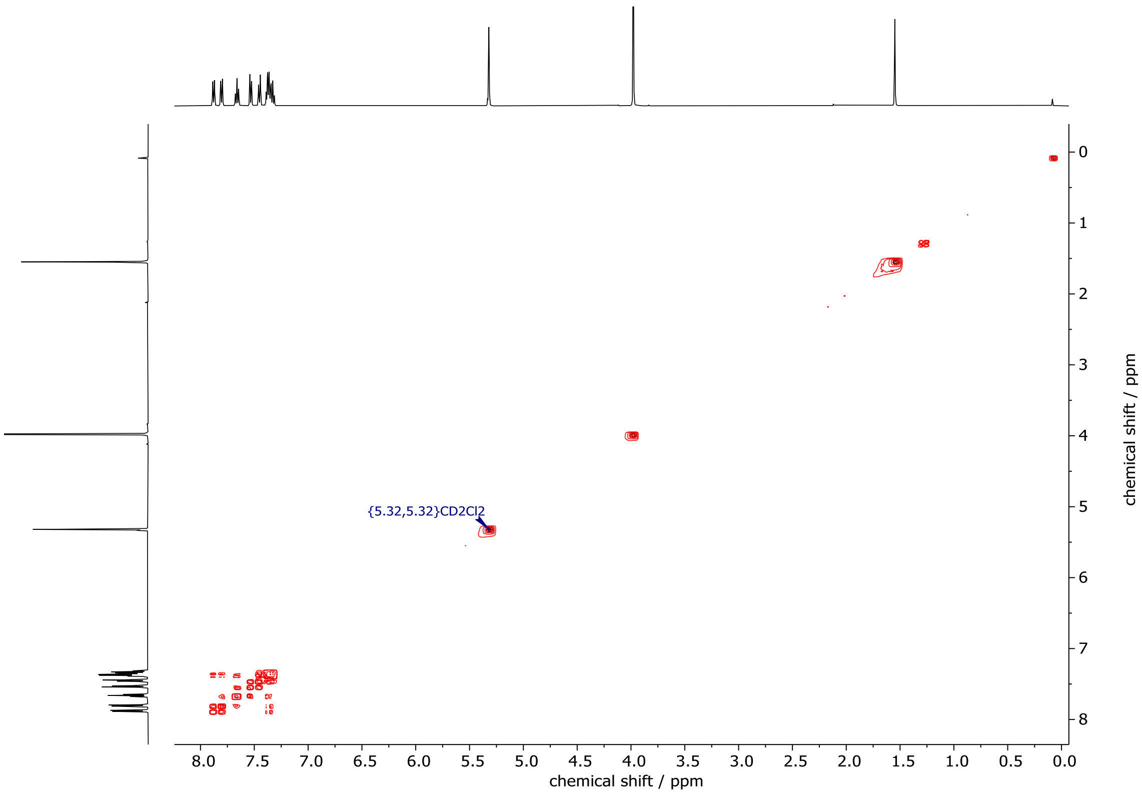

Figure S18. ^{1}H–^{1}H COSY spectrum (500 MHz) of **8** in CD_2Cl_2 at 298 K.

5. Characterization by HRMS (ESI–QTOF)

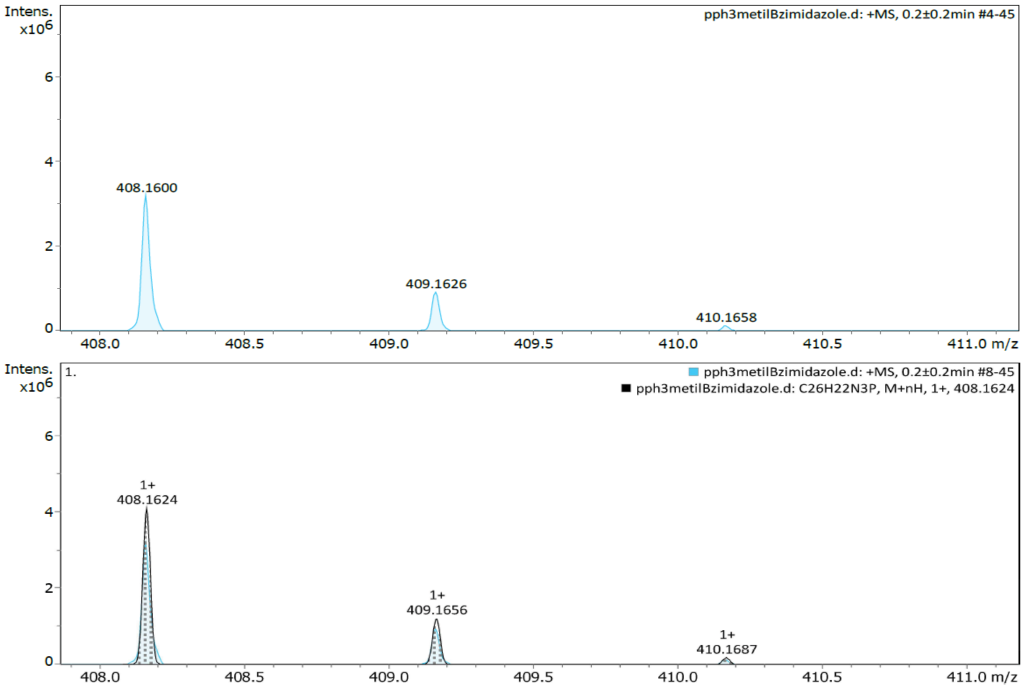

Figure S19. Top: HRMS (QTOF) spectrum of **5** in MeOH. Bottom: Calculated isotopic pattern (overlapped in black). Calculated for $[M+H]^+$; $C_{26}H_{23}N_3P$ 408.1624; found 408.1600.

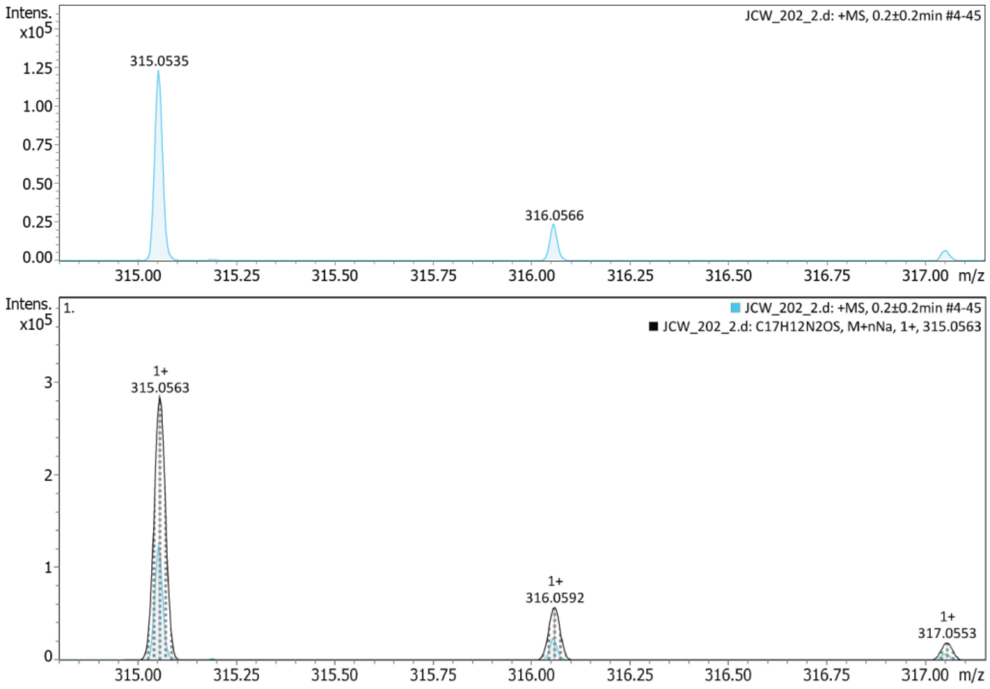

Figure S20. Top: HRMS (QTOF) spectrum of **6** in MeOH. Bottom: Calculated isotopic pattern (overlapped in black). Calculated for $[M+Na]^+$; $C_{17}H_{12}N_2OSNa$ 315.0563; found 315.0535.

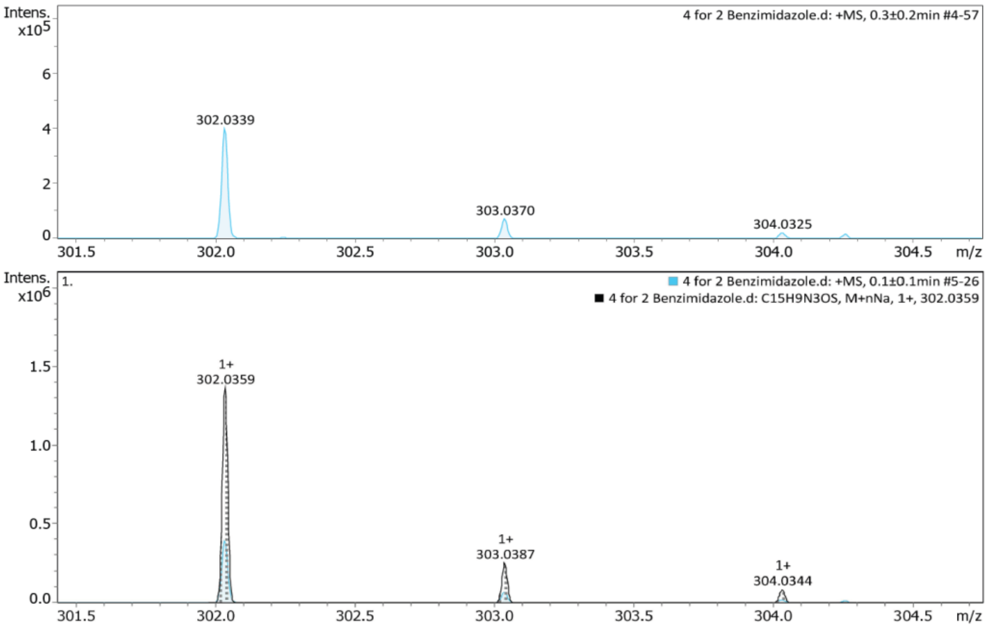

Figure S21. Top: HRMS (QTOF) spectrum of **7** in MeOH. Bottom: Calculated isotopic pattern (overlapped in black). Calculated for $[M+Na]^+$; $C_{15}H_9N_3OSNa$ 302.0359; found 302.0339.

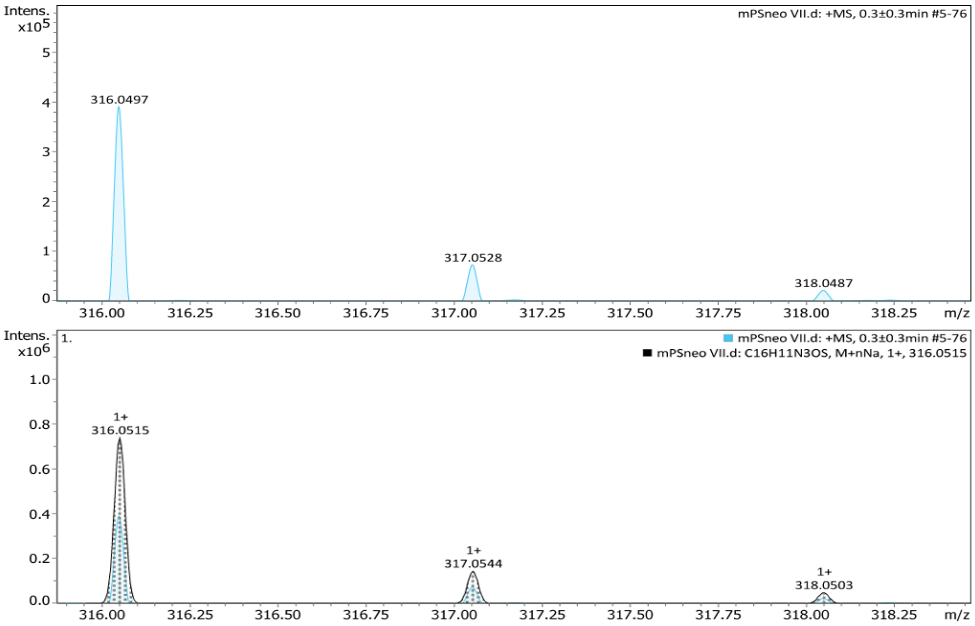

Figure S22. Top: HRMS (QTOF) spectrum of **8** in MeOH. Bottom: Calculated isotopic pattern (overlapped in black). Calculated for $[M+Na]^+$; $C_{16}H_{11}N_3OSNa$ 316.0515; found 316.0497.

6. Photochemistry

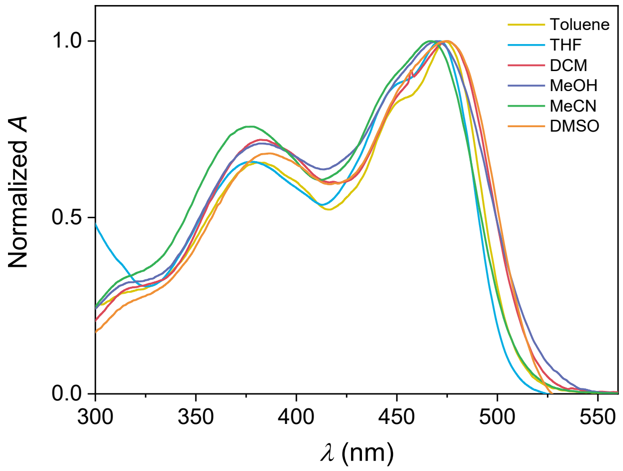

Solvent	λ_{abs} (nm) *Z*
Toluene	474
THF	471
DCM	475
MeOH	470
MeCN	467
DMSO	475

Figure S23. Normalized absorption spectra of *Z*-**7** in various solvents. Absorption maxima are indicated on the table at the right.

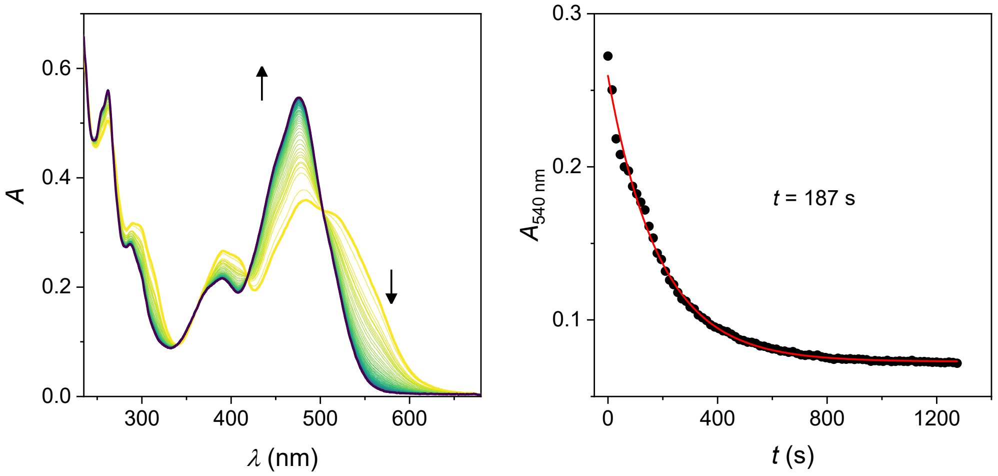

Figure S24. Thermal *E*→*Z* back isomerization (at 175 K) of **6** in MTHF after irradiation at 375 nm. *Left*: evolution of the UV/Vis absorption spectra. *Right*: kinetic trace at 540 nm.

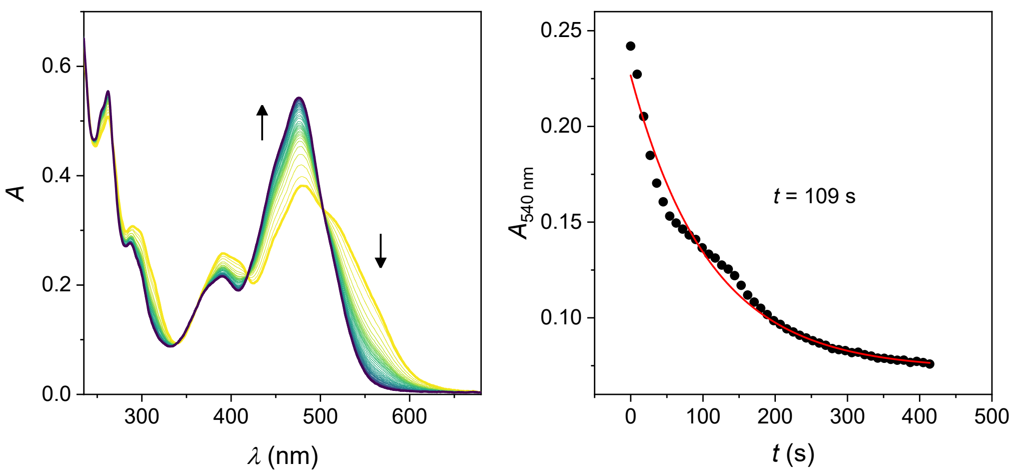

Figure S25. Thermal *E*→*Z* back isomerization (at 180 K) of **6** in MTHF after irradiation at 375 nm. *Left*: evolution of the UV/Vis absorption spectra. *Right*: kinetic trace at 540 nm.

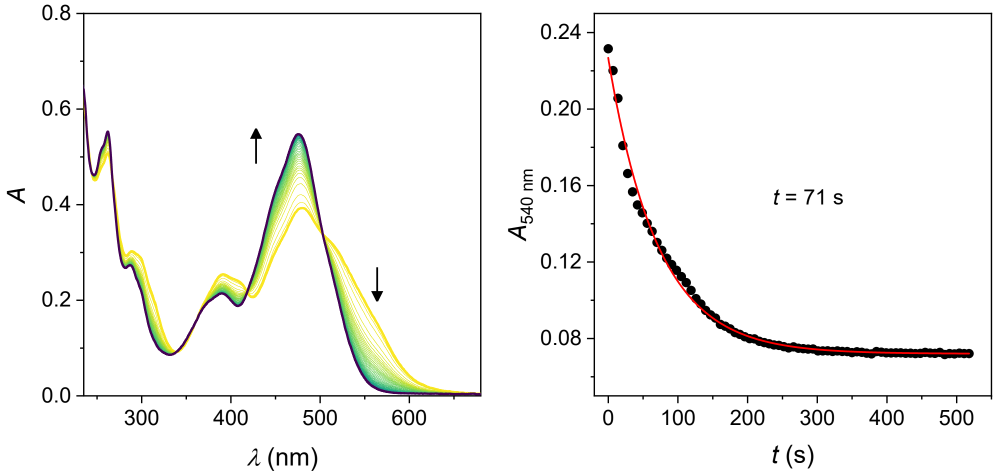

Figure S26. Thermal *E*→*Z* back isomerization (at 185 K) of **6** in MTHF after irradiation at 375 nm. *Left*: evolution of the UV/Vis absorption spectra. *Right*: kinetic trace at 540 nm.

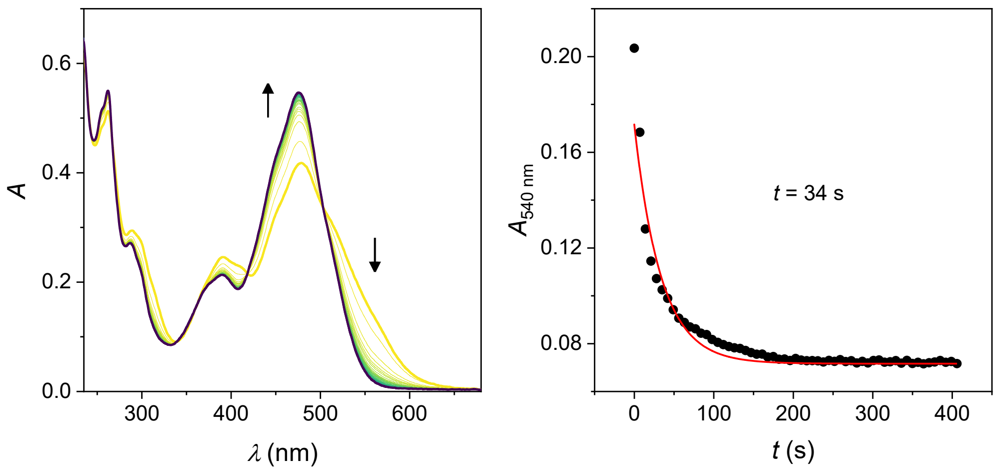

Figure S27. Thermal *E*→*Z* back isomerization (at 190 K) of **6** in MTHF after irradiation at 375 nm. *Left*: evolution of the UV/Vis absorption spectra. *Right*: kinetic trace at 540 nm.

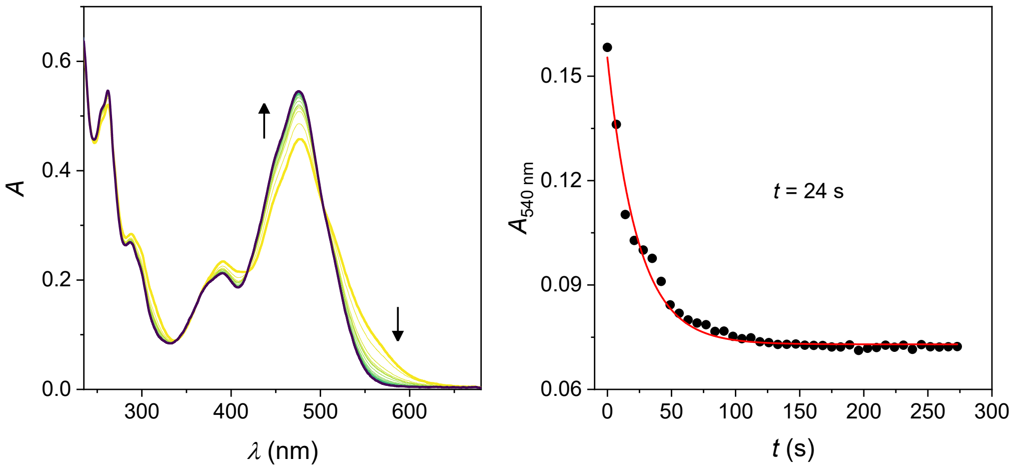

Figure S28. Thermal *E*→*Z* back isomerization (at 195 K) of **6** in MTHF after irradiation at 375 nm. *Left*: evolution of the UV/Vis absorption spectra. *Right*: kinetic trace at 540 nm.

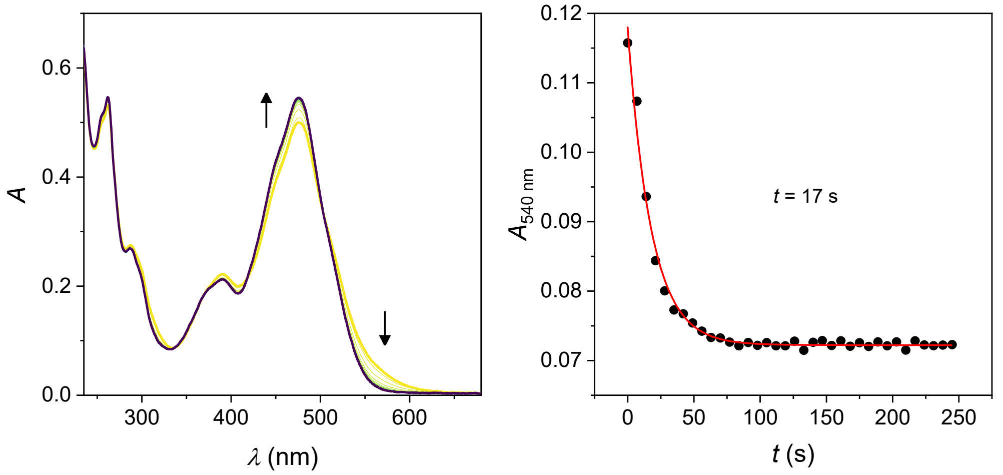

Figure S29. Thermal *E*→*Z* back isomerization (at 200 K) of **6** in MTHF after irradiation at 375 nm. *Left*: evolution of the UV/Vis absorption spectra. *Right*: kinetic trace at 540 nm.

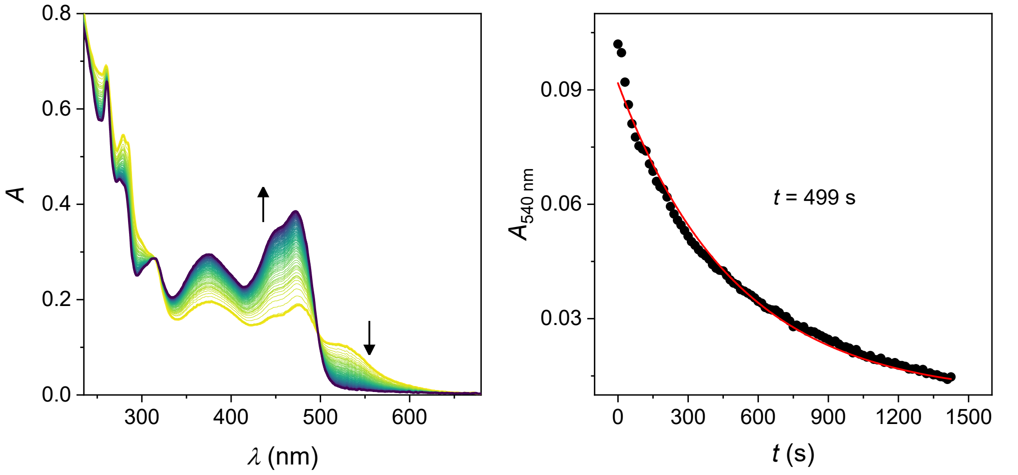

Figure S30. Thermal *E*→*Z* back isomerization (at 190 K) of **7** in MTHF after irradiation at 375 nm. *Left*: evolution of the UV/Vis absorption spectra. *Right*: kinetic trace at 540 nm.

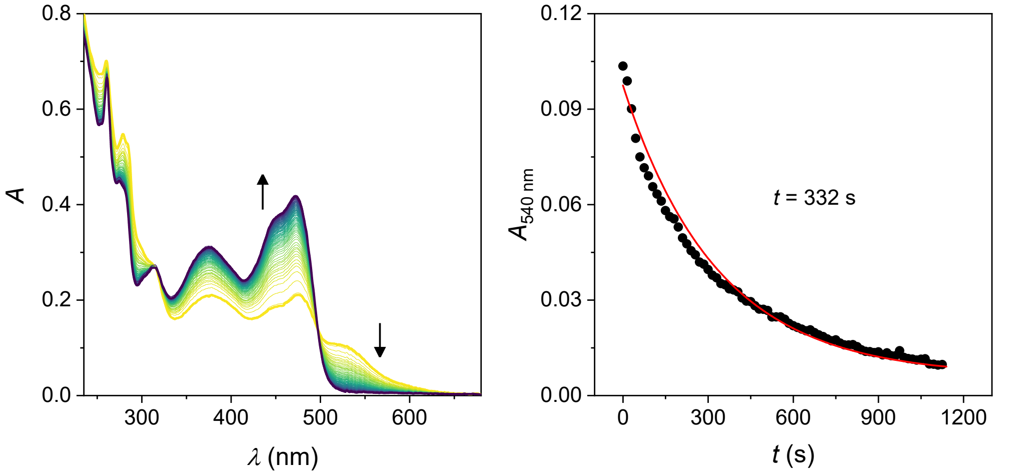

Figure S31. Thermal *E*→*Z* back isomerization (at 195 K) of **7** in MTHF after irradiation at 375 nm. *Left*: evolution of the UV/Vis absorption spectra. *Right*: kinetic trace at 540 nm.

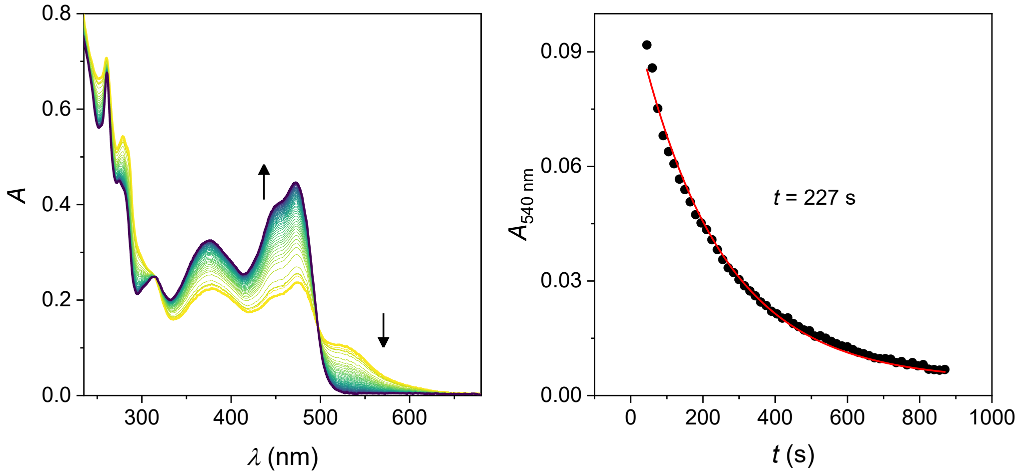

Figure S32. Thermal *E*→*Z* back isomerization (at 200 K) of **7** in MTHF after irradiation at 375 nm. *Left*: evolution of the UV/Vis absorption spectra. *Right*: kinetic trace at 540 nm.

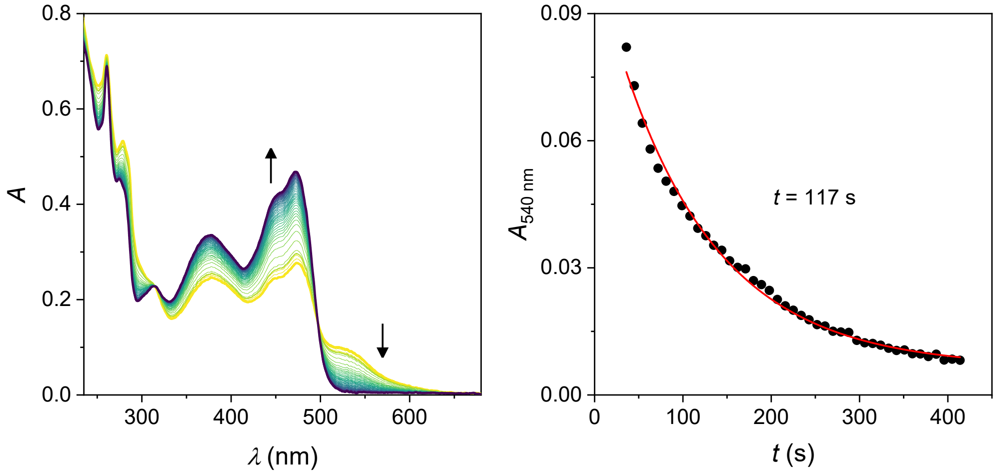

Figure S33. Thermal *E*→*Z* back isomerization (at 205 K) of **7** in MTHF after irradiation at 375 nm. *Left*: evolution of the UV/Vis absorption spectra. *Right*: kinetic trace at 540 nm.

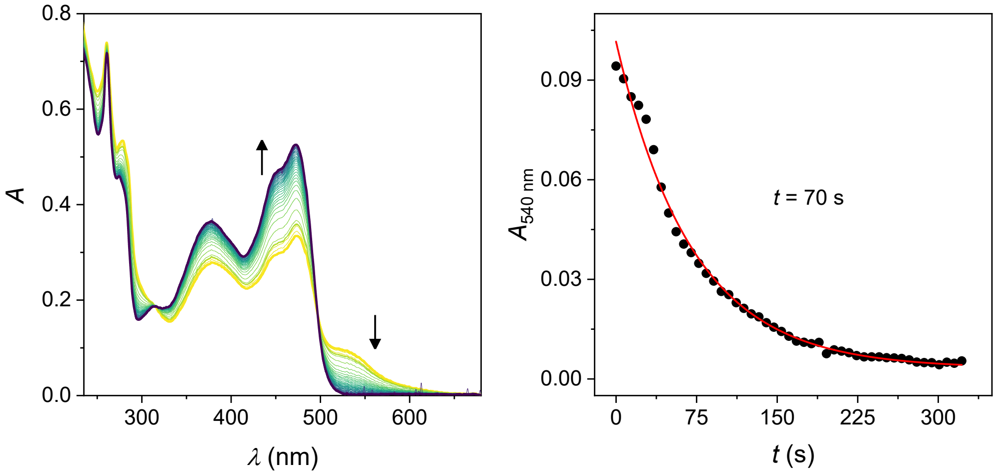

Figure S34. Thermal *E*→*Z* back isomerization (at 210 K) of **7** in MTHF after irradiation at 375 nm. *Left*: evolution of the UV/Vis absorption spectra. *Right*: kinetic trace at 540 nm.

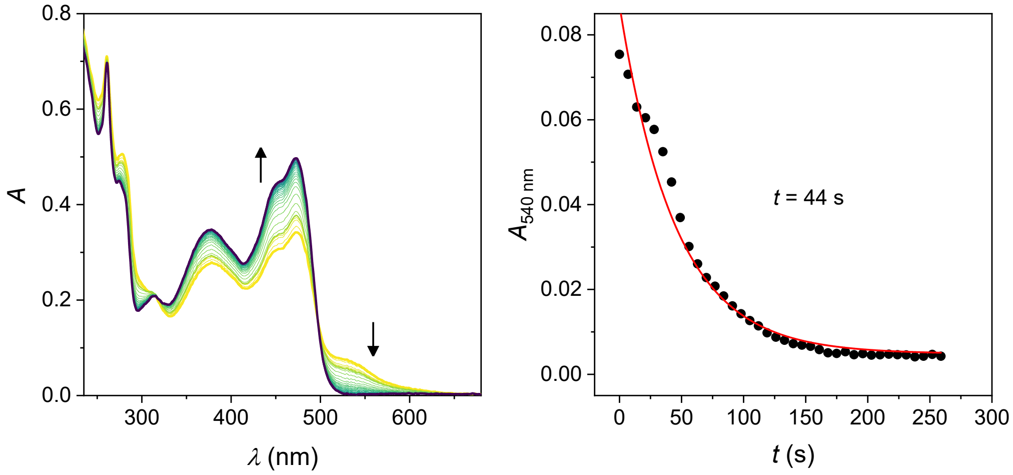

Figure S35. Thermal *E*→*Z* back isomerization (at 215 K) of **7** in MTHF after irradiation at 375 nm. *Left*: evolution of the UV/Vis absorption spectra. *Right*: kinetic trace at 540 nm.

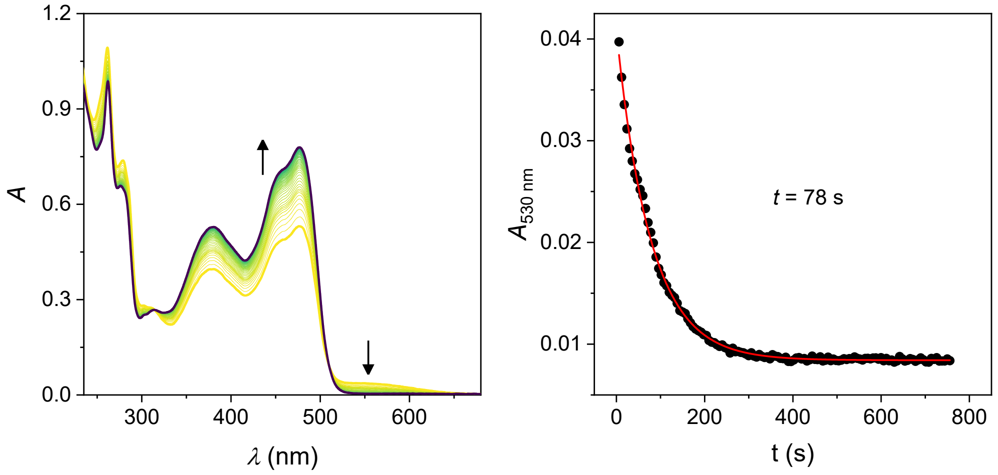

Figure S36. Thermal *E*→*Z* back isomerization (at 195 K) of **8** in MTHF after irradiation at 375 nm. *Left*: evolution of the UV/Vis absorption spectra. *Right*: kinetic trace at 530 nm.

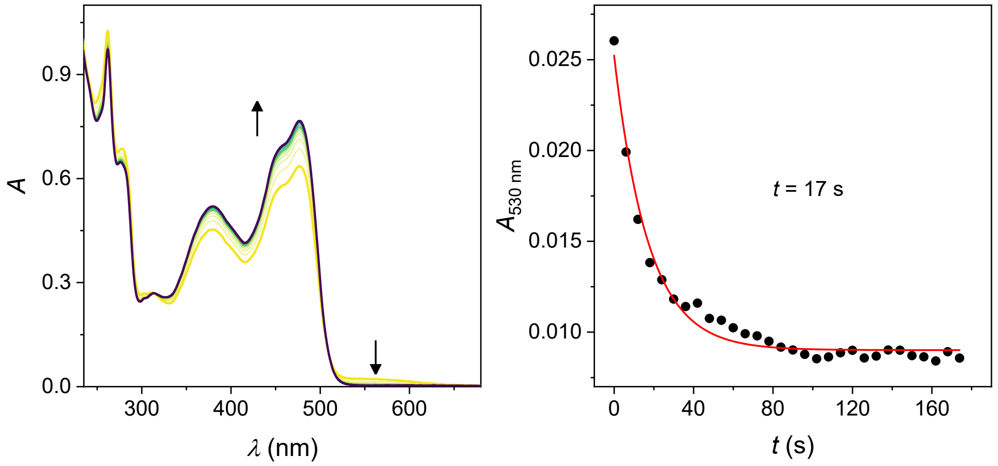

Figure S37 Thermal *E*→*Z* back isomerization (at 205 K) of **8** in MTHF after irradiation at 375 nm. *Left*: evolution of the UV/Vis absorption spectra. *Right*: kinetic trace at 530 nm.

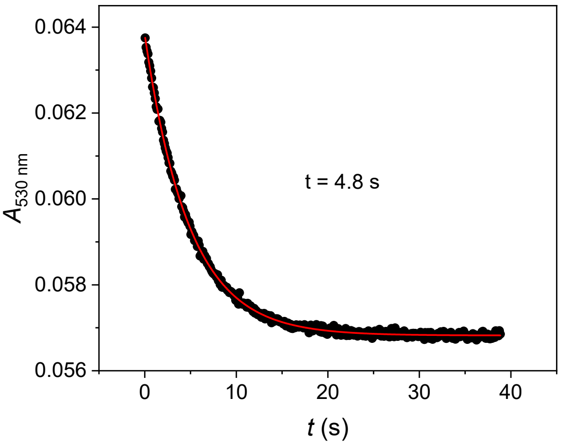

Figure S38. Kinetic trace at 530 nm of the thermal *E*→*Z* back isomerization of **8** in MTHF (at 215 K) after irradiation at 375 nm. Due to the fast back reaction only the trace was recorded but not the complete spectra.

7. Low temperature NMR irradiation

Photoswitch 6:

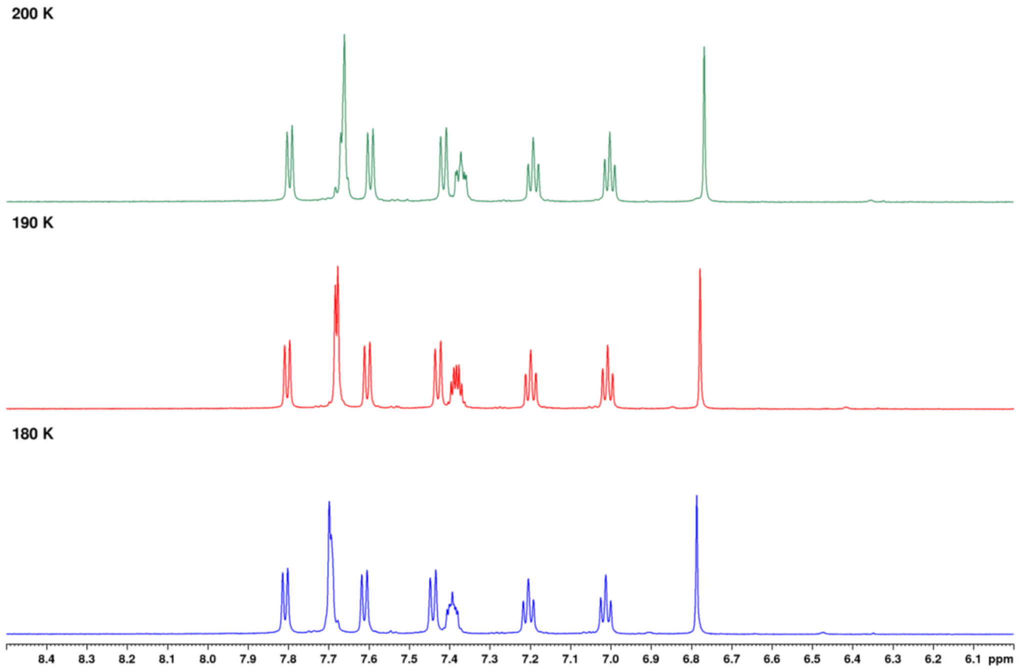

Figure S39. Partial ^{1}H NMR spectra of *Z*-**6** in THF-d_8 at various temperatures.

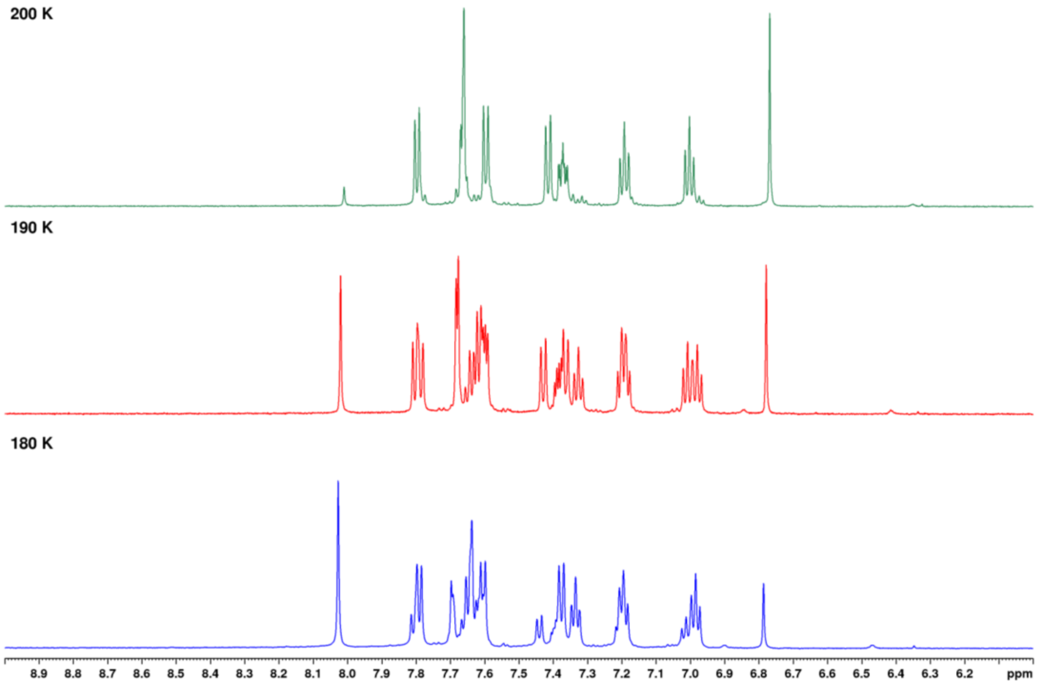

Figure S40. Partial ^{1}H NMR spectra after irradiation (450 nm) of *Z*-**6** in THF-d_8 at various temperatures. These spectra present the photostationary state.

Table S2. Photostationary state distribution of **6** at varying temperatures.

Temperature (K)	*E*-6 (%)	*Z*-6 (%)
200	10.2	89.8
190	49.6	50.4
180	77.9	22.1

Photoswitch 7:

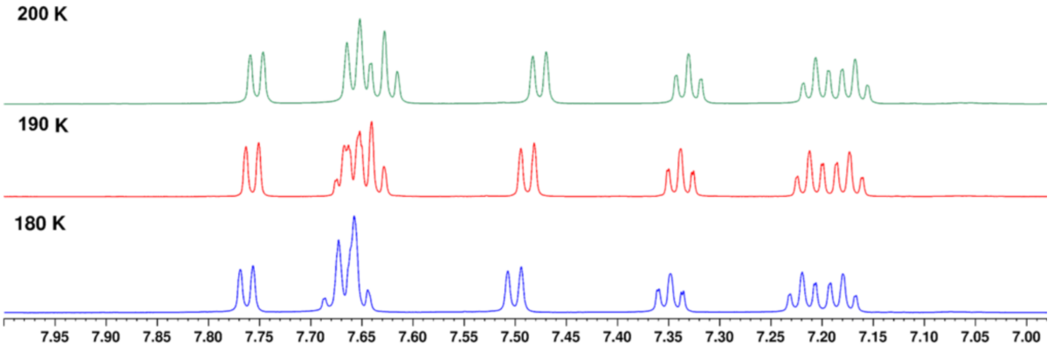

Figure S41. Partial ^{1}H NMR spectra of *Z*-**7** in THF-d_8 at various temperatures.

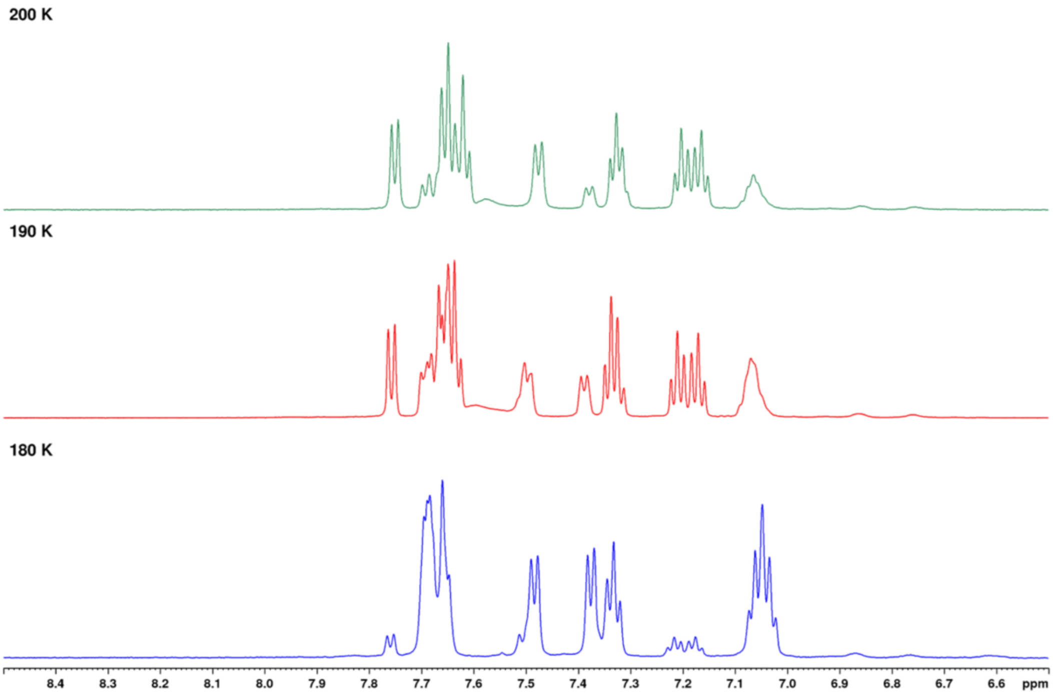

Figure S42. Partial ^{1}H NMR spectra after irradiation (450 nm) of *Z*-**7** in THF-d_8 at various temperatures. These spectra present the photostationary state.

Table S3. Photostationary state distribution of **7** at varying temperatures.

Temperature (K)	*E*-7 (%)	*Z*-7 (%)
200	26.1	73.9
190	34.8	65.2
180	82.6	17.4

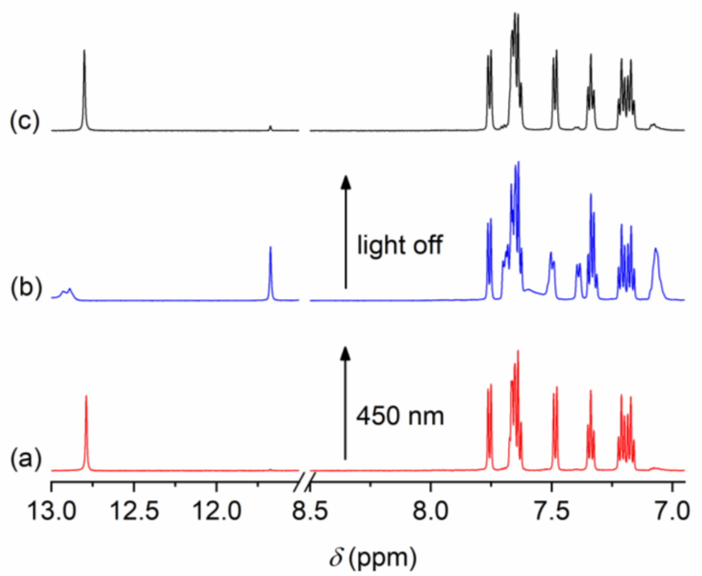

Figure S43. Partial ^{1}H NMR spectra of **7** in THF-d_8 at 190 K, demonstrating the reversible T-type photoswitching. (a) *Z*-**7** before irradiation, (b) photothermal stationary state upon irradiation with 450-nm light (*Z*/*E* *ca*. 65/35), (c) spectrum after thermal back isomerization in the dark, corresponding to *Z*-**7**.

Photoswitch 8:

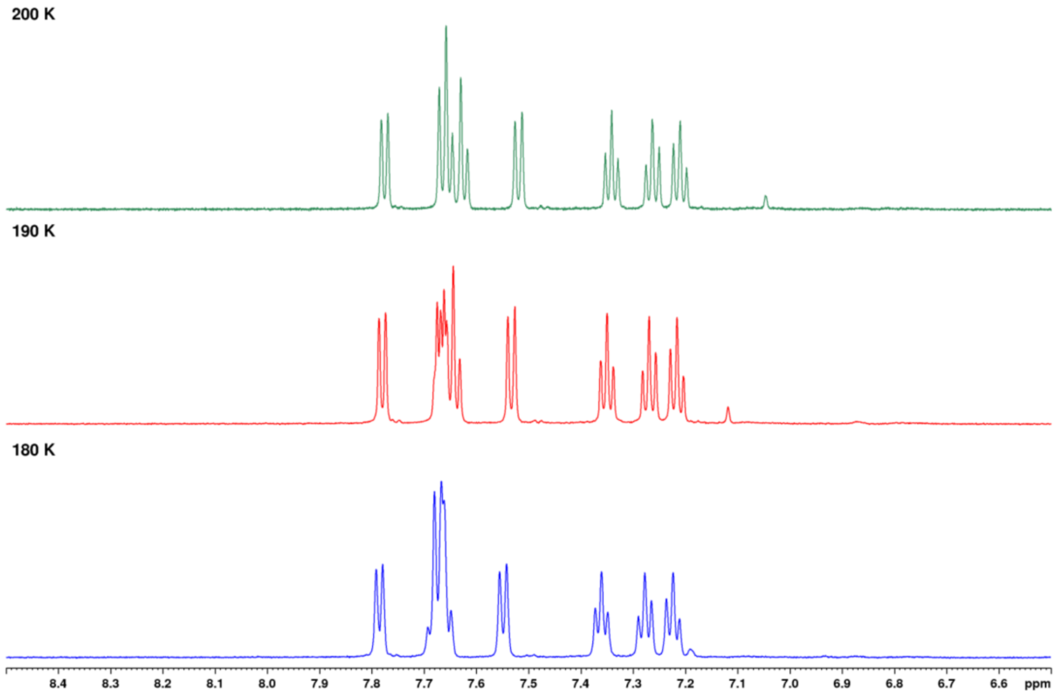

Figure S44. Partial ^{1}H NMR spectra of *Z*-**8** in THF-*d*$_8$ at various temperatures.

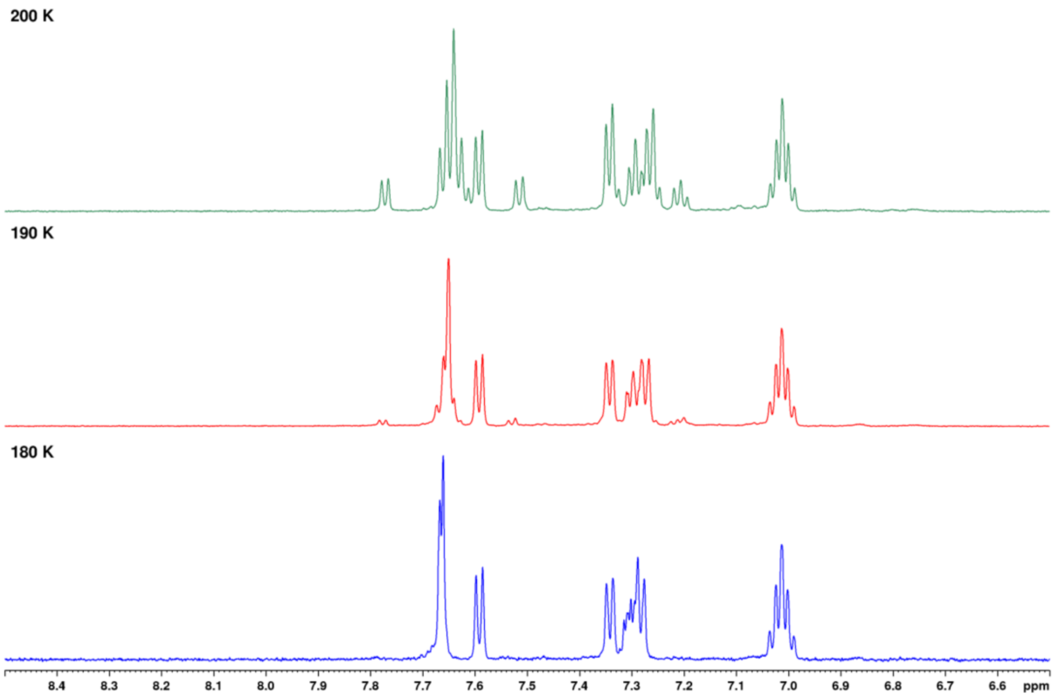

Figure S45. Partial ^{1}H NMR spectra after irradiation (450 nm) of *Z*-**8** in THF-*d*$_8$ at various temperatures. These spectra present the photostationary state.

Table S4. Photostationary state distribution of **8** at varying temperatures.

Temperature (K)	*E*-8 (%)	*Z*-8 (%)
200	72.7	27.3
190	93.6	6.4
180	99.2	0.8

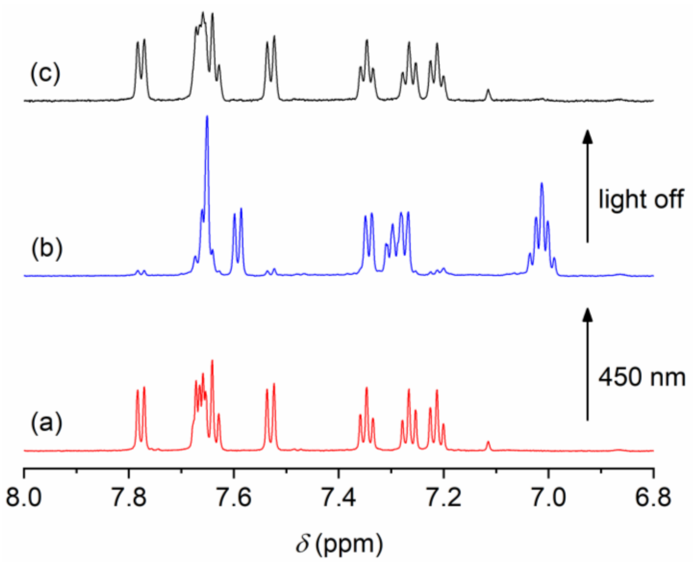

Figure S46. Partial ^{1}H NMR spectra of **8** in THF-d_8 at 190 K, demonstrating the reversible T-type photoswitching. (a) *Z*-**8** before irradiation, (b) photothermal stationary state upon irradiation with 450-nm light (*Z*/*E* *ca*. 6/94), (c) spectrum after thermal back isomerization in the dark, corresponding to *Z*-**8**.

8. Crystallographic data

Table S5. Crystal data and structure refinement for **6** (CCDC 2431430).

Empirical formula	$C_{17}H_{12}N_2OS$	
Formula weight	292.35	
Temperature	150.00(10) K	
Wavelength	1.54184 Å	
Crystal system	Monoclinic	
Space group	C 1 2/c 1	
Unit cell dimensions	a = 13.64030(10) Å	a= 90°.
	b = 7.84000(10) Å	b= 94.1020(10)°.
	c = 25.3454(2) Å	g = 90°.
Volume	2703.49(5) Å^3	
Z	8	
Density (calculated)	1.437 Mg/m^3	
Absorption coefficient	2.118 mm^{-1}	
F(000)	1216	
Crystal size	0.16 × 0.15 × 0.1 mm^3	
Theta range for data collection	3.497 to 79.839°.	
Index ranges	–15<=h<=17, –9<=k<=9, –32<=l<=31	
Reflections collected	26066	
Independent reflections	2929 [R(int) = 0.0282]	
Completeness to theta = 67.684°	100.0 %	
Absorption correction	Semi-empirical from equivalents	
Max. and min. transmission	1.00000 and 0.78909	
Refinement method	Full-matrix least-squares on F^2	
Data / restraints / parameters	2929 / 0 / 192	
Goodness-of-fit on F^2	1.136	
Final R indices [I>2sigma(I)]	R1 = 0.0333, wR2 = 0.0921	
R indices (all data)	R1 = 0.0336, wR2 = 0.0923	
Extinction coefficient	0.00029(6)	
Largest diff. peak and hole	0.307 and –0.312 e·Å^{-3}	

Table S6. Crystal data and structure refinement for **7** (CCDC 2431431).

Empirical formula	$C_{15}H_9N_3OS$	
Formula weight	279.31	
Temperature	150.00(10) K	
Wavelength	0.71073 Å	
Crystal system	Monoclinic	
Space group	I 1 2/a 1	
Unit cell dimensions	a = 19.7360(4) Å	a= 90°.
	b = 4.87140(10) Å	b= 101.070(2)°.
	c = 26.2576(4) Å	g = 90°.
Volume	2477.49(8) Å^3	
Z	8	
Density (calculated)	1.498 Mg/m^3	
Absorption coefficient	0.259 mm^{-1}	
F(000)	1152	
Crystal size	0.21 × 0.055 × 0.05 mm^3	
Theta range for data collection	2.376 to 33.846°.	
Index ranges	–29<=h<=26, –7<=k<=7, –39<=l<=39	
Reflections collected	33247	
Independent reflections	4492 [R(int) = 0.0301]	
Completeness to theta = 25.242°	99.9 %	
Absorption correction	Semi-empirical from equivalents	
Max. and min. transmission	1.00000 and 0.87132	
Refinement method	Full-matrix least-squares on F^2	
Data / restraints / parameters	4492 / 0 / 185	
Goodness-of-fit on F^2	1.045	
Final R indices [I>2sigma(I)]	R1 = 0.0380, wR2 = 0.1024	
R indices (all data)	R1 = 0.0469, wR2 = 0.1070	
Extinction coefficient	n/a	
Largest diff. peak and hole	0.444 and –0.205 e.Å^{-3}	

Additional details: The H2 atom on N2 was located in the difference Fourier map and freely refined, maintaining reasonable geometry and bond lengths consistent with an N–H group.

Table S7. Crystal data and structure refinement for **8** (CCDC 2431432).

Empirical formula	$C_{16}H_{11}N_3OS$	
Formula weight	293.34	
Temperature	150.00(10) K	
Wavelength	1.54184 Å	
Crystal system	Triclinic	
Space group	P-1	
Unit cell dimensions	a = 7.62660(10) Å	a= 81.1440(10)°.
	b = 7.92250(10) Å	b= 82.1160(10)°.
	c = 24.4377(3) Å	g = 64.1260(10)°.
Volume	1308.64(3) $Å^3$	
Z	4	
Density (calculated)	1.489 Mg/m^3	
Absorption coefficient	2.213 mm^{-1}	
F(000)	608	
Crystal size	0.129 × 0.121 × 0.046 mm^3	
Theta range for data collection	3.672 to 77.130°.	
Index ranges	–9<=h<=9, –9<=k<=10, –30<=l<=30	
Reflections collected	55004	
Independent reflections	5503 [R(int) = 0.0436]	
Completeness to theta = 67.684°	100.0 %	
Absorption correction	Gaussian	
Max. and min. transmission	1.000 and 0.736	
Refinement method	Full-matrix least-squares on F^2	
Data / restraints / parameters	5503 / 0 / 381	
Goodness-of-fit on F^2	1.056	
Final R indices [I>2sigma(I)]	R1 = 0.0341, wR2 = 0.0917	
R indices (all data)	R1 = 0.0357, wR2 = 0.0929	
Extinction coefficient	n/a	
Largest diff. peak and hole	0.278 and –0.439 e·$Å^{-3}$	

Additional details: This compound crystallized in the triclinic space group *P*-1, and two molecules were found in the asymmetric unit.

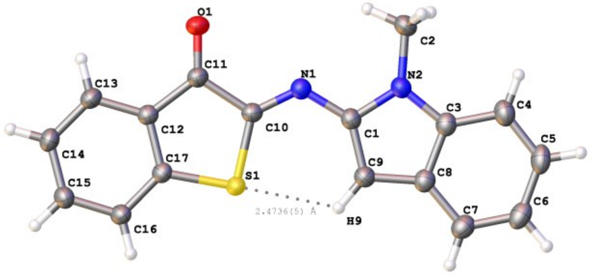

Figure S47. Molecular structure of **6** with displacement ellipsoids at 50% probability. Selected bond lengths (Å) and angles (°): S1···H9 2.4736(5), S(1)-C(10) 1.7608(13), S(1)-C(17) 1.7630(13), O(1)-C(11) 1.2112(17), N(1)-C(1) 1.3758(17), N(1)-C(10) 1.2807(18), N(2)-C(1) 1.3900(17), N(2)-C(2) 1.4547(17), N(2)-C(3) 1.3702(18), C(1)-C(9) 1.3898(19), C(3)-C(4) 1.399(2), C(3)-C(8) 1.415(2), C(4)-C(5) 1.379(2), C(5)-C(6) 1.401(2), C(6)-C(7) 1.376(2), C(7)-C(8) 1.4102(19), C(8)-C(9) 1.4148(19), C(10)-C(11) 1.5241(18), C(11)-C(12) 1.4679(19), C(12)-C(13) 1.3936(19), C(12)-C(17) 1.3947(18), C(13)-C(14) 1.383(2), C(14)-C(15) 1.393(2), C(15)-C(16) 1.3909(19), C(16)-C(17) 1.3822(19), C(10)-S(1)-C(17) 92.33(6), C(10)-N(1)-C(1) 123.08(12), C(1)-N(2)-C(2) 126.19(12), C(3)-N(2)-C(1) 108.65(11), C(3)-N(2)-C(2) 125.09(12), N(1)-C(1)-N(2) 116.86(12), N(1)-C(1)-C(9) 134.32(13), C(9)-C(1)-N(2) 108.83(12), N(2)-C(3)-C(4) 129.88(14), N(2)-C(3)-C(8) 108.21(12), N(1)-C(10)-S(1) 128.50(11), N(1)-C(10)-C(11) 120.98(12), C(11)-C(10)-S(1) 110.52(9), O(1)-C(11)-C(10) 123.25(12), O(1)-C(11)-C(12) 126.82(13), C(12)-C(17)-S(1) 113.96(10), C(16)-C(17)-S(1) 124.39(10), C(16)-C(17)-C(12) 121.64(12).

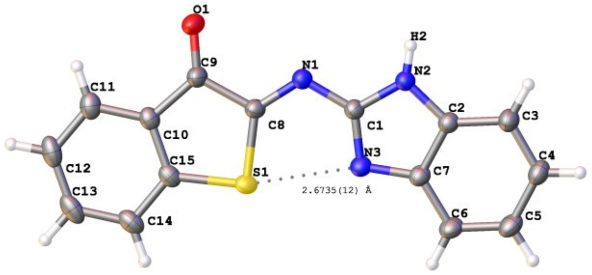

Figure S48. Molecular structure of **7** with displacement ellipsoids at 50% probability. Selected bond lengths (Å) and angles (°): S(1)···N(3) 2.674(1), S(1)-C(15) 1.7696(11), S(1)-C(8) 1.7500(10), O(1)-C(9) 1.2097(14), N(2)-C(1) 1.3639(13), N(2)-C(2) 1.3765(14), N(2)-H(2) 0.826(17), N(3)-C(1) 1.3238(13), N(3)-C(7) 1.3756(14), N(1)-C(1) 1.3805(13), N(1)-C(8) 1.2893(13), C(10)-C(9) 1.4646(15), C(10)-C(15) 1.3917(16), C(10)-C(11) 1.3921(16), C(9)-C(8) 1.5166(15), C(7)-C(2) 1.4117(14), C(7)-C(6) 1.3992(15), C(15)-C(14) 1.3897(14), C(2)-C(3) 1.3981(15), C(3)-C(4) 1.3839(16), C(14)-C(13) 1.3887(18), C(6)-C(5) 1.3746(17), C(11)-C(12) 1.3906(17), C(5)-C(4) 1.4072(18), C(12)-C(13) 1.392(2), C(8)-S(1)-C(15) 91.13(5), C(1)-N(2)-C(2) 106.70(9), C(1)-N(3)-C(7) 104.41(9), C(8)-N(1)-C(1) 117.67(9), O(1)-C(9)-C(10) 127.12(11), O(1)-C(9)-C(8) 122.76(10), N(2)-C(1)-N(1) 120.37(9), N(3)-C(1)-N(2) 113.49(9), N(3)-C(1)-N(1) 126.14(9), N(3)-C(7)-C(2) 110.45(9), N(3)-C(7)-C(6) 129.21(10), C(10)-C(15)-S(1) 115.10(8), C(14)-C(15)-S(1) 124.20(10), N(2)-C(2)-C(7) 104.94(9), N(2)-C(2)-C(3) 133.36(10), N(1)-C(8)-S(1) 129.23(8), N(1)-C(8)-C(9) 119.21(9), C(9)-C(8)-S(1) 111.56(7).

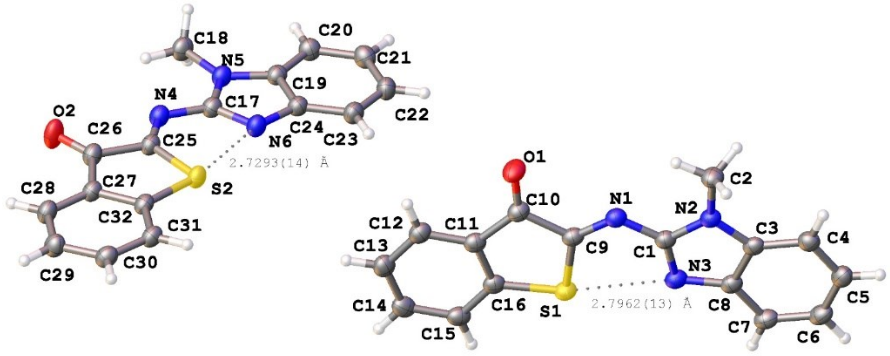

Figure S49. Molecular structure of **8** showing the two independent molecules found within the asymmetric cell with displacement ellipsoids at 50% probability. Selected bond lengths (Å) and angles (°): S(1)···N(3) 2.796(1), S(2)···N(6) 2.729(1), C(20)-C(21) 1.382(2), C(21)-C(22) 1.407(2), C(22)-C(23) 1.379(2), C(23)-C(24) 1.400(2), C(25)-C(26) 1.527(2), C(26)-C(27) 1.466(2), C(27)-C(28) 1.386(2), C(27)-C(32) 1.3961(19), C(28)-C(29) 1.390(2), C(29)-C(30) 1.393(2), C(30)-C(31) 1.387(2), C(31)-C(32) 1.387(2), C(9)-S(1)-C(16) 91.14(7), C(9)-N(1)-C(1) 120.20(12), C(1)-N(2)-C(2) 127.22(12), C(1)-N(2)-C(3) 106.34(11), C(3)-N(2)-C(2) 126.22(12), C(1)-N(3)-C(8) 104.12(12), N(2)-C(1)-N(1) 118.27(12), N(3)-C(1)-N(1) 128.04(13), N(3)-C(1)-N(2) 113.66(12), N(2)-C(3)-C(4) 131.41(13), N(2)-C(3)-C(8) 105.64(12), C(4)-C(3)-C(8) 122.95(13), C(5)-C(4)-C(3) 116.58(14), C(4)-C(5)-C(6) 121.42(15), C(7)-C(6)-C(5) 121.82(14), C(6)-C(7)-C(8) 117.90(14), N(3)-C(8)-C(3) 110.23(12), N(3)-C(8)-C(7) 130.42(14), C(7)-C(8)-C(3) 119.34(14), N(1)-C(9)-S(1) 129.09(11), N(1)-C(9)-C(10) 119.62(12), C(10)-C(9)-S(1) 111.28(10), O(1)-C(10)-C(9) 123.01(13), O(1)-C(10)-C(11) 126.96(14), C(11)-C(10)-C(9) 110.01(12), C(12)-C(11)-C(10) 126.51(13), C(12)-C(11)-C(16) 121.04(14), C(16)-C(11)-C(10) 112.41(13), C(13)-C(12)-C(11) 118.72(14), C(12)-C(13)-C(14) 119.78(14), C(15)-C(14)-C(13) 121.89(14), C(16)-C(15)-C(14) 117.88(14), C(11)-C(16)-S(1) 114.94(11), C(15)-C(16)-S(1) 124.40(11), C(15)-C(16)-C(11) 120.65(14), C(25)-S(2)-C(32) 91.33(7), C(25)-N(4)-C(17) 118.94(13), C(17)-N(5)-C(18) 127.78(12), C(17)-N(5)-C(19) 106.13(12), C(19)-N(5)-C(18) 126.08(12), C(17)-N(6)-C(24) 104.39(12), N(5)-C(17)-N(4) 119.22(13), N(6)-C(17)-N(4) 127.22(13), N(6)-C(17)-N(5) 113.56(13), N(5)-C(19)-C(20) 131.72(13), N(5)-C(19)-C(24) 105.69(12), C(20)-C(19)-C(24) 122.58(13), C(21)-C(20)-C(19) 116.74(14), C(20)-C(21)-C(22) 121.49(14), C(23)-C(22)-C(21) 121.64(14), C(22)-C(23)-C(24) 117.81(14), N(6)-C(24)-C(19) 110.23(12), N(6)-C(24)-C(23) 130.04(14), C(23)-C(24)-C(19) 119.73(14), N(4)-C(25)-S(2) 128.94(11), N(4)-C(25)-C(26) 119.64(12), C(26)-C(25)-S(2) 111.42(10), O(2)-C(26)-C(25) 122.90(13), O(2)-C(26)-C(27) 127.25(14), C(27)-C(26)-C(25) 109.84(12), C(28)-C(27)-C(26) 126.76(13), C(28)-C(27)-C(32) 120.69(14), C(32)-C(27)-C(26) 112.56(13), C(27)-C(28)-C(29) 118.72(14), C(28)-C(29)-C(30) 120.10(14), C(31)-C(30)-C(29) 121.56(14), C(32)-C(31)-C(30) 117.97(13), C(27)-C(32)-S(2) 114.71(11), C(31)-C(32)-S(2) 124.36(11), C(31)-C(32)-C(27) 120.92(14).

9. Theoretical calculations

Photoswitch 6

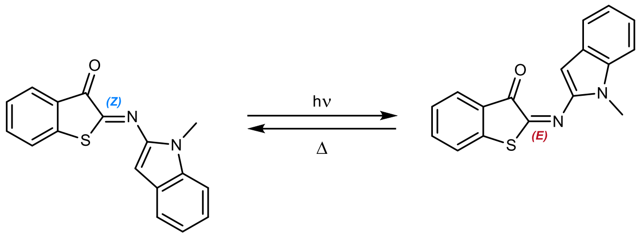

Table S8. Energies of the optimized structures of the isomeric forms of **6**.

Solvent	Energy *Z* (kJ/mol)	Energy *E* (kJ/mol)	$\Delta E_{Z\text{-}E}$ (kJ/mol)
ACN	–3249977.7803	–3249953.7804	24.0
THF	–3249972.3561	–3249949.5981	22.8
**MTHF**	**–3249918.7168**	**–3249897.4270**	**21.3**

Theory level: SMD(*solvent*)/M06-2X/6-311+G(d,p)

Table S9. Relevant data for the $S_0 \rightarrow S_1$ transition of **6** in various solvents.

Solvent	Isomer *Z*				Isomer *E*			
	f	*Comp.*	*E* (eV)	*λ* (nm)	*f*	*Comp.*	*E* (eV)	*λ* (nm)
ACN	0.7673	H→L (99)	2.42	512	0.6765	H→L (99)	2.23	556
THF	0.6826	H→L (99)	2.48	500	0.6181	H→L (99)	2.28	545
**MTHF**	**0.6682**	**H→L (99)**	**2.48**	**500**	**0.6097**	**H→L (99)**	**2.27**	**546**

Theory level: SMD(*solvent*)/mPW1PW91/6-311+G(d,p)

Photoswitch 7

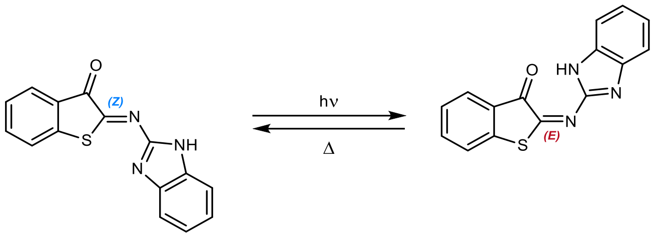

Table S10. Energies of the optimized structures of the isomeric forms of **7**.

Solvent	Energy *Z* (kJ/mol)	Energy *E* (kJ/mol)	$\Delta E_{Z\text{-}E}$ (kJ/mol)
ACN	–3188973.5009	–3188941.8949	31.6
THF	–3188966.6270	–3188934.6696	32.0
**MTHF**	**–3188918.4834**	**–3188892.5827**	**25.9**

Theory level: SMD(*solvent*)/M06-2X/6-311+G(d,p)

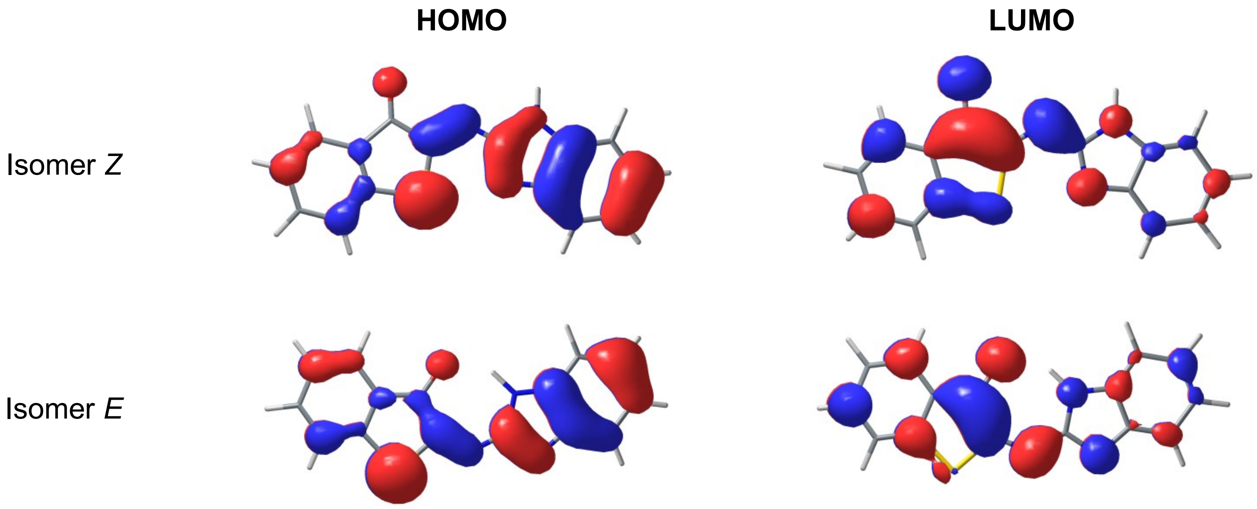

Figure S50. Frontier molecular orbitals participating in the $S_0 \rightarrow S_1$ transition of **7**; theory level: SMD(MTHF)/mPW1PW91/6-311+G(d,p).

Table S11. Relevant data for the $S_0 \rightarrow S_1$ transition of **7** in various solvents.

Solvent	Isomer *Z*				Isomer *E*			
	f	*Comp.*	*E* (eV)	*λ* (nm)	*f*	*Comp.*	*E* (eV)	*λ* (nm)
ACN	0.6215	H→L (93)	2.58	480	0.5515	H→L (96)	2.27	546
THF	0.5726	H→L (92)	2.63	472	0.5191	H→L (95)	2.29	542
**MTHF**	**0.5699**	**H→L (92)**	**2.62**	**473**	**0.5146**	**H→L (95)**	**2.28**	**545**

Theory level: SMD(*solvent*)/mPW1PW91/6-311+G(d,p)

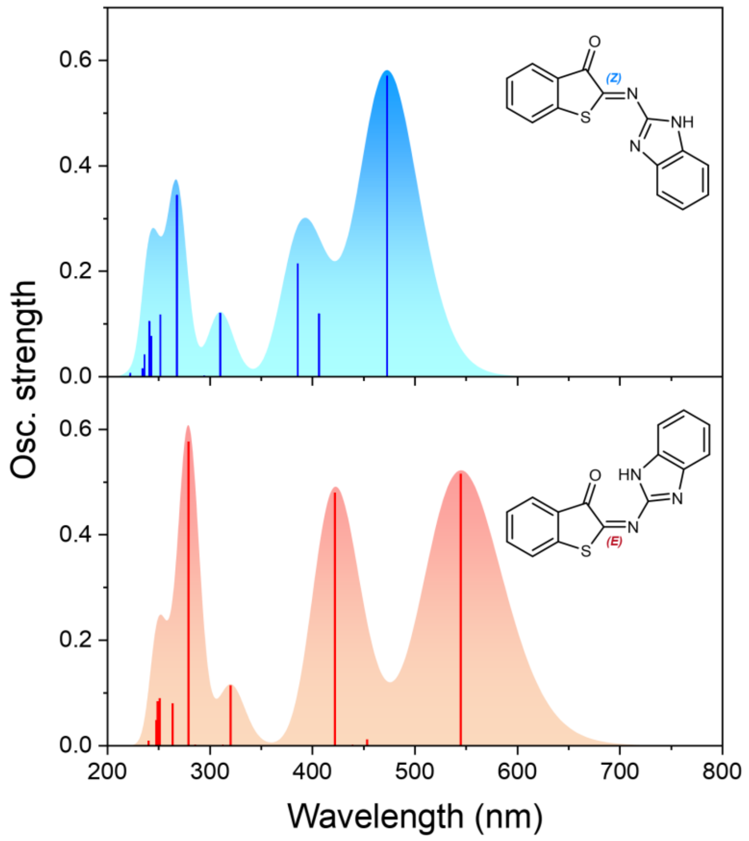

Figure S51. Predicted absorption spectra of compound **7** in both isomeric forms at the SMD(MTHF)/mPW1PW91/6-311+G(d,p) level.

Photoswitch 8

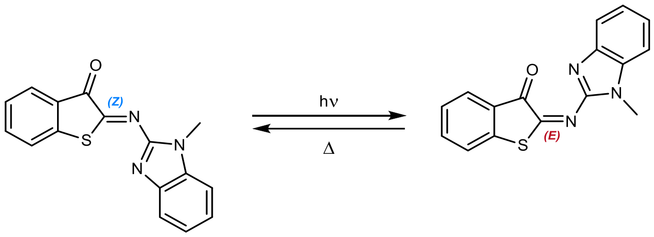

Table S12. Energies of the optimized structures of the isomeric forms of **8**.

Solvent	Energy *Z* (kJ/mol)	Energy *E* (kJ/mol)	$\Delta E_{Z\text{-}E}$ (kcal/mol)
ACN	–3292153.3299	–3292115.2154	38.1
THF	–3292148.0184	–3292107.2888	40.7
**MTHF**	**–3292093.1745**	**–3292070.2248**	**23.0**

Theory level: SMD(*solvent*)/M06-2X/6-311+G(d,p)

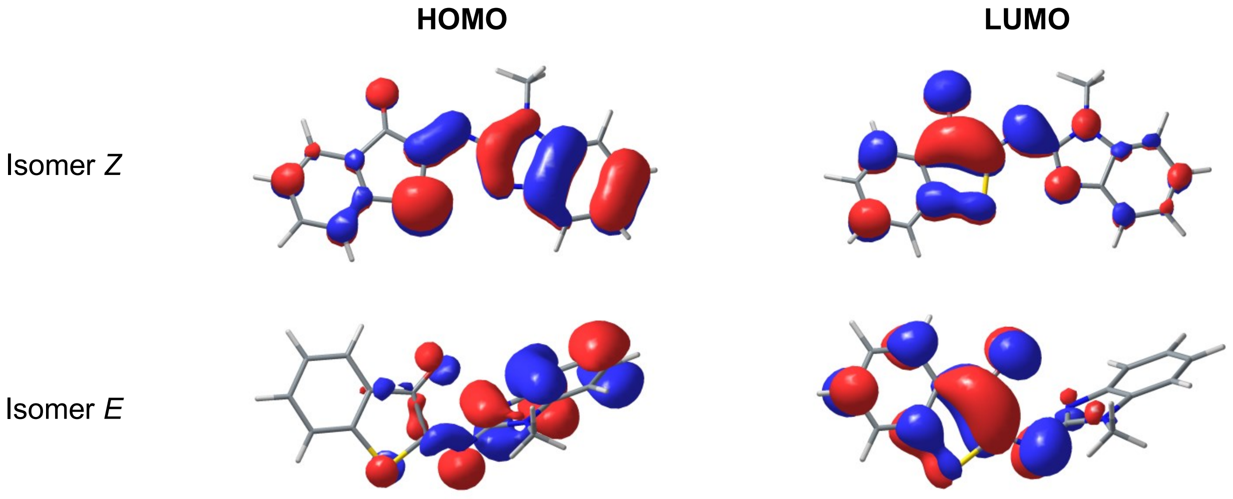

Figure S52. Frontier molecular orbitals participating in the $S_0 \rightarrow S_1$ transition of **8**; theory level: SMD(MTHF)/mPW1PW91/6-311+G(d,p).

Table S13. Relevant data for the $S_0 \rightarrow S_1$ transition of **8** in various solvents.

Solvent	Isomer *Z*				Isomer *E*			
	f	*Comp.*	*E* (eV)	*λ* (nm)	*f*	*Comp.*	*E* (eV)	*λ* (nm)
ACN	0.6453	H→L (94)	2.56	485	0.2664	H→L (92)	2.14	579
THF	0.5959	H→L (93)	2.60	477	0.2592	H→L (91)	2.15	577
**MTHF**	**0.5932**	**H→L (93)**	**2.60**	**478**	**0.0924**	**H→L (93)**	**2.10**	**591**

Theory level: SMD(*solvent*)/mPW1PW91/6-311+G(d,p)

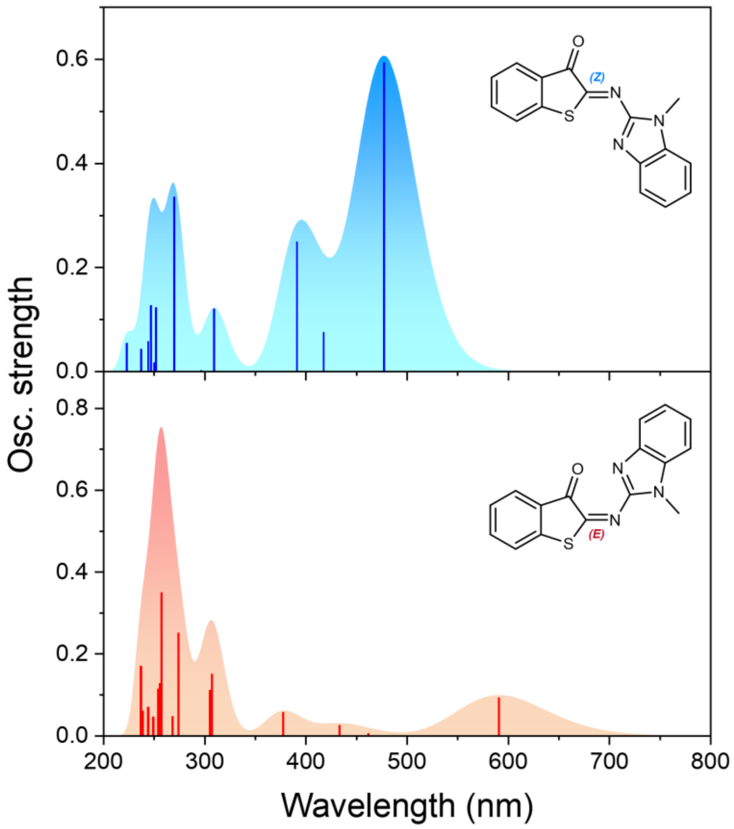

Figure S53. Predicted absorption spectra of compound **8** in both isomeric forms at the SMD(MTHF)/mPW1PW91/6-311+G(d,p) level.

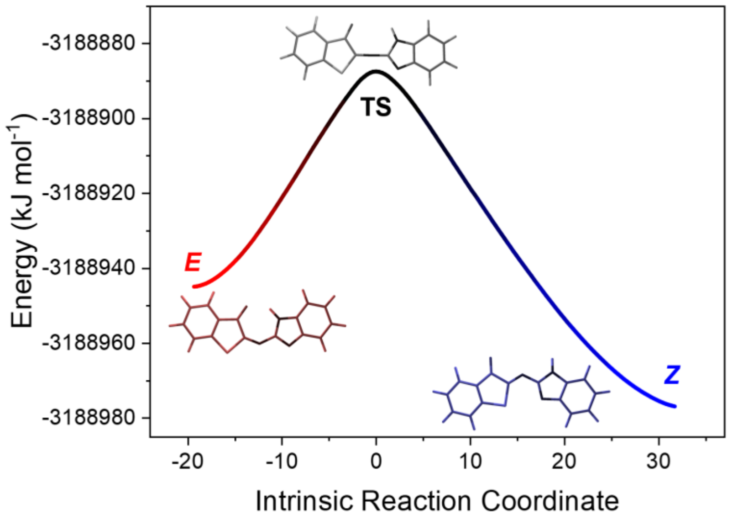

Figure S54. Calculated *E*→*Z* ground-state isomerization path for photoswitch **7**; SMD(MTHF)/M06-2X/6-311+G(d,p).

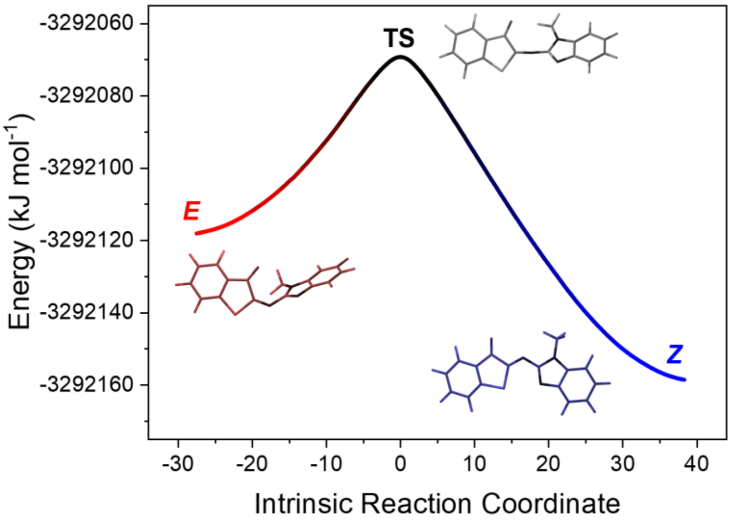

Figure S55. Calculated *E*→*Z* ground-state isomerization path for photoswitch **8**; SMD(MTHF)/M06-2X/6-311+G(d,p).

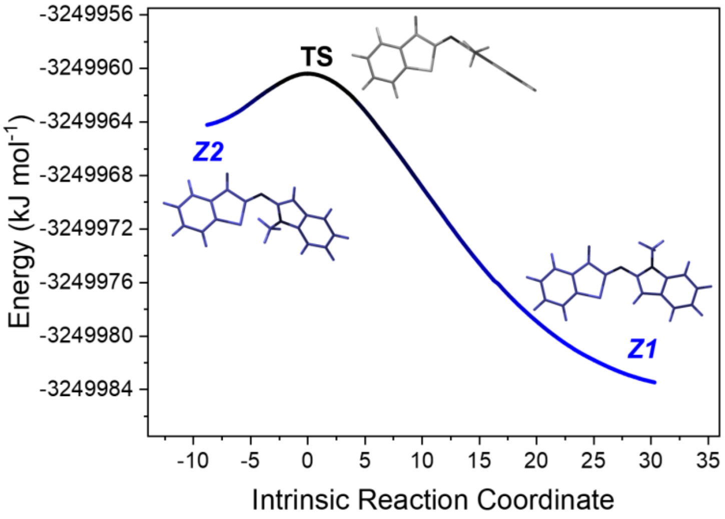

Figure S56. Calculated path for the interconversion between different rotamers of *Z*-**6** (**6***Z*2→**6***Z*1); SMD(MTHF)/M06-2X/6-311+G(d,p).

Atomic coordinates

Photoswitch **6**, isomer *Z*

- SMD(MTHF)/M06-2X/6-311+G(d,p)
- Imaginary frequencies: 0

Atomic type		Coordinates		
		X	Y	Z
1	C	-3.470110	0.480880	0.000096
2	C	-3.169280	-0.878730	-0.000170
3	C	-4.174590	-1.834060	-0.000467
4	C	-5.495720	-1.391070	-0.000496
5	C	-5.808120	-0.029720	-0.000227
6	C	-4.791500	0.916090	0.000074
7	C	-1.017180	0.480820	0.000280
8	H	-3.944910	-2.892450	-0.000671
9	H	-6.295270	-2.122560	-0.000729
10	H	-6.844000	0.284920	-0.000255
11	H	-5.004170	1.979270	0.000292
12	C	-2.276880	1.342170	0.000390
13	O	-2.253770	2.546700	0.000681
14	S	-1.428530	-1.243880	-0.000061
15	N	0.126040	1.036590	0.000369
16	C	1.331660	0.385960	0.000258
17	C	3.127550	-0.978130	0.000335
18	C	3.568280	0.369280	-0.000141
19	C	4.082990	-2.011620	0.000459
20	C	4.927740	0.710380	-0.000516
21	C	5.421740	-1.677930	0.000096
22	H	3.767580	-3.048690	0.000829
23	C	5.839120	-0.327480	-0.000388
24	H	5.248380	1.745380	-0.000865
25	H	6.171220	-2.460310	0.000177
26	H	6.899330	-0.103310	-0.000656
27	N	2.472560	1.181450	-0.000141
28	C	2.532270	2.632540	-0.000635
29	H	3.055620	2.984540	-0.891200
30	H	1.520330	3.026760	-0.000471
31	H	3.056130	2.985130	0.889390
32	C	1.705770	-0.949760	0.000568
33	H	1.063790	-1.814200	0.001010

Photoswitch **6**, isomer *E*

- SMD(MTHF)/M06-2X/6-311+G(d,p)
- Imaginary frequencies: 0

Atomic type		Coordinates		
		X	Y	Z
1	C	-3.130460	-0.721420	0.000208
2	C	-3.618930	0.585230	-0.000149
3	C	-4.986670	0.831740	0.000074
4	C	-5.849390	-0.259910	0.000671
5	C	-5.367600	-1.573180	0.001030
6	C	-4.001250	-1.809050	0.000785
7	C	-1.062640	0.587130	-0.000651
8	H	-5.373050	1.843600	-0.000211
9	H	-6.918760	-0.084220	0.000855
10	H	-6.063920	-2.402230	0.001500
11	H	-3.597910	-2.815490	0.001030
12	C	-1.664810	-0.806950	-0.000229
13	O	-1.010660	-1.824940	-0.000295
14	S	-2.355940	1.819390	-0.000883
15	N	0.141090	0.995980	-0.000730
16	C	1.337790	0.339620	-0.000456
17	C	3.170110	-0.978930	-0.000505
18	C	3.580280	0.378720	0.000130
19	C	4.149590	-1.991620	-0.000527
20	C	4.933100	0.749760	0.000720
21	C	5.479660	-1.628690	0.000061
22	H	3.856410	-3.035250	-0.000988
23	C	5.866180	-0.267730	0.000671
24	H	5.231680	1.791300	0.001170
25	H	6.247250	-2.393290	0.000086
26	H	6.921290	-0.020340	0.001100
27	N	2.469150	1.164330	0.000093
28	C	2.506990	2.615280	0.001230
29	H	3.025080	2.977150	-0.888690
30	H	1.489860	2.995000	0.001700
31	H	3.025430	2.975690	0.891530
32	C	1.751540	-0.986870	-0.000842
33	H	1.104280	-1.843620	-0.001480

Photoswitch **7**, isomer *Z*

- SMD(MTHF)/M06-2X/6-311+G(d,p)
- Imaginary frequencies: 0

Atomic type		Coordinates		
		X	Y	Z
1	C	-3.297090	0.490710	0.000026
2	C	-2.867100	-0.833500	0.000032
3	C	-3.779910	-1.877410	0.000149
4	C	-5.137330	-1.559970	0.000270
5	C	-5.576420	-0.234270	0.000261
6	C	-4.652840	0.802830	0.000130
7	C	-0.849870	0.698630	-0.000155
8	H	-3.453150	-2.910320	0.000138
9	H	-5.864730	-2.363290	0.000363
10	H	-6.637270	-0.018120	0.000359
11	H	-4.964870	1.841050	0.000114
12	C	-2.186900	1.453070	-0.000129
13	O	-2.257970	2.653730	-0.000243
14	S	-1.094880	-1.045650	-0.000194
15	N	0.234880	1.367060	-0.000177
16	C	1.462120	0.744190	-0.000108
17	C	3.107340	-0.613930	-0.000134
18	C	3.662410	0.683210	0.000148
19	C	3.947590	-1.733730	-0.000202
20	C	5.039360	0.909070	0.000363
21	C	5.313200	-1.515700	0.000015
22	H	3.530850	-2.733690	-0.000432
23	C	5.851050	-0.211940	0.000290
24	H	5.449710	1.911140	0.000569
25	H	5.989270	-2.362170	-0.000038
26	H	6.927080	-0.086020	0.000452
27	N	2.581320	1.528270	0.000151
28	H	2.579960	2.539330	0.000405
29	N	1.728280	-0.541720	-0.000347

Photoswitch **7**, isomer *E*

- SMD(MTHF)/M06-2X/6-311+G(d,p)
- Imaginary frequencies: 0

Atomic type		Coordinates		
		X	Y	Z
1	C	-2.850340	0.669550	0.000177
2	C	-3.447790	-0.591490	-0.000074
3	C	-4.829650	-0.722170	-0.000068
4	C	-5.595740	0.440120	0.000303
5	C	-5.005560	1.708200	0.000625
6	C	-3.624790	1.828970	0.000520
7	C	-0.903960	-0.825180	-0.000485
8	H	-5.300370	-1.697510	-0.000217
9	H	-6.676090	0.355830	0.000354
10	H	-5.629820	2.592560	0.000907
11	H	-3.139060	2.798110	0.000743
12	C	-1.389960	0.629120	-0.000069
13	O	-0.654040	1.592960	0.000012
14	S	-2.296120	-1.931190	-0.000218
15	N	0.254700	-1.349040	-0.000316
16	C	1.507670	-0.787260	-0.000368
17	C	3.643540	-0.716070	0.000372
18	C	3.199440	0.622650	-0.000538
19	C	5.018040	-0.998070	0.001090
20	C	4.081470	1.706620	-0.000712
21	C	5.893500	0.069220	0.000814
22	H	5.368230	-2.023260	0.001730
23	C	5.430140	1.404370	-0.000052
24	H	3.724580	2.728890	-0.001390
25	H	6.961200	-0.114020	0.001310
26	H	6.153360	2.211140	-0.000208
27	N	1.832720	0.544710	-0.001120
28	N	2.569850	-1.570620	0.000223
29	H	1.151240	1.297030	-0.000576

Photoswitch **8**, isomer *Z*

- SMD(MTHF)/M06-2X/6-311+G(d,p)
- Imaginary frequencies: 0

Atomic type		Coordinates		
		X	Y	Z
1	C	-3.420520	0.463130	-0.000006
2	C	-3.042900	-0.877000	0.000014
3	C	-3.996710	-1.883740	0.000061
4	C	-5.340430	-1.513020	0.000084
5	C	-5.726780	-0.170910	0.000062
6	C	-4.762870	0.828710	0.000017
7	C	-0.966800	0.574750	-0.000064
8	H	-3.710850	-2.928690	0.000078
9	H	-6.099220	-2.286790	0.000122
10	H	-6.778290	0.086860	0.000080
11	H	-5.033370	1.878590	-0.000001
12	C	-2.273240	1.380970	-0.000052
13	O	-2.298410	2.583800	-0.000071
14	S	-1.280900	-1.159680	-0.000032
15	N	0.143000	1.199080	-0.000070
16	C	1.346390	0.534510	-0.000057
17	C	2.920690	-0.905920	-0.000042
18	C	3.532380	0.364300	-0.000002
19	C	3.713490	-2.060060	-0.000033
20	C	4.917950	0.535290	0.000065
21	C	5.087590	-1.898840	0.000014
22	H	3.256600	-3.042440	-0.000079
23	C	5.681830	-0.619350	0.000065
24	H	5.368440	1.520610	0.000119
25	H	5.727170	-2.773290	0.000012
26	H	6.762380	-0.540820	0.000113
27	N	2.503290	1.271080	-0.000017
28	N	1.550090	-0.766590	-0.000096
29	C	2.654300	2.716310	0.000121
30	H	1.668150	3.171720	-0.000254
31	H	3.199490	3.029450	0.891250
32	H	3.200190	3.029510	-0.890560

Photoswitch **8**, isomer *E*

- SMD(MTHF)/M06-2X/6-311+G(d,p)
- Imaginary frequencies: 0

Atomic type		Coordinates		
		X	Y	Z
1	C	2.638820	-0.713920	-0.205340
2	C	3.432530	0.420950	-0.037280
3	C	4.814850	0.323410	-0.005470
4	C	5.383550	-0.940960	-0.147860
5	C	4.597050	-2.083040	-0.316740
6	C	3.214130	-1.973970	-0.346050
7	C	0.984080	1.094520	-0.033300
8	H	5.438050	1.199740	0.123640
9	H	6.462770	-1.035520	-0.126670
10	H	5.069240	-3.050980	-0.425670
11	H	2.577790	-2.841890	-0.476720
12	C	1.203160	-0.423800	-0.206750
13	O	0.286570	-1.193710	-0.326940
14	S	2.521600	1.940590	0.124890
15	N	-0.117240	1.721100	-0.039470
16	C	-1.333420	1.034900	-0.133200
17	C	-3.230800	0.363730	-0.816170
18	C	-3.093570	-0.098270	0.504370
19	C	-4.383280	0.058630	-1.540780
20	C	-4.061100	-0.871440	1.138650
21	C	-5.357890	-0.709360	-0.918030
22	H	-4.504320	0.410180	-2.558590
23	C	-5.199150	-1.169790	0.400100
24	H	-3.931280	-1.227530	2.153750
25	H	-6.261410	-0.963860	-1.459270
26	H	-5.979810	-1.772370	0.848760
27	N	-1.870670	0.378080	0.938560
28	N	-2.110210	1.097420	-1.181320
29	C	-1.217790	-0.019950	2.171620
30	H	-0.889550	-1.059410	2.108880
31	H	-0.360620	0.624360	2.359610
32	H	-1.919130	0.091260	2.998650

Transition State between isomers **6***E* and **6***Z* (*Z*2)

- SMD(MTHF)/M06-2X/6-311+G(d,p)
- Imaginary frequencies: 1 (-106.1 cm^{-1})

Atomic type		Coordinates		
		X	Y	Z
1	6	-3.492699	1.857178	0.005805
2	6	-2.931926	3.165106	0.014027
3	6	-3.734018	4.317379	0.019305
4	6	-5.102145	4.135898	0.016683
5	6	-5.680647	2.844008	0.008950
6	6	-4.894936	1.711922	0.003468
7	6	-1.241393	1.698247	0.006560
8	1	-3.294029	5.308048	0.024259
9	1	-5.752200	5.003463	0.020266
10	1	-6.760369	2.751194	0.007054
11	1	-5.340793	0.723450	-0.002693
12	7	0.018076	1.278254	0.001955
13	6	1.189320	0.892089	-0.004790
14	6	1.622460	-0.577061	-0.006574
15	6	3.741426	0.552526	-0.016875
16	6	3.087190	-0.682825	-0.012654
17	6	5.130588	0.614612	-0.023113
18	6	3.812558	-1.872524	-0.014440
19	6	5.846281	-0.579174	-0.024816
20	1	5.646100	1.567625	-0.026479
21	6	5.198758	-1.818358	-0.020493
22	1	3.282937	-2.819012	-0.011014
23	1	6.929827	-0.542815	-0.029527
24	1	5.780944	-2.731682	-0.021903
25	16	2.669101	1.960918	-0.013785
26	8	0.819776	-1.477328	-0.002801
27	6	-2.407824	0.946034	0.001775
28	1	-2.438523	-0.132819	-0.004364
29	7	-1.575596	3.058829	0.015309
30	6	-0.653316	4.174933	0.020624
31	1	-0.826283	4.804007	0.895846
32	1	0.362649	3.787069	0.061681
33	1	-0.769509	4.770904	-0.886889

Transition State between isomers **6**Z2 and **6**Z1

- SMD(MTHF)/M06-2X/6-311+G(d,p)
- Imaginary frequencies: 1 (-45.9 cm^{-1})

Atomic type		Coordinates X	Y	Z
1	6	-3.306442	0.120288	-0.919504
2	6	-3.196884	0.018926	0.489334
3	6	-4.197982	-0.547081	1.281525
4	6	-5.333134	-1.012930	0.635776
5	6	-5.466740	-0.918479	-0.762125
6	6	-4.468203	-0.359191	-1.543049
7	6	-1.335703	0.972389	-0.277313
8	1	-4.090233	-0.622185	2.357704
9	1	-6.131372	-1.458732	1.218028
10	1	-6.368426	-1.292707	-1.233449
11	1	-4.580267	-0.292579	-2.619733
12	7	-0.073432	1.580478	-0.184828
13	6	0.953350	0.850215	-0.179876
14	6	2.366462	1.452616	-0.070741
15	6	2.791296	-0.902848	-0.155664
16	6	3.358228	0.368320	-0.058785
17	6	3.586547	-2.039010	-0.158705
18	6	4.738139	0.526448	0.037644
19	6	4.966955	-1.872147	-0.060882
20	1	3.152831	-3.028990	-0.235361
21	6	5.544063	-0.603910	0.036728
22	1	5.159818	1.522701	0.111873
23	1	5.603046	-2.749919	-0.061936
24	1	6.619898	-0.505116	0.111385
25	16	1.012746	-0.921927	-0.276345
26	8	2.559385	2.635675	-0.005975
27	6	-2.099990	0.738583	-1.389931
28	1	-1.826533	0.986351	-2.403521
29	7	-1.983346	0.552827	0.864577
30	6	-1.485336	0.609673	2.226698
31	1	-1.218749	-0.388764	2.582003
32	1	-0.606921	1.251953	2.262307
33	1	-2.252173	1.033283	2.877109

Transition State between isomers **7***E* and **7***Z*

- SMD(MTHF)/M06-2X/6-311+G(d,p)
- Imaginary frequencies: 1 (-134.6 cm^{-1})

Atomic type		Coordinates		
		X	Y	Z
1	6	-4.635832	0.155302	0.000000
2	6	-4.005880	-1.112143	0.000000
3	6	-4.730935	-2.308549	0.000000
4	6	-6.108072	-2.195726	0.000000
5	6	-6.754335	-0.937878	0.000000
6	6	-6.037462	0.241379	0.000000
7	6	-2.530092	0.536570	0.000000
8	1	-4.236040	-3.272074	0.000000
9	1	-6.712264	-3.095561	0.000000
10	1	-7.837508	-0.906572	0.000000
11	1	-6.526848	1.208389	0.000000
12	1	-1.899062	-1.491843	0.000000
13	7	-3.697028	1.154375	0.000000
14	7	-1.328396	1.108528	0.000000
15	6	-0.199250	1.600531	0.000000
16	6	1.109556	0.784475	0.000000
17	6	1.924445	3.036761	0.000000
18	6	2.269323	1.681845	0.000000
19	6	2.909342	4.015844	0.000000
20	6	3.605257	1.286936	0.000000
21	6	4.242286	3.611670	0.000000
22	1	2.649982	5.067927	0.000000
23	6	4.595192	2.259552	0.000000
24	1	3.850356	0.230459	0.000000
25	1	5.020121	4.366793	0.000000
26	1	5.639964	1.974378	0.000000
27	16	0.180696	3.365230	0.000000
28	8	1.095668	-0.419883	0.000000
29	7	-2.669494	-0.835434	0.000000

Transition State between isomers **8***E* and **8***Z*

- SMD(MTHF)/M06-2X/6-311+G(d,p)
- Imaginary frequencies: 1 (-118.5 cm^{-1})

Atomic type		Coordinates		
		X	Y	Z
1	6	4.586075	-0.298552	0.948298
2	6	4.190395	-1.006746	-0.207306
3	6	5.050349	-1.865417	-0.893888
4	6	6.339673	-1.978614	-0.397925
5	6	6.755830	-1.269371	0.747266
6	6	5.895072	-0.426726	1.429762
7	6	2.537421	0.197435	0.604748
8	1	4.728481	-2.410986	-1.773110
9	1	7.046456	-2.627382	-0.902180
10	1	7.773449	-1.392172	1.099482
11	1	6.210699	0.115662	2.313554
12	7	3.526200	0.419303	1.450525
13	7	1.315483	0.725328	0.697491
14	6	0.201221	1.247144	0.671612
15	6	-0.534459	1.726836	-0.602610
16	6	-2.096895	2.293506	1.121576
17	6	-1.830106	2.312265	-0.250202
18	6	-3.291177	2.800363	1.615588
19	6	-2.753981	2.840106	-1.149583
20	6	-4.208865	3.326970	0.709199
21	1	-3.506876	2.790084	2.677435
22	6	-3.949416	3.350186	-0.663598
23	1	-2.526155	2.844181	-2.209869
24	1	-5.144608	3.727297	1.082596
25	1	-4.681684	3.766057	-1.344502
26	16	-0.810107	1.591456	2.123546
27	8	-0.040735	1.604133	-1.693570
28	7	2.884260	-0.647370	-0.428521
29	6	2.025794	-1.137116	-1.490168
30	1	2.605489	-1.197063	-2.411533
31	1	1.629695	-2.124146	-1.242560
32	1	1.207710	-0.432713	-1.631552

10. References

1. J. E. Zweig and T. R. Newhouse, Isomer-Specific Hydrogen Bonding as a Design Principle for Bidirectionally Quantitative and Redshifted Hemithioindigo Photoswitches, *J. Am. Chem. Soc.*, 2017, **139**, 10956-10959.
2. V. Josef, F. Hampel and H. Dube, Heterocyclic Hemithioindigos: Highly Advantageous Properties as Molecular Photoswitches, *Angew. Chem. Int. Ed.*, 2022, **61**, e202210855.
3. M. W. H. Hoorens, M. Medved', A. D. Laurent, M. Di Donato, S. Fanetti, L. Slappendel, M. Hilbers, B. L. Feringa, W. J. Buma and W. Szymański, Iminothioindoxyl as a molecular photoswitch with 100 nm band separation in the visible range, *Nat. Commun.*, 2019, **10**, 2390.
4. M. E. Boëtius, M. W. H. Hoorens, M. Ošťadnický, A. D. Laurent, M. Di Donato, A. C. A. van Wingaarden, M. F. Hilbers, B. L. Feringa, W. J. Buma, M. Medved' and W. Szymański, Getting a molecular grip on the half-lives of iminothioindoxyl photoswitches, *Chem. Sci.*, 2024, **15**, 14379-14389.
5. J. Cosier and A. M. Glazer, A nitrogen-gas-stream cryostat for general X-ray diffraction studies, *J. Appl. Cryst.*, 1986, **19**, 105-107.
6. Y. Agilent (2014). CrysAlis PRO. Agilent Technologies Ltd, Oxfordshire, England.
7. G. M. Sheldrick, SHELXT - Integrated space-group and crystal-structure determination, *Acta Crystallogr., Sect. A*, 2015, **71**, 3-8.
8. G. M. Sheldrick, *Acta Crystallogr., Sect. A*, 2008, **64**, 112-122.
9. O. V. Dolomanov, L. J. Bourhis, R. J. Gildea, J. A. K. Howard and H. Puschmann, OLEX2: a complete structure solution, refinement and analysis program, *J. Appl. Cryst.*, 2009, **42**, 339-341.
10. Y. Zhao and D. G. Truhlar, The M06 suite of density functionals for main group thermochemistry, thermochemical kinetics, noncovalent interactions, excited states, and transition elements: two new functionals and systematic testing of four M06-class functionals and 12 other functionals, *Theor. Chem. Acc.*, 2008, **120**, 215-241.
11. N. Mardirossian and M. Head-Gordon, Thirty years of density functional theory in computational chemistry: an overview and extensive assessment of 200 density functionals, *Mol. Phys.*, 2017, **115**, 2315-2372.
12. A. V. Marenich, C. J. Cramer and D. G. Truhlar, Universal Solvation Model Based on Solute Electron Density and on a Continuum Model of the Solvent Defined by the Bulk Dielectric Constant and Atomic Surface Tensions, *J. Phys. Chem. B*, 2009, **113**, 6378-6396.
13. C. Adamo and V. Barone, Exchange functionals with improved long-range behavior and adiabatic connection methods without adjustable parameters: The *m*PW and *m*PW1PW models, *J. Chem. Phys.*, 1998, **108**, 664-675.
14. C. Bonini, M. Funicello and P. Spagnolo, Novel α-Carboline Synthesis Using Tandem aza-Wittig-Electrocyclization Reaction of Indol-2-yl Phosphorane with Enone, *Synlett*, 2006, 1574-1576.
15. P. Molina, A. Lorenzo and E. Aller, Iminophosphorane-Mediated Annulation of 1,3,5-Triazine to Benzimidazole: Synthesis of 1,3,5-Triazino[1,2-*a*]benzimidazoles, *Synthesis*, 1992, 297-302.
